# Astrocyte-mediated spike-timing-dependent long-term depression modulates synaptic properties in the developing cortex

**DOI:** 10.1371/journal.pcbi.1008360

**Published:** 2020-11-10

**Authors:** Tiina Manninen, Ausra Saudargiene, Marja-Leena Linne

**Affiliations:** 1 Faculty of Medicine and Health Technology, Tampere University, Tampere, Finland; 2 Department of Neurobiology, Stanford University, Stanford, CA, USA; 3 Neuroscience Institute, Lithuanian University of Health Sciences, Kaunas, Lithuania; 4 Department of Informatics, Vytautas Magnus University, Kaunas, Lithuania; University College London, UNITED KINGDOM

## Abstract

Astrocytes have been shown to modulate synaptic transmission and plasticity in specific cortical synapses, but our understanding of the underlying molecular and cellular mechanisms remains limited. Here we present a new biophysicochemical model of a somatosensory cortical layer 4 to layer 2/3 synapse to study the role of astrocytes in spike-timing-dependent long-term depression (t-LTD) *in vivo*. By applying the synapse model and electrophysiological data recorded from rodent somatosensory cortex, we show that a signal from a postsynaptic neuron, orchestrated by endocannabinoids, astrocytic calcium signaling, and presynaptic N-methyl-D-aspartate receptors coupled with calcineurin signaling, induces t-LTD which is sensitive to the temporal difference between post- and presynaptic firing. We predict for the first time the dynamics of astrocyte-mediated molecular mechanisms underlying t-LTD and link complex biochemical networks at presynaptic, postsynaptic, and astrocytic sites to the time window of t-LTD induction. During t-LTD a single astrocyte acts as a delay factor for fast neuronal activity and integrates fast neuronal sensory processing with slow non-neuronal processing to modulate synaptic properties in the brain. Our results suggest that astrocytes play a critical role in synaptic computation during postnatal development and are of paramount importance in guiding the development of brain circuit functions, learning and memory.

## Author summary

Brain development is dependent on neuroplasticity, the ability of the brain to modify its structure and function. Experimental evidence suggests that astrocytes, the non-neuronal cells in the brain, take part in shaping synaptic plasticity. In this study, we built a new computational model of spike-timing-dependent long-term depression and addressed the involvement of astroglial cells in modulation of synaptic glutamate transmission. Our results suggest that astrocytes are an integral part of synaptic computations and may guide brain circuit functions, learning and memory during postnatal development. Disruptions in these processes are likely involved in neurodevelopmental diseases such as schizophrenia and autism spectrum disorder. Modeling synaptic functions may help develop pharmacological targets for treatments of brain disorders.

## Introduction

Synaptic long-term plasticity, defined as the activity-dependent change in the strength or efficacy of the synaptic connection between a pre- and postsynaptic neuron, is expressed in the brain in diverse forms across multiple timescales [1]. Action potential (or spike) timing is one of the many factors governing synaptic plasticity induction [2, 3]. In spike-timing-dependent plasticity (STDP), the order and precise temporal difference between pre- and postsynaptic action potentials determine the direction and magnitude of long-term plasticity. Depending on the form of synaptic plasticity and the brain area, a large number of cellular and molecular level mechanisms are involved [4–7]. In the developing mouse barrel area of the somatosensory cortex, spike-timing-dependent long-term depression (t-LTD) [8] at layer 4 (L4) to layer 2/3 (L2/3) synapses has been shown to require activation of presynaptic mechanisms [9–14] and involve astrocytic functions [15]. This t-LTD has been shown to emerge in the first postnatal week, be present during the second week, and disappear in the adult, whereas spike-timing-dependent long-term potentiation (t-LTP) persisted into adulthood [10]. Long-term depression may provide an important mechanism for synapse pruning and subsequent neuron and circuit remodeling during postnatal development [16].

Astrocytes, a type of non-neuronal cells in the mammalian brain, are recognized as important homeostatic, metabolic, and neuromodulatory elements that are also coupled to the neurovascular system [17, 18]. In the developing central nervous system, astrocytes promote the formation of excitatory synapses and the establishment of synaptic connectivity [19]. Astrocytes can also sense and modulate synaptic functions [20]. Astrocytes maintain glutamatergic synaptic transmission by glutamate uptake [21] and clear excess extracellular potassium ions (K^+^) to spatially transfer K^+^ from regions of elevated concentration to regions of lower concentration [22]. In addition, there is ample evidence to indicate that astrocytes actively contribute to the information processing capabilities of neural circuits and ultimately affect animal behavior [23, 24]. Astrocytes have, for example, been shown to influence brain state transitions [25], promote the coordinated activation of neuronal networks [26], and modulate sensory-evoked neuronal network activity [27] and brain rhythms during sleep [28]. Recent research has the potential to revolutionize our current understanding of the role of astrocytes in the modulation of brain network activity [17, 29].

Astrocytes are integral elements of synapses in developing rodent and human cerebral cortices [30–32]. A single cortical astrocyte is estimated to contact 20,000 to 120,000 synapses in rodents and up to 2,000,000 synapses in humans [30]. Several lines of evidence suggest that, through this close association with neurons, astrocytes alter synaptic functions. Astrocytes have been shown to modulate synaptic transmission [33, 34], long-term potentiation [34–40], and long-term depression [15] in several brain areas, as well as provide a developmental switch of synaptic transmission from LTD to LTP in hippocampus [41]. More and more details about astrocytic cellular and subcellular mechanisms have recently been presented [40, 42–49]. It is of interest to understand how these subcellular mechanisms in astrocytes and their processes are linked with synaptic transmission and plasticity in neocortex [13, 15, 42, 50, 51]. In the developing somatosensory cortex, t-LTD has been shown to depend on type 1 cannabinoid receptor (CB_1_R) activation and increased astrocytic calcium (Ca^2+^) signaling [15]. Nevertheless, the central questions still remain: Do cortical astrocytes *in vivo* have subcellular mechanisms capable of synapse modification at fast enough timescales comparable to neuronal ones? Does this modulation depend on the brain area and circuitry in question? Is this modulation significant only in developing brain circuits or does it also happen in mature circuits? The answers to these questions will significantly increase our understanding of mammalian neocortical network functioning.

Computational modeling is an important complementary method for linking the dynamics of different biochemical and biophysical reactions and processes together and for unraveling the complexity of synaptic functions. Our goal here is to better understand through computational modeling the role of cortical astrocytes in sensory processing, particularly in synaptic plasticity, during postnatal development. To address this question we propose a new biophysicochemical model of a somatosensory cortical L4 to L2/3 synapse and study the role of astrocytes in t-LTD *in vivo*. We made several assumptions based on the experimental electrophysiological, Ca^2+^ imaging, and other data (see Materials and methods and S1 Appendix). The computational model was built in component-by-component manner for the presynaptic L4 spiny stellate cell and postsynaptic L2/3 pyramidal cell as well as for the nearby fine astrocyte process. After careful validation of each model component, all the components were brought together to describe all the necessary elements of a somatosensory cortical synapse. The integrated model takes into account the well-established biophysical and biochemical mechanisms for this particular synapse, such as the voltage-gated ion channels, transmitter-gated receptors, Ca^2+^-mediated signaling pathways including the neuronal endocannabinoid and astrocytic inositol 1,4,5-trisphosphate (IP_3_) receptor (IP_3_R) signaling, as well as other crucial subcellular mechanisms. These mechanisms are described using deterministic differential equations. The integrated model is carefully validated against experimental data on synaptic plasticity [11, 12, 15]. Here we show that cortical astrocytic Ca^2+^ dynamics can be modified by presynaptic L4 spiny stellate cell and postsynaptic L2/3 pyramidal cell activity through the endocannabinoid signaling pathway. The subsequent downstream signaling pathways in astrocytes have an influence on synaptic long-term plasticity, particularly on the t-LTD in somatosensory cortex, through presynaptic N-methyl-D-aspartate receptors (NMDARs) and calcineurin (CaN) signaling. Our study provides several predictions that can be tested in future electrophysiological, Ca^2+^ imaging, and molecular biology experiments.

## Results

We simulated a synapse model containing neuronal pre- and postsynaptic terminals and a fine astrocyte process. Specifically, our computational model includes the axonal compartment of a presynaptic L4 spiny stellate cell, the dendritic and somatic compartments of a postsynaptic L2/3 pyramidal cell, and the nearby fine astrocyte process. Several previous modeling studies [52–55] have had an influence on our synapse modeling project and the choices we made during the work. In our *in silico* experiments, we studied which mechanisms are important in the induction of t-LTD at L4-L2/3 synapses in somatosensory cortex, including key Ca^2+^-dependent intracellular processes. We used stimulation protocols equivalent to the protocols applied in electrophysiological experiments *in vitro* and *in vivo* to activate our *in silico* synapse model [12]. We showed that t-LTD at an L4-L2/3 synapse can be explained by the activation of Ca^2+^-dependent mechanisms in the fine astrocyte process and this further has an influence on the probability of neurotransmitter release in the presynaptic neuron through NMDARs and calcineurin signaling. In the absence of the Ca^2+^-dependent mechanism in the fine astrocyte process, the synapse did not show t-LTD similarly to experimental data.

### Synapse model components

**Fig 1:**
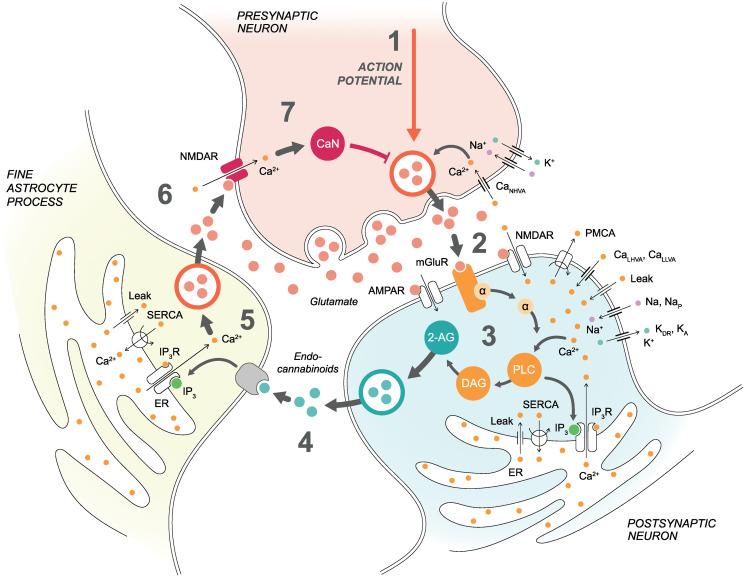
Schematic illustration of the synapse model. Pre- and postsynaptic neurons and a fine astrocyte process are presented with key model components. (1) Presynaptic membrane potential depends on currents via Ca_NHVA_, Na^+^, and K^+^ channels as well as via NMDARs. Presynaptic action potential and Ca_NHVA_- and NMDAR-mediated Ca^2+^ concentrations together with the influence of CaN affect the vesicular release. (2) The released glutamate in the synaptic cleft activates postsynaptic mGluRs, NMDARs, and AMPARs in addition to presynaptic NMDARs. (3) Postsynaptic membrane potential in the soma depends on currents via Na^+^, Na_P_, and K_DR_ channels, whereas postsynaptic membrane potential in the dendrite depends on currents via Ca_LHVA_, Ca_LLVA_, Na^+^, and K_A_ channels as well as via NMDARs and AMPARs. The activation of postsynaptic mGluRs and NMDARs, together with the Ca_LHVA_- and Ca_LLVA_-mediated Ca^2+^ influx, triggers a G-protein signaling cascade where GαGTP dissociates from mGluR-bound Gβγ and activates PLC and production of DAG and IP_3_. Increases in Ca^2+^ and IP_3_ concentrations activate Ca^2+^ release via IP_3_Rs from the ER to the cytosol. On the other hand, PMCA and SERCA pumps transfer Ca^2+^ away from the cytosol and leak fluxes transfer Ca^2+^ back to the cytosol. The production of DAG leads to a production of endocannabinoid 2-AG. (4) Endocannabinoid 2-AG released from the postsynaptic neuron binds to the astrocytic CB_1_Rs and triggers Ca^2+^ signaling in the astrocyte. We modeled this step by directly modifying astrocytic IP_3_ concentration based on the postsynaptic 2-AG concentration. (5) Astrocytic IP_3_ and Ca^2+^ activate similar ER-related events as in the postsynaptic neuron. Astrocytic Ca^2+^ increase then induces glutamate exocytosis to the extrasynaptic space. (6) Glutamate in the extrasynaptic space and the spillover of glutamate from the synaptic cleft activate presynaptic NMDARs. (7) Presynaptic NMDAR-mediated Ca^2+^ concentration activates CaN, and CaN has an effect on vesicular release together with presynaptic action potential and Ca_NHVA_-mediated Ca^2+^ concentration.

Our specific goal was to study the role of astrocytes in the modulation of t-LTD. We selected and modeled some of the most important candidate signaling pathways that may be crucial in explaining signaling in synapses, specifically the signaling from a presynaptic neuron to a postsynaptic neuron, from the postsynaptic neuron to an astrocyte, as well as from the astrocyte to the presynaptic neuron (Fig 1). We extended a previously published presynaptic one-compartmental neuron model [56] by adding (1) high-voltage-activated (HVA) N-type Ca^2+^ (Ca_NHVA_) channels [57], (2) NMDARs composed of GluN1 and either GluN2C or GluN2D subunits (GluN2C/D-containing NMDARs) [58, 59], (3) Ca^2+^ signaling [57], (4) calcineurin signaling [60], (5) calcineurin-dependence to available glutamate release, and (6) modified the known equations of glutamate release to the synaptic cleft [61–65]. We modified a previously published postsynaptic two-compartmental neuron model [66] by adopting (1) A-type K^+^ (K_A_), delayed rectifier K^+^ (K_DR_), sodium (Na^+^), and persistent Na^+^ (Na_P_) channels [67], (2) L-type HVA Ca^2+^ (Ca_LHVA_) channels [54, 68], (3) low-voltage-activated (LVA) L-type Ca^2+^ (Ca_LLVA_) channels [69], (4) α-amino-3-hydroxy-5-methyl-4-isoxazolepropionic acid receptors (AMPARs) [70], (5) NMDARs composed of GluN1 and GluN2B subunits (GluN2B-containing NMDARs) [70], (6) metabotropic glutamate receptor (mGluR) activation to endocannabinoid release [53, 71], and (7) Ca^2+^ signaling including, for example, plasma membrane Ca^2+^-ATPase (PMCA), sarco/endoplasmic reticulum (ER) Ca^2+^-ATPase (SERCA), and IP_3_R models [72–74]. For the astrocyte model, we utilized previously published [72, 73, 75] and extensively tested [76–79] Ca^2+^ signaling models, including IP_3_Rs and SERCA pumps on the ER membrane, and added a modified version of a previously published model for IP_3_-dependence on endocannabinoids [80] and a model for Ca^2+^-dependent glutamate exocytosis to the extrasynaptic space [61–65]. In summary, we combined previously published validated model components with novel components developed in this study to create a new synapse model.

### Synapse model dynamics before, during, and after t-LTD induction: Fitting the model to experimental data

**Fig 2:**
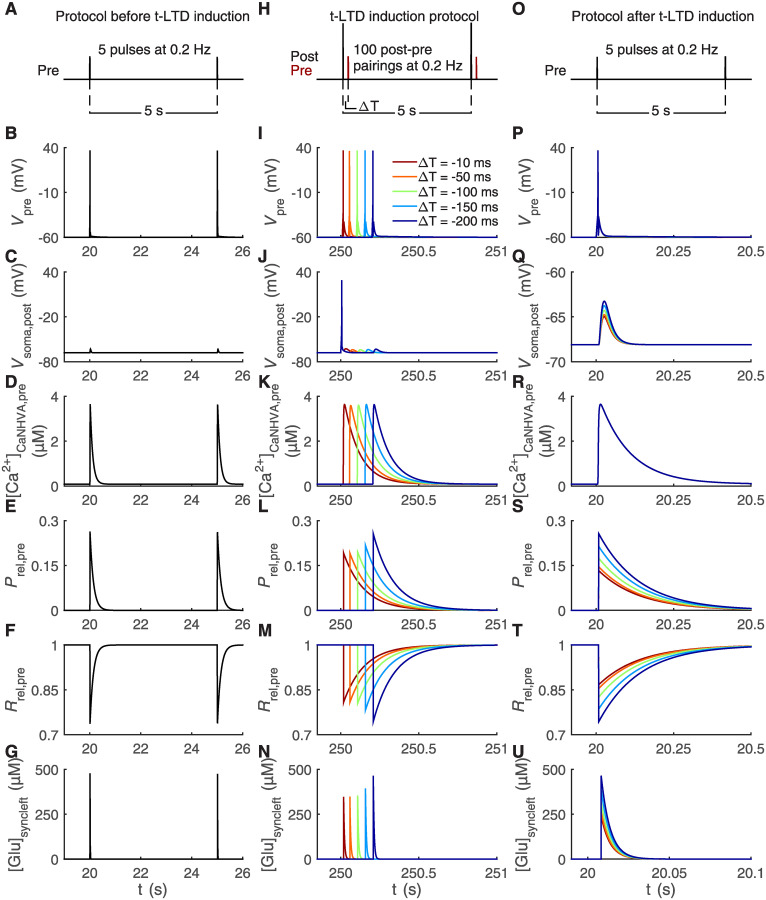
Pre- and postsynaptic neurons respond to t-LTD stimulation protocols through reduced synaptic glutamate release. The t-LTD stimulation protocols consisted of the protocol before t-LTD induction composed of five presynaptic pulses at a frequency of 0.2 Hz illustrated in (A), the t-LTD induction protocol with 100 post-pre pairings at a frequency of 0.2 Hz having the temporal difference ΔT as values between −10 ms and −200 ms illustrated in (H), and the protocol after t-LTD induction composed of five presynaptic pulses at a frequency of 0.2 Hz illustrated in (O) [12]. The simulation results are shown for six key model variables during the first two stimulus pulses of our protocol before t-LTD induction in (B–G), during a single post-pre pairing with five different ΔT occurring about in the middle of the 100 post-pre pairings of the t-LTD induction protocol in (I–N), and during a single stimulus pulse of our protocol after t-LTD induction in (P–U, note the different x-axis in U). The presynaptic membrane potential (*V*_pre_) in (B, I, P), postsynaptic membrane potential in the soma (*V*_soma,post_) in (C, J, Q, note the different y-axis in Q), presynaptic Ca_NHVA_-mediated Ca^2+^ concentration ([Ca^2+^]_CaNHVA,pre_) in (D, K, R), release probability of presynaptic glutamate vesicles (*P*_rel,pre_) in (E, L, S), fraction of releasable presynaptic vesicles (*R*_rel,pre_) in (F, M, T), and glutamate concentration in synaptic cleft ([Glu]_syncleft_) in (G, N, U) responded to the stimuli shown in (A, H, O), respectively. In (J), the postsynaptic action potential in the soma was followed by an EPSP with a delay corresponding to ΔT. The lowest release probability of presynaptic glutamate vesicles in (L, S), the lowest glutamate concentration in synaptic cleft in (N, U), and the highest fraction of releasable presynaptic vesicles in (M, T) were obtained with the shortest ΔT due to the astrocyte-mediated signaling during depression.

**Fig 3:**
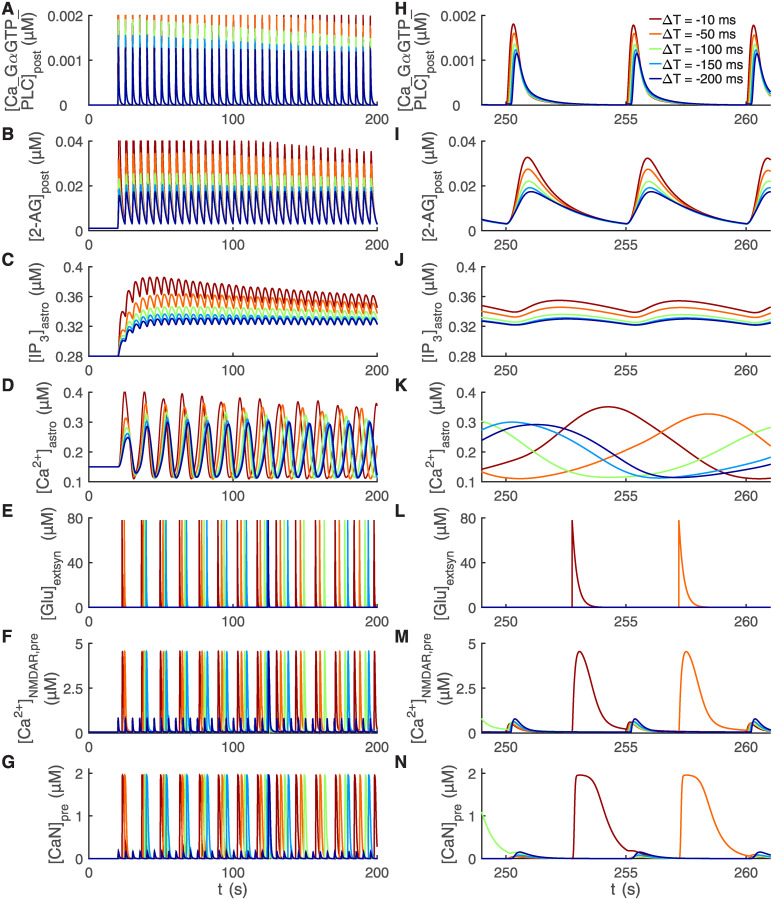
Delayed activation of astrocytic Ca^2+^ signaling by postsynaptic endocannabinoids is followed by fast astrocytic glutamate release and presynaptic Ca^2+^ and calcineurin activation. The stimulation protocol used was the t-LTD induction protocol with five different temporal differences ΔT [12] (see Fig 2H). The simulation results are shown for seven key model variables during the first 200 s of the stimulation protocol in (A–G) and during three post-pre pairings occurring about in the middle of the whole stimulation protocol in (H–N). The shorter the temporal difference ΔT, the higher the concentration of postsynaptic Ca^2+^-GαGTP-PLC complex ([Ca_GαGTP_PLC]_post_) in (A, H), postsynaptic 2-AG concentration ([2-AG]_post_) in (B, I), astrocytic IP_3_ concentration ([IP_3_]_astro_) in (C, J), and astrocytic Ca^2+^ concentration ([Ca^2+^]_astro_) in (D, K). The postsynaptic 2-AG concentration shown in (B, I) triggered an increase in the astrocytic IP_3_ concentration shown in (C, J) which was followed by an increase in the astrocytic Ca^2+^ concentration shown in (D, K). (E, L) After the astrocytic Ca^2+^ concentration reached the threshold, astrocyte released a fixed amount of glutamate to the extrasynaptic space and this can be seen as an increase in the glutamate concentration in the extrasynaptic space ([Glu]_extsyn_). (F, M) Both glutamate in the extrasynaptic space and the spillover of glutamate from the synaptic cleft were able to activate presynaptic NMDARs. The smaller changes in the presynaptic NMDAR-mediated Ca^2+^ concentration ([Ca^2+^]_NMDAR,pre_) occurred due to the spillover of glutamate from the synaptic cleft, and the larger changes occurred due to the glutamate in the extrasynaptic space. (G, N) The presynaptic NMDAR-mediated Ca^2+^ influx increased the presynaptic calcineurin concentration ([CaN]_pre_).

In our simulations, we closely followed experimental stimulation protocols [12]. During our stimulation protocol before t-LTD induction, we simulated our synapse model with five pulses of presynaptic stimulus at a frequency of 0.2 Hz (Fig 2A). Our t-LTD induction protocol consisted of 100 post-pre pairings at a frequency of 0.2 Hz where a postsynaptic stimulus occurred between 10 ms and 200 ms before a presynaptic stimulus, thus the temporal difference (ΔT) had values between −10 ms and −200 ms (Fig 2H). The protocol after t-LTD induction included five pulses of presynaptic stimulus at a frequency of 0.2 Hz (Fig 2O), similarly as with the protocol before t-LTD induction. All these different stimuli triggered changes in the pre- and postsynaptic membrane potentials, similarly to experimental data [12, 15] (Fig 2B, 2C, 2I, 2J, 2P and 2Q), that led, for example, to the opening of pre- and postsynaptic Ca^2+^ channels and glutamate release from the presynaptic neuron (Fig 2D–2G, 2K–2N and 2R–2U). The simulated presynaptic Ca^2+^ concentration values followed the experimental values [81–84] (Figs 2D, 2K and 2R and 3F and 3M). The glutamate concentration in the synaptic cleft increased to about 500 μM after stimuli, which is close to the measured experimental values [85] (Fig 2G, 2N and 2U). The release probability of presynaptic glutamate vesicles and the concentration of glutamate in the synaptic cleft were the lowest for the shortest ΔT due to ongoing astrocyte-mediated molecular dynamics during depression (Fig 2L, 2N, 2S and 2U). The effect of depression is clearly seen after t-LTD induction (Fig 2Q and 2S–2U).

During the t-LTD induction protocol, the released glutamate in the synaptic cleft activated AMPARs, NMDARs, and mGluRs in the dendritic membrane of the postsynaptic neuron, in addition to presynaptic NMDARs. The activation of these postsynaptic receptors together with Ca^2+^ influx via Ca_LHVA_ and Ca_LLVA_ channels into the postsynaptic neuron induced a G-protein signaling cascade that activated phospholipase C (PLC) (Fig 3A and 3H). This led to the production of biologically realistic concentrations of diacylglycerol (DAG) and IP_3_ in the postsynaptic neuron. Then IP_3_ activated, together with Ca^2+^, Ca^2+^-induced Ca^2+^ release via IP_3_Rs from the ER to the cytosol in the postsynaptic neuron, which led to an increase in Ca^2+^ concentration in the cytosol. The production of DAG, on the other hand, resulted in a production of 2-arachidonoylglycerol (2-AG) (Fig 3B and 3I), and ultimately in the release of endocannabinoid 2-AG from the postsynaptic neuron. All these are well-established signaling pathways known to exist in cortical neurons.

Endocannabinoid 2-AG can bind to the astrocytic CB_1_Rs and trigger Ca^2+^ signaling in the astrocyte [15, 42]. We modeled this step by directly modifying IP_3_ concentration in the astrocyte based on the postsynaptic concentration of 2-AG (Fig 3B, 3C, 3I and 3J), followed by an increase in astrocytic Ca^2+^ concentration (Fig 3D and 3K) and ultimately glutamate exocytosis, thus inducing glutamate release from the astrocyte to the extrasynaptic space (Fig 3E and 3L). We chose the astrocytic Ca^2+^ threshold for glutamate release based on experimental data [86]. Similarly, we chose the maximum value of glutamate concentration in the extrasynaptic space based on experimental findings [87].

Astrocytes have been shown to have an effect on presynaptic glutamate release by modifying release probabilities [15, 36, 88]. In somatosensory cortex, astrocytes have exhibited reduction in the presynaptic release probabilities as a response to the t-LTD induction protocol [15]. In our synapse model, glutamate release from the presynaptic neuron depended, among other things, on the presynaptic Ca_NHVA_- and NMDAR-mediated Ca^2+^ concentrations (Figs 2D, 2K and 2R and 3F and 3M), release probability of presynaptic glutamate vesicles (Fig 2E, 2L and 2S), presynaptic calcineurin concentrations [14] (Fig 3G and 3N), and fraction of presynaptic glutamate release inhibition (*f*_pre_, see Materials and methods and S1 Appendix). The presynaptic NMDARs were activated by the glutamate in the extrasynaptic space and the spillover of glutamate from the synaptic cleft. Our simulations showed that the glutamate in the extrasynaptic space substantially increased the presynaptic NMDAR-mediated Ca^2+^ concentration (Fig 3F and 3M).

### t-LTD amplitude depends on the temporal difference between post- and presynaptic activity: Confirming the broad t-LTD time window

**Fig 4:**
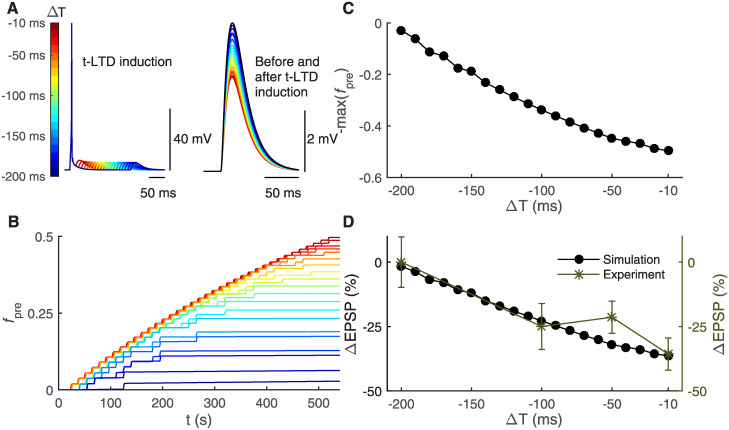
Shorter temporal difference between pre- and postsynaptic activity leads to stronger t-LTD through astrocyte-mediated cellular and subcellular mechanisms. The t-LTD stimulation protocols were obtained from experimental literature [12] and their use in *in silico* modeling is shown in Fig 2A, 2H and 2O. (A) In the left, the postsynaptic membrane potential in the soma is shown during a single post-pre pairing of the t-LTD induction protocol with a temporal difference ΔT between −10 ms and −200 ms at every 10 ms. The postsynaptic stimulus evoked a somatic action potential followed by an EPSP generated by the presynaptic stimulus. The longer the temporal difference ΔT, the longer the delay for the EPSP. In the right, the postsynaptic membrane potential in the soma, in other words in this case the postsynaptic EPSP generated by the presynaptic stimulus, is shown during a single presynaptic stimulus occurring before (black) and after (color bar) t-LTD induction. The shorter the temporal difference ΔT, the smaller the amplitude of the EPSP. (B) The fraction of presynaptic glutamate release inhibition (*f*_pre_) had the highest values with the shortest ΔT, i.e. the strongest t-LTD, during the 100 post-pre pairings in the t-LTD induction protocol. Color bar is given in (A). (C) The final value of −*f*_pre_ is shown as a function of ΔT. (D) The ΔEPSP percentage is shown as a function of ΔT. We calculated the ΔEPSP percentage for every ΔT as the percentage change between the somatic EPSP amplitude evoked by the presynaptic stimulus occurring before t-LTD induction (shown in (A, right) as black) and the somatic EPSP amplitude evoked by the presynaptic stimulus occurring after t-LTD induction (shown in (A, right) with different colors given in the color bar). Our synapse model confirmed the experimental data [12]. The shorter the temporal difference ΔT, the stronger the t-LTD.

In our *in silico* experiments, we followed the experimental t-LTD stimulation protocols [12]. First, we estimated the amplitude of the excitatory postsynaptic potential (EPSP) before t-LTD induction when the stimulation protocol consisted of only a presynaptic stimulus repeated five times at a frequency of 0.2 Hz (Fig 2A). The EPSP before t-LTD induction is presented in Figs 2C and 4A (right). Then t-LTD was induced by the post-pre pairing protocol consisting of a postsynaptic stimulus followed by a presynaptic stimulus with a temporal difference ΔT from −10 ms to −200 ms and the pairing was repeated 100 times at a frequency of 0.2 Hz (Fig 2H). In this case, the postsynaptic action potential in the soma was followed by an EPSP which is shown during one presynaptic stimulus in Fig 4A (left) for ΔT from −10 ms to −200 ms (see also Fig 2J). Presynaptic activity and thus EPSPs were delayed by ΔT in respect to the postsynaptic action potential. After t-LTD induction, we estimated the changes in the EPSPs by stimulating the synapse model with the same protocol as before t-LTD induction, including only a presynaptic stimulus repeated five times at a frequency of 0.2 Hz (Figs 2O and 2Q and 4A (right)).

The post-pre pairings induced presynaptically expressed t-LTD, sensitive to the temporal difference between the pre- and postsynaptic activity (ΔT). The strongest LTD change was observed for the shortest ΔT: the EPSP decreased from 4.9 mV (before t-LTD induction) to 3.1 mV (after t-LTD induction) (Fig 4A (right)). The time courses of the presynaptic glutamate release inhibition fraction (*f*_pre_) for ΔT from −10 ms to −200 ms are shown in Fig 4B. A shorter ΔT led to a larger increase in *f*_pre_ (Fig 4B), and thus stronger t-LTD (Fig 4D). The dependence of final *f*_pre_ values on ΔT is shown in Fig 4C. The fraction *f*_pre_ had different resulting values depending on ΔT used (Fig 4C), for example *f*_pre_ = 0.5 for ΔT = −10 ms, *f*_pre_ = 0.34 for ΔT = −100 ms, and *f*_pre_ = 0.03 for ΔT = −200 ms. The changes in the EPSP, estimated in Fig 4A (right), showed similar dependence on ΔT: for example, ΔEPSP = −36.45% for ΔT = −10 ms, ΔEPSP = −22.86% for ΔT = −100 ms, and ΔEPSP = −1.64% for ΔT = −200 ms (Fig 4D). Thus the stimulation protocol induced t-LTD for ΔT values shorter than −200 ms and t-LTD was the strongest for the shortest ΔT, which was consistent with the experimental results [12] (Fig 4D). The broad time window for t-LTD in somatosensory cortex has been reported in several experimental studies [8, 12].

### t-LTD requires astrocytic signaling and presynaptic NMDARs

**Fig 5:**
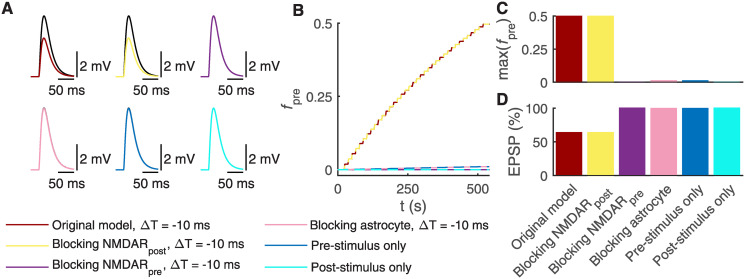
Blocking astrocytic Ca^2+^ signaling and presynaptic NMDARs prevents t-LTD induction. (A) The postsynaptic EPSPs in the soma are shown before (black) and after t-LTD induction (other colors than black) when manipulating postsynaptic NMDAR, presynaptic NMDAR, or astrocytic signaling, or stimulation protocols (Fig 2A, 2H and 2O). In top left, the post-pre pairing protocol with the temporal difference ΔT = −10 ms induced t-LTD (same synapse model as in Fig 4). In top middle, blocking the postsynaptic NMDARs failed to prevent t-LTD with the post-pre pairing protocol for ΔT = −10 ms. In top right, blocking the presynaptic NMDAR, on the other hand, prevented t-LTD with the post-pre pairing protocol for ΔT = −10 ms. In bottom left, blocking the fine astrocyte process also prevented t-LTD with the post-pre pairing protocol for ΔT = −10 ms. In bottom middle, the presynaptic stimulus at a frequency of 0.2 Hz for 500 s failed to induce t-LTD. In bottom right, the postsynaptic stimulus at a frequency of 0.2 Hz for 500 s failed to induce t-LTD. (B) The fraction of presynaptic glutamate release inhibition (*f*_pre_) is shown during the whole t-LTD induction protocol for all six models described in (A). (C) The values of *f*_pre_ at the end of the t-LTD induction protocol are shown for all six models described in (A). The high values of *f*_pre_ led to t-LTD. (D) The EPSP percentage is given for all six models described in (A). We calculated the EPSP percentage for every ΔT by normalizing the EPSP amplitude occurring after t-LTD induction by the EPSP amplitude occurring before t-LTD induction, and multiplied them by 100%.

Previous experimental studies have reported that presynaptic GluN2C/D-containing NMDARs are required for t-LTD, whereas postsynaptic GluN2B-containing NMDARs are necessary for t-LTP at the vertical L4 input onto L2/3 neuron [9, 12, 15, 89–91]. Our model simulations showed that blocking postsynaptic GluN2B-containing NMDARs, thus changing their conductance to zero, did not prevent the increase in fraction of presynaptic glutamate release inhibition (*f*_pre_) (Fig 5B and 5C), and therefore did not abolish t-LTD (Fig 5A (top middle) and Fig 5D) when the same t-LTD induction protocol was used as in Fig 2H with a temporal difference ΔT equaling −10 ms. For a comparison, Fig 5A (top left) shows the original synapse model with a temporal difference ΔT equaling −10 ms. Blocking presynaptic GluN2C/D-containing NMDARs failed to increase *f*_pre_ (Fig 5B and 5C) and prevented t-LTD (Fig 5A (top right) and Fig 5D), following the same t-LTD induction protocol. Thus, our computational synapse model confirmed the experimental findings that presynaptic NMDARs, but not postsynaptic NMDARs, are necessary for t-LTD induction.

Previous experimental studies have also shown that t-LTD requires astrocytic CB_1_R activation by neuronal endocannabinoid release followed by an increase in astrocytic Ca^2+^ signaling and the exocytosis of glutamate from astrocytes [15]. The released glutamate then activates presynaptic NMDARs and leads to t-LTD [15]. We therefore tested whether interfering with the astrocytic activity leads to the inhibition of t-LTD by blocking the astrocyte, thus keeping the astrocyte model in a steady state by setting all the astrocytic differential equations to zero in our synapse model. The simulation results showed that *f*_pre_ stayed at low levels (Fig 5B and 5C) and did not lead to t-LTD (Fig 5A (bottom left) and Fig 5D). Thus, our synapse model confirmed the experimental findings that blocking the fine astrocyte process activity entirely prevented t-LTD.

In addition, we tested the model by applying either a presynaptic (Fig 5A (bottom middle)) or a postsynaptic (Fig 5A (bottom right)) stimulus at a frequency of 0.2 Hz for 500 s, thus repeating both stimulation protocols 100 times. In both cases, *f*_pre_ did not increase substantially, failing to induce t-LTD (Fig 5D). Our synapse model confirmed the experimental data that an unpaired synaptic pathway remains unmodified [12].

### Astrocytes sense the temporal difference of t-LTD and modify their Ca^2+^ signaling

**Fig 6:**
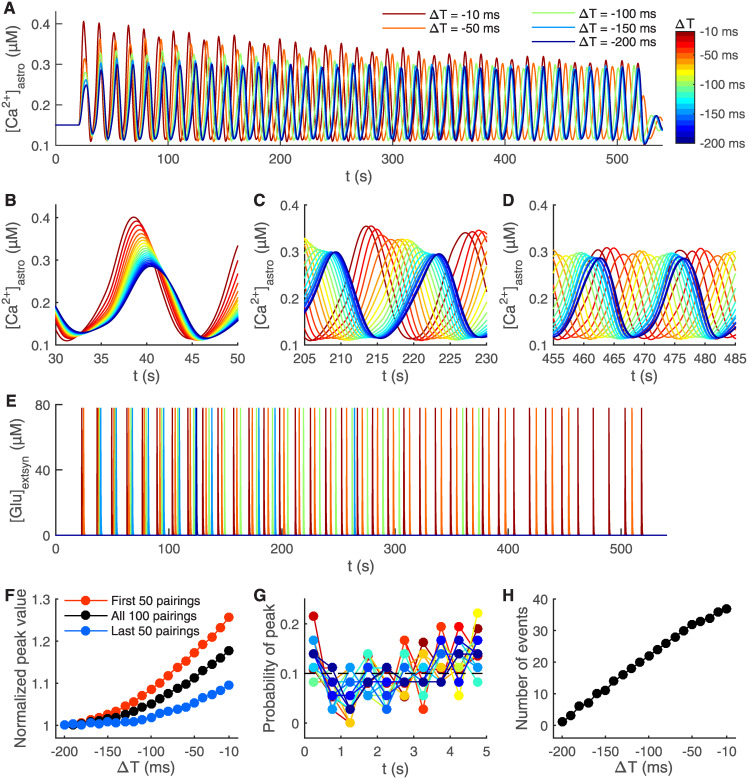
Shorter temporal difference between pre- and postsynaptic activity leads to shorter delay in astrocytic Ca^2+^ response and more frequent glutamate release from astrocyte. The stimulation protocol used was the t-LTD induction protocol for every temporal difference ΔT between −10 ms and −200 ms at every 10 ms [12] (Fig 2H). The astrocytic Ca^2+^ concentration ([Ca^2+^]_astro_) is shown during the whole simulation of the 100 post-pre pairings for five different ΔT in (A), and with twenty different ΔT in the beginning of the simulation in (B), in the middle of the simulation in (C), and in the end of the simulation in (D). Color bar is given in (A). (E) The glutamate concentration in the extrasynaptic space ([Glu]_extsyn_) is shown for the whole simulation for five different ΔT. Glutamate was released every time the astrocytic Ca^2+^ concentration reached the threshold. (F) The normalized mean peak values of astrocytic Ca^2+^ concentration are shown during the first 50 post-pre pairings, last 50 post-pre pairings, and the whole 100 post-pre pairings of the t-LTD induction protocol with different ΔT. (G) The probability of astrocytic Ca^2+^ peaks is shown as a function of time between each post-pre pairing. Every 5 s long sweep between each of the post-pre pairings was divided into ten 0.5 s long bins. The time for the post-pre pairing was in the beginning of the 5 s long sweep. The probability of astrocytic Ca^2+^ peaks was calculated for every bin during the whole t-LTD induction protocol with different ΔT. The dashed line indicates the equal probability between the ten bins, so 0.1. Similarly to experimental data [15], Ca^2+^ peaks were not time-locked to the post-pre pairing onset. Color bar is given in (A). (H) The number of times the astrocyte released glutamate during the 100 post-pre pairings in the t-LTD induction protocol is shown with different ΔT.

Finally, we studied in more detail how Ca^2+^ concentration behaves in the fine astrocyte process during the t-LTD induction protocol depicted in Fig 2H. The delay in the astrocytic Ca^2+^ peak responses to the post-pre pairing onset varied with the temporal difference of post-pre pairings (Fig 6A). The delay increased with the lengthening of the post-pre pairing temporal difference (Fig 6B–6D).

We then addressed the occurrence of peaks in the fine astrocyte process. Our synapse model showed that astrocytic Ca^2+^ peaks for different ΔT of the t-LTD induction protocol occurred within 2 s of each other in the beginning of the protocol (Fig 6B), whereas the peaks occurred within 10 s in the middle of the protocol (Fig 6C) and within 14 s towards the end of the protocol (Fig 6D). The normalized mean peak value of the astrocytic Ca^2+^ concentration increased with the shortening of the temporal difference between post-pre pairings, having the highest values during the first 50 post-pre pairings and the lowest during the last 50 post-pre pairings in the t-LTD induction protocol (Fig 6F). It is of interest to explore these *in silico* results further in future wet-lab experiments to make it possible to build more sophisticated and biologically relevant models for astrocytes.

Previous experimental results have shown that Ca^2+^ transients do not occur at a certain fixed time point after each individual post-pre pairing, but are rather evenly distributed in the 5 s long period between each post-pre pairing [15]. We confirmed this experimental finding by calculating the probability of astrocytic Ca^2+^ peaks occurring in the 5 s long period between each pairing with different ΔT (Fig 6G). One of the reasons behind this distribution is that astrocytes are activated slower than the individual post-pre pairings because of the slow endocannabinoid signaling [15]. Our synapse model predicted that the astrocytic Ca^2+^ concentration oscillated every 13 s during the t-LTD induction protocol (13.34 s for ΔT = −10 ms and 13.67 s for ΔT = −200 ms), which is close to the reported experimental oscillation rate of every 15 s for ΔT = −25 ms [15]. Note that both the experimental and computational values are about 3 times longer than the length between each post-pre pairings. The number of times the astrocyte released glutamate during the whole 100 post-pre pairings in the t-LTD induction protocol increased with the shortening of ΔT (Fig 6E and 6H). In our synapse model, this is due to the fact that astrocytic Ca^2+^ peaks were slightly higher with shorter ΔT. There is experimental evidence showing that Ca^2+^ peaks are not higher with shorter ΔT but instead Ca^2+^ transients are more frequent and an individual Ca^2+^ transient lasts longer [15]. Our astrocyte model is based on the same mechanisms as the models published so far [78, 79]. This issue with more frequent and longer Ca^2+^ transients clearly requires further experimental clarification, so that future computational models may be extended to incorporate more realistic Ca^2+^ transients using available simulation tools, for example [92].

In summary, the model simulations confirmed several experimentally obtained results, such as t-LTD sensitivity to ΔT [12, 15] and the role of astrocytic signaling in t-LTD [15]. Moreover, the model simulations predicted the time courses of astrocytic Ca^2+^ signals and the putative roles and time courses of presynaptic mechanisms in t-LTD. These predictions will be useful in planning the future studies of astrocytes and synapses in somatosensory cortex *in vivo*.

## Discussion

Astrocytes have been shown to dynamically modulate synaptic transmission and plasticity in some cortical synapses, but how this occurs in time and space has remained unclear [15, 17, 27, 93]. We demonstrate with a new somatosensory cortical synapse model that a well-established feedback signal from a postsynaptic neuron to a presynaptic neuron via a fine astrocyte process can induce, maintain, and modulate spike-timing-dependency of long-term depression during postnatal development at cortical layer 4 to layer 2/3 synapses. This modulation occurs through astrocyte-mediated molecular mechanisms to the presynaptic axonal terminal. We predict for the first time the dynamics of these molecular mechanisms underlying spike-timing-dependent LTD and link complex biochemical networks at the pre- and postsynaptic as well as astrocytic sites to the electrophysiology and time window of spike-timing-dependent plasticity induction at vertical L4-L2/3 synapses [12, 15]. The removal of any of the key mechanisms, including the astrocytic mechanisms, impaired synaptic t-LTD. Our results indicate that multiple biophysical and biochemical plasticity mechanisms at the L4-L2/3 neuronal synapse and nearby fine astrocyte process contribute to enabling synaptic LTD in a developing somatosensory cortex.

Our study highlights several important advancements in neuroscience. First, we link together the dynamics of known cellular and molecular players of t-LTD during postnatal development and describe each model component by mathematical equations and data from a multitude of experimental and modeling studies. Second, we combine unique experimental results on the time-dependency of t-LTD in a developing somatosensory cortex, obtained by two independent research groups [11, 12, 14, 15], to validate our model. Third, our analysis using the biophysically and biochemically detailed synapse model confirms the experimental findings on astrocytes’ ability in setting the temporal difference of t-LTD at L4-L2/3 synapses [15]. In summary, we confirm with our *in silico* synapse model the following experimental findings and predictions (1–4).

1. The fine sensitivity of t-LTD to the temporal difference in a developing somatosensory cortex is achieved through complex molecular signaling, similarly to experimental data and predictions [11, 12, 15].
2. At the L4 spiny stellate cell—L2/3 pyramidal cell synapse, t-LTD is orchestrated through the postsynaptic release of endocannabinoid molecules (agonist 2-AG) detected by CB_1_Rs on the fine astrocyte process [15].
3. Astrocytic Ca^2+^ transients induced by endocannabinoids and subsequent exocytosis of glutamate from the fine astrocyte process are appropriate to induce and maintain t-LTD (comparable to experimental validation data [15]).
4. Glutamate release from the fine astrocyte process can be detected by presynaptic NMDARs at the time courses appropriate for the modulation of synaptic release through calcineurin-related signaling [14].

We modeled all the above-mentioned mechanisms using biologically realistic time constants validated against published experimental data (see Materials and methods and S1 Appendix). The predictions made by our synapse model are readily available for further experimental wet-lab testing. In addition, we provide all mathematical equations and their relationships, all parameter values, all references used in the model construction, and commented code upon publication in order to enable the reproduction of our results and facilitate reproducible science [76–79, 94].

In a developing somatosensory cortex, t-LTD has been shown to require activation of CB_1_Rs by postsynaptically released endocannabinoids, and increased astrocytic Ca^2+^ concentration [15]. However, the spatial location and distribution of the CB_1_Rs is under debate. Earlier it was assumed that t-LTD requires CB_1_Rs located on the presynaptic neuron [95], but more recent evidence from several brain areas and spinal cord shows that CB_1_Rs are also located on astrocytes [15, 96–98]. Agonists of CB_1_Rs have been found to evoke Ca^2+^ transients in astrocytes [97] and in the micro-domains of astrocyte processes [15, 98]. Furthermore, it has been shown that a prerequisite for t-LTD in the somatosensory cortex [15], and also in the hippocampus [96], are astrocytic CB_1_Rs, not the presynaptic CB_1_Rs. Based on these most recent findings we modeled the postsynaptically released endocannabinoid activation only on the astrocytic CB_1_Rs [15]. In addition, we made an assumption that an increase in astrocytic Ca^2+^ levels, due to endocannabinoids in our model, is mediated by IP_3_Rs on the ER membrane [40] and subsequent Ca^2+^-dependent glutamate exocytosis [42, 99, 100]. Recently, studies have found multiple types of Ca^2+^ signals in astrocyte processes [44], also in somatosensory cortex *in vivo* [15, 101, 102]. These multiple types of Ca^2+^ signals may be explained by the activation of different subtypes 1, 2, and 3 of IP_3_Rs [40]. Although evidence against [103, 104] and for [47] IP_3_R-mediated Ca^2+^-dependent glutamate exocytosis in plasticity exist, we decided to test with our model whether the kinetics of Ca^2+^-dependent glutamate exocytosis in astrocyte processes can take part in mediating t-LTD observed in somatosensory cortex during postnatal development. The controversial results between different studies may be explained by various factors, including differences in the postnatal developmental stage of the rodent, the brain area, the type of a synapse and brain circuitry, the motility of astrocyte processes, and the experimental conditions as well as the different measurement techniques, including the use of transgenic animals. Furthermore, other Ca^2+^-related mechanisms may coexist in astrocyte processes [40, 42–49] which can also contribute to the modulation of plasticity.

In addition to astrocytic CB_1_Rs, the activation of presynaptic NMDARs is required for t-LTD in the developing somatosensory cortex [9–14]. These presynaptic NMDARs have been shown to be tightly linked with presynaptic Ca^2+^, proteins and associated signaling cascades to control the release of neurotransmitters from the vesicles, the size of the vesicle pool, and/or the replenishment of synaptic vesicle pools [14]. The exact signaling between astrocytic CB_1_Rs and presynaptic NMDARs cooperatively leading to synaptic depression is, however, not fully understood. Moreover, the presynaptic NMDAR-dependent LTD (in the vertical pathway) seems to be developmentally regulated and disappears by 3–4 weeks of age in the mouse barrel cortex [10] and visual cortex [105] as well as in the mouse hippocampus [41]. Regardless of the few missing components and debates on the exocytosis of astrocytic glutamate and the presynaptic NMDARs [14, 17, 29, 106–108], we conclude that there is a growing body of evidence suggesting the involvement of astrocytes in t-LTD during postnatal development. Based on recent reconstruction studies of astrocytic morphologies [32] and imaging of IP_3_R-mediated events in fine astrocyte processes [40], astrocytes indeed seem to make an important contribution to synapses. Using computational modeling we present the links between different molecular pathways contributing to the temporal difference of t-LTD and the required time courses of the molecular players. The full synapse model couples the following key signaling cascades: (1) the signaling cascade from the postsynaptic terminal, thus the release of endocannabinoids, to the astrocyte, (2) the signaling cascade from the fine astrocyte process, including Ca^2+^-dependent glutamate exocytosis, to the presynaptic terminal, and (3) the signaling cascade from the presynaptic NMDARs and calcineurin (a protein phosphatase) to the vesicular release of synaptic glutamate. Based on our simulation results endocannabinoid-induced, Ca^2+^-mediated glutamate release from fine astrocyte processes *in vivo* can thus have a pivotal impact on synaptic properties and thereby on neuronal activity, most profoundly in the developing somatosensory system.

There is plenty of experimental evidence that NMDAR-dependent synaptic plasticity can be induced by several different mechanisms [14]. Studies with neocortical and hippocampal synapses show that presynaptic NMDARs typically induce LTD and postsynaptic NMDARs LTP. This indicates that presynaptic NMDARs control synaptic release and plasticity, particularly in glutamatergic synapses. The expression of presynaptic NMDARs is, however, highly heterogeneous and synapse specific [109]. For example, it has been shown that presynaptic NMDARs can selectively modulate L4-L2/3 synapses in the somatosensory cortex, but not L4-L4 or L2/3-L2/3 synapses [109]. Moreover, presynaptic NMDARs have been shown to operate in unconventional ways in some synapses [110]. At the L4-L2/3 synapse, NMDARs may therefore support a special form of plasticity, also confirmed by our modeling. Taken together, these previous results on the heterogenous expression of presynaptic NMDARs may explain the lack of presynaptic NMDAR-mediated plasticity in some studies. Furthermore, our results suggest that the astrocytic modulation of NMDAR-dependent t-LTD is highly synapse specific, and synapses that do not contain any presynaptic NMDARs cannot implement astrocytic modulation of t-LTD during postnatal development. We are not aware of any study showing that astrocytes are not modulating t-LTD at L4-L2/3 synapses. All these findings highlight the acute need for detailed mechanistic modeling such as our present study where we show astrocytic CB_1_R- and presynaptic NMDAR-dependent t-LTD in a developing somatosensory cortex. It is also likely that some additional plasticity mechanisms could be added to the model or that their role could be fulfilled by multiple redundant parallel plasticity pathways.

For more than ten years, STDP has been suggested to underlie the development of sensory representations and synapse maturation in the somatosensory cortex [111]. In particular, t-LTD at L4-L2/3 synapses in rodents has been shown to be vital for plasticity during postnatal development [89]. A growing body of evidence also suggests that astrocytes have a fundamental role in cortical postnatal development and map plasticity [15, 51, 112]. It has been suggested that the functional role of astrocytes in t-LTD at developing somatosensory L4-L2/3 synapses might be to act as a time buffer (or, delay factor) for neuronal activity and sensory processing that occurs on a fast millisecond timescale [15]. During these events, fast and correlated neuronal activity is integrated into slower astrocytic Ca^2+^ dynamics. It can therefore be speculated that astrocytes monitor, integrate, and modulate the activity of synapses, on longer timescales, to enhance the capacity of information processing in the brain to build a complex cognitive, conscious experience of the acquired sensory information in higher animals and humans. We have here demonstrated how this monitoring, integration, and modulation of activity is orchestrated through biophysicochemical processes in a synapse to induce t-LTD. We conclude that modeling the dynamics of neuron-astrocyte signaling in a synapse can offer profound mechanistic insights into the development of synaptic computation and information processing in sensory systems.

Developing sensory circuits undergo synapse elimination, a process of pruning synapses during development. Synapse elimination is essential for the formation of mature neuronal circuits and proper brain functions in the cerebral cortex. Although less is known about the cortical pruning compared to other areas, a disruption of this process is likely involved in neurodevelopmental diseases such as schizophrenia, autism spectrum disorder, and epilepsy [16]. The specific molecular mechanisms that drive synapse elimination remain mostly unknown. Interestingly, hippocampal astrocytes have been found to contribute to synapse elimination in a subtype 2 IP_3_R-dependent manner through the activation of purinergic signaling [113]. On the other hand, dendritic spines that have contacts with astrocytes have been found to survive longer and be morphologically more mature than those without such contacts [114]. We argue that astrocytes, potentially together with microglia, might contribute to the elimination of synapses at L4-L2/3 using t-LTD. Overall, the astrocytic modulation of STDP may be one important phase in the development of synapses and functional circuits for mature cortical sensory processing.

Different forms of plasticity, including the Hebbian type of plasticity, have been studied both in experimental and computational settings for a long time. There is accumulating evidence that Hebbian framework and plasticity rules may depend on the 3rd and 4th factors, such as neuromodulatory agents or neuroglial cells [115, 116]. The 3rd factor is usually included in phenomenological models of synaptic plasticity [115–117]. To the best of our knowledge, we present here the first computational study that provides strong supportive evidence on the role of astrocytes and their processes as a putative 3rd factor in t-LTD in the somatosensory cortex during postnatal development. Overall, our results highlight the importance of neuroglial mechanisms in STDP that may complement and stabilize developing somatosensory L4 to L2/3 synapses. The synapses in other cortical layers and brain areas as well as the inhibitory synapses deserve further study, both experimentally and computationally.

We argue that to understand how the brain functions, we need to understand both the structure and function of all the different spatial scales, from genes to the whole brain. Although a great deal of experimental work has been undertaken to study all these different scales, we still have not solved many of the puzzles the brain holds [118]. Computational modeling tightly integrated with experimental data is one of the tools that is used more and more to study brain functions on different scales [52–55, 119]. Modeling approaches bridging different organizational levels and dynamical scales have been increasingly introduced to describe complex neuronal systems [54]. We have here shown how computational modeling can provide important additional insights into the newly developed experimental tools and protocols to study astrocytes and their genetic, molecular, morphological, and physiological profiling in *in vivo* [120, 121]. With computational modeling, we can test different hypotheses, ease the planning of experimental studies, and, especially, explore the role of new mechanisms and their dynamics (temporal behavior) in different experimental settings and brain phenomena.

Our biophysically and biochemically detailed model provides several predictions that could be tested in future wet-lab experiments. The experiments should address the influence of molecular mechanisms, electrophysiological properties, and patterns of neuronal activity on the t-LTD time window (Fig 4). An additional testable key prediction of our work is the astrocytic Ca^2+^ signals, shown in Fig 6, by using the same t-LTD induction protocol with different temporal differences. The testing of these predictions requires a combination of electrophysiological, Ca^2+^ imaging, and molecular biology techniques. New experimental data could also be helpful in refining some of the model components, particularly the subcellular ones. There are new emerging techniques for single cells developed in the intersection of engineering and biology [122]. These techniques could be used to refine the description of signal transduction pathways, especially the calcineurin-related pathway in the presynaptic terminal. The concentration levels of key molecular species and the rates of molecular reactions could be measured during plasticity induction both in a single cortical neuron and cortical astrocyte using novel imaging techniques [121]. The NMDAR functioning should as well be further studied in wet-lab experiments, particularly addressing the type, time courses, and density of presynaptic NMDARs. There is a great demand for new targets for treating neurodevelopmental disorders and diseases. Systematic collection of experimental data on the role of how astrocytic signaling pathways impair synaptic plasticity in developmental brain disorders is crucial. Taken together, all these future experiments will enable deeper insights into the players of long-term plasticity in developing circuits in health and disease by providing data for construction and validation of models.

It is extremely complex to model synaptic plasticity and the underlying biochemical networks in a biologically meaningful way. Despite the challenges, we were able to bring about a combination of experimentally verified neuronal and astrocytic mechanisms and show how they lead to the emergence of spike-timing-dependent long-term plasticity. Our analysis confirms the experimental findings on astrocytes’ ability in setting the temporal difference of t-LTD at developing somatosensory L4-L2/3 synapses [15]. Furthermore, we predicted with our *in silico* synapse model (1) which are the key molecules related to t-LTD, (2) how the molecular reactions depend on the temporal difference of t-LTD, and (3) what are the time courses of molecular interactions. The synapse model can be used to design future wet-lab experiments and, ultimately, to clarify the controversies present in the field. Our study provides both neuronal and neuroglial elements to build sophisticated and biologically relevant large-scale neuron-astrocyte network models. With such models bridging different scales, we will expect to link the molecular, synaptic, cellular, and network level dynamics to cognitive phenomena and to assess the roles of astrocytes in higher brain functions, such as learning, memory, decision-making, sleep, and, ultimately, consciousness.

## Materials and methods

To study the role of astrocytes in modulation of t-LTD, we simulated an L4-L2/3 synapse in somatosensory cortex. We described major biophysical and biochemical mechanisms for the one-compartmental presynaptic L4 spiny stellate cell, two-compartmental (soma and dendrite) postsynaptic L2/3 pyramidal cell, and one-compartmental fine astrocyte process (Fig 1). We employed the following key assumptions to build our initial hypotheses about the testable cellular and subcellular mechanisms: (1) Endocannabinoid 2-AG activates astrocytic CB_1_Rs and triggers Ca^2+^ signaling in astrocytes in somatosensory cortex [15, 42, 97], (2) astrocytic Ca^2+^-dependent glutamate exocytosis, together with a spillover of glutamate from the synaptic cleft, has an effect on presynaptic glutamate release by modifying the release probabilities [36, 42, 88, 99], and (3) the link between the glutamate exocytosis from the astrocyte and the presynaptically released glutamate is the protein phosphatase calcineurin which is activated by the influx of Ca^2+^ through the presynaptic NMDARs [13, 14]. The model components are described using differential equations and validated against experimental data. We stimulated the synapse model using t-LTD stimulation protocols with a varying temporal difference between pre- and postsynaptic activity [12]. For clarity, only those differential equations that we developed or modified from previously published models are given next. A complete description of the model is given in S1 Appendix.

### Presynaptic neuron model

The differential equation for the presynaptic membrane potential can be given as [56]

$$\begin{array}{cc} C_{m,\text{pre}}\frac{dV_{\text{pre}}}{dt}= & -I_{\text{CaNHVA},\text{pre}}-I_{K,\text{pre}}-I_{\text{Na},\text{pre}}-I_{L,\text{pre}}\\ & -I_{\text{Ca},\text{NMDAR},\text{pre}}-I_{\text{Na},\text{NMDAR},\text{pre}}+I_{\text{ext},\text{pre}}\;, \end{array}$$

where *C*_m,pre_ is the presynaptic membrane capacitance per unit area, *I*_CaNHVA,pre_ is the current density via Ca_NHVA_ channels, *I*_K,pre_ is the K^+^ current density, *I*_Na,pre_ is the Na^+^ current density, *I*_L,pre_ is the leak current density, *I*_Ca,NMDAR,pre_ and *I*_Na,NMDAR,pre_ are the Ca^2+^ and Na^+^ current densities via NMDARs, and *I*_ext,pre_ is the stimulus current injected into the presynaptic neuron per unit area. The presynaptic channels are described by the Hodgkin-Huxley and Goldman-Hodgkin-Katz formalisms [56, 57] as explained in S1 Appendix. The differential equations for the gating variables of different currents are given in S1 Appendix [56, 57].

Presynaptic Ca^2+^ concentrations were elevated by Ca^2+^ influxes through presynaptic NMDARs and Ca_NHVA_ channels. The differential equations for the presynaptic Ca^2+^ concentration mediated by Ca_NHVA_ channels and by NMDARs are based on a previously published study [123]. The concentration of Ca^2+^ mediated by Ca_NHVA_ channels ([Ca^2+^]_CaNHVA,pre_) activates vesicle exocytosis and glutamate release from the presynaptic neuron. The concentration of Ca^2+^ mediated by NMDARs ([Ca^2+^]_NMDAR,pre_) activates presynaptic calcineurin [14], and the differential equation for the presynaptic calcineurin concentration ([CaN]_pre_) is given in S1 Appendix [60].

Calcineurin has been shown to regulate a specific phase of synaptic vesicle cycling, thus influencing the vesicle release [11, 124–126]. We modeled this effect via a signaling pathway linking calcineurin to vesicle release and recycling in the presynaptic terminal with the following differential equation

$$\frac{d{\left[X\right]}_{\text{ac},\text{pre}}}{dt}=p_{1,\text{pre}}\frac{{\left[\text{CaN}\right]}_{\text{pre}}^{n_{2,\text{pre}}}}{K_{A,\text{pre}}^{n_{2,\text{pre}}}+{\left[\text{CaN}\right]}_{\text{pre}}^{n_{2,\text{pre}}}}(X_{\text{total},\text{pre}}-{\left[X\right]}_{\text{ac},\text{pre}})\;,$$

where [X]_ac,pre_ is the active concentration and X_total,pre_ is the total concentration of the unspecified protein that affects the vesicle release, *p*_1,pre_ is the rate constant, *K*_A,pre_ is the calcineurin concentration producing half occupation, and *n*_2,pre_ is the Hill coefficient.

The differential equation for the fraction of releasable presynaptic vesicles (*R*_rel,pre_) was taken from previously published models [62–65], and the differential equation for the release probability of presynaptic glutamate vesicles was combined and modified from previously published equations [62–65] and is given as

$$\begin{array}{cc} \frac{dP_{\text{rel},\text{pre}}}{dt}= & -k_{f,\text{pre}}P_{\text{rel},\text{pre}}\\ & +\sum_{j}(1-f_{\text{pre}})\frac{{\left[{\text{Ca}}^{2+}\right]}_{\text{CaNHVA},\text{pre}}^{n_{1,\text{pre}}}}{K_{\text{rel},\text{pre}}^{n_{1,\text{pre}}}+{\left[{\text{Ca}}^{2+}\right]}_{\text{CaNHVA},\text{pre}}^{n_{1,\text{pre}}}}(1-P_{\text{rel},\text{pre}})\delta \left(t-t_{j}\right)\;, \end{array}$$

where the fraction (*f*_pre_), which is the active concentration ([X]_ac,pre_) divided by the total concentration (X_total,pre_) of the protein, affects the probability of presynaptic glutamate release. Through *f*_pre_, we modeled the inhibiting role of calcineurin in vesicle exocytosis and glutamate release [13, 14] in the presynaptic terminal. Parameters *k*_f,pre_, *K*_rel,pre_, and *n*_1,pre_ describe the facilitation rate constant, Ca^2+^ concentration producing half occupation used in calculation of glutamate release, and Hill coefficient, respectively. The presynaptic glutamate release occurs at the first time point *t* = *t*_*j*_ such that [Ca^2+^]_CaNHVA,pre_ ≥ *C*_thr,pre_ and less than 10 ms has passed from the previous presynaptic membrane potential crossing 0 mV from negative to positive voltages (*V*_pre_ ≥ 0, $\frac{dV_{\text{pre}}}{dt}>0$) at that time point *t*_*j*_. The *δ* function has units of $\frac{\text{1}}{\text{ms}}$.

The differential equation for the glutamate concentration in the synaptic cleft was combined and modified from previously published glutamate equations [63–65] and glutamate-activated postsynaptic equations related to mGluRs [53] and is given as

$$\begin{array}{cc} \frac{d{\left[\text{Glu}\right]}_{\text{syncleft}}}{dt}= & -k_{\text{Glu},f,\text{post}}(1-f_{\text{Glu},\text{pre}}){\left[\text{Glu}\right]}_{\text{syncleft}}\\ & -k_{\text{mGluR},f,\text{post}}(1-f_{\text{Glu},\text{pre}}){\left[\text{Glu}\right]}_{\text{syncleft}}{\left[\text{mGluR}\right]}_{\text{post}}\\ & +k_{\text{mGluR},b,\text{post}}{\left[\text{Glu}\_\text{mGluR}\right]}_{\text{post}}\\ & +\sum_{j}\frac{G_{\text{pre}}N_{\text{pre}}P_{\text{rel},\text{pre}}R_{\text{rel},\text{pre}}}{k_{\text{Glu},\text{pre}}N_{A}V_{\text{syncleft}}}\delta \left(t-t_{j}\right)\;, \end{array}$$

where *k*_Glu,f,post_, *k*_mGluR,f,post_, and *k*_mGluR,b,post_ are the rate constants for the postsynaptic mGluR glutamate uptake, and postsynaptic mGluR glutamate binding and unbinding, respectively. Parameter *f*_Glu,pre_ represents the spillover of glutamate from the synaptic cleft, and thus the amount 1 − *f*_Glu,pre_ denotes the part of glutamate in synaptic cleft that activates the postsynaptic receptors. [mGluR]_post_ and [Glu_mGluR]_post_ denote the concentrations of postsynaptic mGluRs and glutamate-mGluR complex, respectively. Parameters *G*_pre_, *N*_pre_, *k*_Glu,pre_, *N*_A_, and *V*_syncleft_ denote the number of glutamate per presynaptic vesicle, number of readily releasable presynaptic vesicles, scaling factor to convert from units M to μM, Avogadro’s constant, and volume of synaptic cleft, respectively.

We modeled presynaptic GluN2C/D-containing NMDARs [58, 59], because experimental studies have reported that presynaptic GluN2C/D-containing NMDARs are required for t-LTD at L4 to L2/3 synapses [9, 12, 15, 89–91]. On the other hand, postsynaptic GluN2B-containing NMDARs are necessary for t-LTP at L4-L2/3 and L2/3-L2/3 synapses, and postsynaptic GluN2A-containing NMDARs are required in t-LTD at L2/3-L2/3 synapses [9, 12, 15, 89–91, 127]. In our synapse model, presynaptic NMDARs are activated by the glutamate in the extrasynaptic space and the spillover of glutamate from the synaptic cleft. The differential equations for the presynaptic variables can be seen in S1 Appendix. Intermediate variables, parameter values, and initial values needed to solve the presynaptic neuron model are also given in S1 Appendix.

### Postsynaptic neuron model

The postsynaptic neuron model had two compartments, a soma and a dendrite, modified from a previously published study [66]. The differential equations for the membrane potentials of these two compartments are

$$\begin{array}{cc} C_{m,\text{post}}\frac{dV_{\text{soma},\text{post}}}{dt}= & -I_{\text{KDR},\text{soma},\text{post}}-I_{\text{Na},\text{soma},\text{post}}-I_{\text{NaP},\text{soma},\text{post}}\\ & -I_{L,\text{soma},\text{post}}+I_{\text{coupl},\text{soma},\text{post}}+I_{\text{ext},\text{post}} \end{array}$$

and

$$\begin{array}{cc} C_{m,\text{post}}\frac{dV_{\text{dend},\text{post}}}{dt}= & -I_{\text{KA},\text{dend},\text{post}}-I_{\text{CaLHVA},\text{dend},\text{post}}-I_{\text{CaLLVA},\text{dend},\text{post}}\\ & -I_{\text{Na},\text{dend},\text{post}}-I_{L,\text{dend},\text{post}}-I_{\text{AMPAR},\text{post}}\\ & -I_{\text{Ca},\text{NMDAR},\text{post}}+I_{\text{coupl},\text{dend},\text{post}}\;, \end{array}$$

where *C*_m,post_ is the membrane capacitance per unit area, *I*_KDR,soma,post_ is the somatic K_DR_ current density, *I*_Na,soma,post_ and *I*_Na,dend,post_ are the somatic and dendritic Na^+^ current densities, *I*_NaP,soma,post_ is the somatic Na_P_ current density, *I*_L,soma,post_ and *I*_L,dend,post_ are the somatic and dendritic leak current densities, *I*_coupl,soma,post_ and *I*_coupl,dend,post_ are the somatic and dendritic coupling terms, *I*_ext,post_ is the current injected into the soma per unit area, *I*_KA,dend,post_ is the dendritic K_A_ current density, *I*_CaLHVA,dend,post_ and *I*_CaLLVA,dend,post_ are the dendritic Ca_LHVA_ and Ca_LLVA_ current densities, and *I*_AMPAR,post_ and *I*_Ca,NMDAR,post_ are the synaptic current densities via AMPARs and NMDARs in the dendrite. Hodgkin-Huxley formalism was used to describe the behavior of ionic currents [54, 67–69] as explained in S1 Appendix.

The differential equation for the fraction of postsynaptic AMPARs in open state can be modified from previously published study [70] and is given as

$$\begin{array}{cc} \frac{dm_{\text{AMPAR},\text{post}}}{dt}=\; & \alpha_{\text{AMPAR},\text{post}}\left(1-f_{\text{Glu},\text{pre}}\right){\left[\text{Glu}\right]}_{\text{syncleft}}\left(1-m_{\text{AMPAR},\text{post}}\right)\\ & -\beta_{\text{AMPAR},\text{post}}m_{\text{AMPAR},\text{post}}\;, \end{array}$$

where *α*_AMPAR,post_ and *β*_AMPAR,post_ describe the rate constants of opening and closing postsynaptic AMPARs, respectively, and we can similarly write the equation for NMDARs. Other differential equations for the gating variables of different currents related to the membrane potential in the dendrite and in the soma as well as for the IP_3_R inactivation gating variable are given in S1 Appendix [54, 67–69, 72, 73].

The biochemical mechanisms related to mGluRs, the activation of the G-protein signaling cascade, and the production of endocannabinoids are included in the synapse model to study the effect of endocannabinoids on the adjacent astrocyte. The differential equations starting from the mGluR activation to the endocannabinoid 2-AG release were based on previously published models [53, 71]. Glutamate in the synaptic cleft binds to postsynaptic mGluRs and induces dissociation of the G protein α subunit bound with guanosine-5’-triphosphate (GαGTP) from the mGluR-bound G protein with β and γ subunits (Gβγ). Calcium can bind to PLC, and, in addition, GαGTP can enhance its activity. The postsynaptic Ca^2+^ equation was based on previously published models [53, 72–74]. Active PLC produces IP_3_ and DAG from phosphatidylinositol 4,5-bisphosphate (PIP_2_). After Ca^2+^ binds to DAG lipase, the complex binds to DAG and catalyzes 2-AG synthesis. The differential equations, intermediate variables, parameter values, and initial values needed to solve the postsynaptic neuron model are given in S1 Appendix.

### Astrocyte model

More and more evidence about the complexity of astrocyte processes is accumulating *in vivo*. Several studies have revealed Ca^2+^ activity [27, 44, 101, 102, 128–131] and complex molecular and biochemical mechanisms [40, 42–49] in the main and fine processes of astrocytes. Currently it is not clear how these signals and the underlying subcellular mechanisms are linked. These recent findings encouraged us to test the dynamical capacity and the role of astrocytic Ca^2+^-dependent glutamate exocytosis in the endocannabinoid-mediated signaling from the postsynaptic terminal to the presynaptic terminal to modulate synaptic functions. The fine astrocyte process in our model is assumed to contain IP_3_R-mediated Ca^2+^-dependent glutamate exocytosis [42, 99, 100], similar to earlier published models on astrocyte-neuron interactions (for a summary of models, see [78, 79]). We modeled IP_3_R-mediated Ca^2+^-dependent glutamate exocytosis as a generic glutamate exocytosis, not as a biochemically detailed vesicular release due to lacking molecular details [132, 133]. Contradictory evidence has been presented on the involvement of astrocytic IP_3_Rs on synaptic plasticity in hippocampal slices, using transgenic mice that lack the commonly expressed subtype 2 of the IP_3_Rs in astrocytes [103, 134]. It is possible that other IP_3_R subtypes exist in astrocyte processes [40], indeed knocking out the subtype 2 of IP_3_Rs has been shown to abolish all Ca^2+^ signals in astrocyte soma but only about half in the astrocyte processes [44]. These recent results about the diversity of IP_3_R subtypes in astrocyte processes [40] provide additional justification for our assumption to further examine the significance of the kinetics of IP_3_R-mediated Ca^2+^-dependent glutamate exocytosis in t-LTD in somatosensory cortex [15, 42]. Other mechanisms that couple the endocannabinoids to astrocyte Ca^2+^ signaling may coexist in somatosensory cortex in different phases of development and should be studied in the future, but our study focused on the most modeled and tested Ca^2+^-dependent mechanism in astrocytes.

We modeled Ca^2+^ and IP_3_ concentrations, and the gating variable for IP_3_R inactivation in the astrocyte based on previously published models [72, 73, 75, 80]. The differential equation for the astrocytic IP_3_ concentration was modified to be

$$\frac{d{\left[{\text{IP}}_{3}\right]}_{\text{astro}}}{dt}=\frac{{\text{IP}}_{3,\text{astro}}^{⋆}-{\left[{\text{IP}}_{3}\right]}_{\text{astro}}}{\tau_{\text{IP}3,\text{astro}}}+r_{\text{IP}3,\text{astro}}\left({\left[2-\text{AG}\right]}_{\text{post}}-{\text{AG}}_{\text{post}}^{⋆}\right)\;,$$

where ${\text{IP}}_{3,\text{astro}}^{⋆}$, *τ*_IP3,astro_, *r*_IP3,astro_, [2-AG]_post_, and ${\text{AG}}_{\text{post}}^{⋆}$ denote the resting concentration of IP_3_, time constant for IP_3_ degradation, rate constant of IP_3_ production, concentration of the endocannabinoid 2-AG released from the postsynaptic neuron, and resting concentration of 2-AG, respectively. The differential equation for the fraction of releasable glutamate resources in the astrocyte was taken from previously published models [62–65] and the glutamate concentration in the extrasynaptic space was also taken from the previously published model [64, 65]. The differential equations, intermediate variables, parameter values, and initial values needed to solve the astrocyte model are given in S1 Appendix.

### Stimulation protocols

The following protocols were used [12]:

1. The stimulation protocol before t-LTD induction consisted of five 10 ms long presynaptic stimuli at a frequency of 0.2 Hz and with an amplitude of 10 $\frac{\mu \text{A}}{{\text{cm}}^{2}}$ keeping the fraction of presynaptic glutamate release inhibition (*f*_pre_) as constant zero (Fig 2A).
2. The t-LTD induction protocol consisted of a 10 ms long postsynaptic stimulus with an amplitude of 25 $\frac{\mu \text{A}}{{\text{cm}}^{2}}$ occurring between 10 ms and 200 ms before a 10 ms long presynaptic stimulus with an amplitude of 10 $\frac{\mu \text{A}}{{\text{cm}}^{2}}$ and the post-pre pairing was repeated 100 times at a frequency of 0.2 Hz (Fig 2H). Thus, the temporal difference (ΔT) between the pre- and postsynaptic stimulus in this study had negative values meaning that the postsynaptic stimulus occurred before the presynaptic stimulus (ΔT had values between −10 ms and −200 ms). The initial value of *f*_pre_ in these simulations was zero, but it increased during the stimulation protocol to above zero depending on ΔT used in the protocol.
3. The stimulation protocol after t-LTD induction consisted of five 10 ms long presynaptic stimuli at a frequency of 0.2 Hz and with an amplitude of 10 $\frac{\mu \text{A}}{{\text{cm}}^{2}}$ (Fig 2O) keeping *f*_pre_ as constant value that is the final simulation value obtained from the simulation with the t-LTD induction protocol. Thus, the fraction *f*_pre_ had different constant values depending on ΔT used in the t-LTD induction protocol (Fig 2H).

### Data analysis

Data was analyzed in MATLAB^®^. We calculated the amplitude of the postsynaptic EPSP in the soma during the first presynaptic stimulus in the stimulation protocol before t-LTD induction and also for every ΔT in the stimulation protocol after t-LTD induction. To obtain the EPSP percentage, we normalized for every ΔT the EPSP amplitude occurring after t-LTD induction by the EPSP amplitude occurring before t-LTD induction, and multiplied them by 100%. Furthermore, we calculated the ΔEPSP percentage for every ΔT as the percentage change between the somatic EPSP amplitude occurring before t-LTD induction and the somatic EPSP amplitude occurring after t-LTD induction. Thus, the ΔEPSP percentage was obtained by subtracting the somatic EPSP amplitude occurring before t-LTD induction from the somatic EPSP amplitude occurring after t-LTD induction, dividing this change with the somatic EPSP amplitude occurring before t-LTD induction, and finally multiplying by 100%. The shorter way to calculate the ΔEPSP percentage was to subtract 100% from the EPSP percentage. The experimental ΔEPSP data for comparison was calculated from the normalized EPSP slopes and obtained from the literature [12].

We calculated the Ca^2+^ peak values, peak times, and oscillation frequencies during the t-LTD induction protocol for every ΔT. We divided the 500 s long post-pre pairing simulation data into 5 s long sweeps, where each sweep represents one post-pre pairing (0.2 Hz stimulus). We reorganized the peak times of Ca^2+^ oscillations into the 5 s long sweeps. Then we reorganized the number of peaks occurring in ten 0.5 s long bins. We obtained the probability of astrocytic Ca^2+^ peaks by normalizing the reorganized data by the total number of peaks [15].

### Simulation details and code

Simulation code was written in Python 3.7. The code is available in the ModelDB [135] (http://modeldb.yale.edu/266819) and in the author’s GitHub page (https://github.com/TiinaManninen/synapsemodel). State variables were updated using the forward Euler method with 0.05 ms step size.

## Supporting information

S1 Appendix Full description of the synapse model. (PDF)

## References

[1] Markram H, Gerstner W, Sjöström PJ. A history of spike-timing-dependent plasticity. Front Synaptic Neurosci. 2011;3:4 10.3389/fnsyn.2011.00004 22007168 PMC3187646

[2] Markram H, Lübke J, Frotscher M, Sakmann B. Regulation of synaptic efficacy by coincidence of postsynaptic APs and EPSPs. Science. 1997;275(5297):213–215. 10.1126/science.275.5297.213 8985014

[3] Bi GQ, Poo MM. Synaptic modifications in cultured hippocampal neurons: dependence on spike timing, synaptic strength, and postsynaptic cell type. J Neurosci. 1998;18(24):10464–10472. 10.1523/JNEUROSCI.18-24-10464.1998 9852584 PMC6793365

[4] Citri A, Malenka RC. Synaptic plasticity: multiple forms, functions, and mechanisms. Neuropsychopharmacology. 2008;33(1):18–41. 10.1038/sj.npp.1301559 17728696

[5] Manninen T, Hituri K, Hellgren Kotaleski J, Blackwell KT, Linne ML. Postsynaptic signal transduction models for long-term potentiation and depression. Front Comput Neurosci. 2010;4:152 10.3389/fncom.2010.00152 21188161 PMC3006457

[6] Hellgren Kotaleski J, Blackwell KT. Modelling the molecular mechanisms of synaptic plasticity using systems biology approaches. Nat Rev Neurosci. 2010;11(4):239–251. 10.1038/nrn2807 20300102 PMC4831053

[7] Nicoll RA. A brief history of long-term potentiation. Neuron. 2017;93(2):281–290. 10.1016/j.neuron.2016.12.015 28103477

[8] Feldman DE. Timing-based LTP and LTD at vertical inputs to layer II/III pyramidal cells in rat barrel cortex. Neuron. 2000;27(1):45–56. 10.1016/S0896-6273(00)00008-8 10939330

[9] Rodríguez-Moreno A, Paulsen O. Spike timing–dependent long-term depression requires presynaptic NMDA receptors. Nat Neurosci. 2008;11(7):744–745. 10.1038/nn.2125 18516036

[10] Banerjee A, Meredith RM, Rodríguez-Moreno A, Mierau SB, Auberson YP, Paulsen O. Double dissociation of spike timing–dependent potentiation and depression by subunit-preferring NMDA receptor antagonists in mouse barrel cortex. Cereb Cortex. 2009;19(12):2959–2969. 10.1093/cercor/bhp067 19363149 PMC2774397

[11] Rodríguez-Moreno A, González-Rueda A, Banerjee A, Upton AL, Craig MT, Paulsen O. Presynaptic self-depression at developing neocortical synapses. Neuron. 2013;77(1):35–42. 10.1016/j.neuron.2012.10.035 23312514 PMC3542421

[12] Banerjee A, González-Rueda A, Sampaio-Baptista C, Paulsen O, Rodríguez-Moreno A. Distinct mechanisms of spike timing-dependent LTD at vertical and horizontal inputs onto L2/3 pyramidal neurons in mouse barrel cortex. Physiol Rep. 2014;2(3):e00271 10.1002/phy2.271 24760524 PMC4002250

[13] Banerjee A, Larsen RS, Philpot BD, Paulsen O. Roles of presynaptic NMDA receptors in neurotransmission and plasticity. Trends Neurosci. 2016;39(1):26–39. 10.1016/j.tins.2015.11.001 26726120 PMC4716805

[14] Bouvier G, Larsen RS, Rodriguez-Moreno A, Paulsen O, Sjöström PJ. Towards resolving the presynaptic NMDA receptor debate. Curr Opin Neurobiol. 2018;51:1–7. 10.1016/j.conb.2017.12.020 29454833

[15] Min R, Nevian T. Astrocyte signaling controls spike timing–dependent depression at neocortical synapses. Nat Neurosci. 2012;15(5):746–753. 10.1038/nn.3075 22446881

[16] Neniskyte U, Gross CT. Errant gardeners: glial-cell-dependent synaptic pruning and neurodevelopmental disorders. Nat Rev Neurosci. 2017;18(11):658–670. 10.1038/nrn.2017.110 28931944

[17] Volterra A, Liaudet N, Savtchouk I. Astrocyte Ca^2+^ signalling: an unexpected complexity. Nat Rev Neurosci. 2014;15(5):327–335. 10.1038/nrn3725 24739787

[18] Magistretti PJ, Allaman I. Lactate in the brain: from metabolic end-product to signalling molecule. Nat Rev Neurosci. 2018;19(4):235–249. 10.1038/nrn.2018.19 29515192

[19] Allen NJ, Eroglu C. Cell biology of astrocyte-synapse interactions. Neuron. 2017;96(3):697–708. 10.1016/j.neuron.2017.09.056 29096081 PMC5687890

[20] Araque A, Parpura V, Sanzgiri RP, Haydon PG. Tripartite synapses: glia, the unacknowledged partner. Trends Neurosci. 1999;22(5):208–215. 10.1016/S0166-2236(98)01349-6 10322493

[21] Danbolt NC. Glutamate uptake. Prog Neurobiol. 2001;65(1):1–105. 10.1016/S0301-0082(00)00067-8 11369436

[22] Orkand RK, Nicholls JG, Kuffler SW. Effect of nerve impulses on the membrane potential of glial cells in the central nervous system of amphibia. J Neurophysiol. 1966;29(4):788–806. 10.1152/jn.1966.29.4.788 5966435

[23] Pannasch U, Rouach N. Emerging role for astroglial networks in information processing: from synapse to behavior. Trends Neurosci. 2013;36(7):405–417. 10.1016/j.tins.2013.04.004 23659852

[24] Oliveira JF, Sardinha VM, Guerra-Gomes S, Araque A, Sousa N. Do stars govern our actions? Astrocyte involvement in rodent behavior. Trends Neurosci. 2015;38(9):535–549. 10.1016/j.tins.2015.07.006 26316036

[25] Poskanzer KE, Yuste R. Astrocytes regulate cortical state switching in vivo. Proc Natl Acad Sci USA. 2016;113(19):E2675–E2684. 10.1073/pnas.1520759113 27122314 PMC4868485

[26] Chever O, Dossi E, Pannasch U, Derangeon M, Rouach N. Astroglial networks promote neuronal coordination. Sci Signal. 2016;9(410):ra6 10.1126/scisignal.aad3066 26758214

[27] Lines J, Martin ED, Kofuji P, Aguilar J, Araque A. Astrocytes modulate sensory-evoked neuronal network activity. Nat Commun. 2020;11:3689 10.1038/s41467-020-17536-3 32704144 PMC7378834

[28] Foley J, Blutstein T, Lee S, Erneux C, Halassa MM, Haydon P. Astrocytic IP_3_/Ca^2+^ signaling modulates theta rhythm and REM sleep. Front Neural Circuits. 2017;11:3 10.3389/fncir.2017.00003 28167901 PMC5253379

[29] Bazargani N, Attwell D. Astrocyte calcium signaling: the third wave. Nat Neurosci. 2016;19(2):182–189. 10.1038/nn.4201 26814587

[30] Oberheim NA, Takano T, Han X, He W, Lin JHC, Wang F, et al Uniquely hominid features of adult human astrocytes. J Neurosci. 2009;29(10):3276–3287. 10.1523/JNEUROSCI.4707-08.2009 19279265 PMC2819812

[31] Mederos S, González-Arias C, Perea G. Astrocyte–neuron networks: A multilane highway of signaling for homeostatic brain function. Front Synaptic Neurosci. 2018;10:45 10.3389/fnsyn.2018.00045 30542276 PMC6277918

[32] Calì C, Agus M, Kare K, Boges DJ, Lehväslaiho H, Hadwiger M, et al 3D cellular reconstruction of cortical glia and parenchymal morphometric analysis from Serial Block-Face Electron Microscopy of juvenile rat. Prog Neurobiol. 2019;183:101696 10.1016/j.pneurobio.2019.101696 31550514

[33] Sibille J, Pannasch U, Rouach N. Astroglial potassium clearance contributes to short-term plasticity of synaptically evoked currents at the tripartite synapse. J Physiol. 2014;592(1):87–102. 10.1113/jphysiol.2013.261735 24081156 PMC3903353

[34] Petrelli F, Dallérac G, Pucci L, Calì C, Zehnder T, Sultan S, et al Dysfunction of homeostatic control of dopamine by astrocytes in the developing prefrontal cortex leads to cognitive impairments. Mol Psychiatry. 2018; p. 1–18. 10.1038/s41380-018-0226-y 30127471 PMC7156348

[35] Yang Y, Ge W, Chen Y, Zhang Z, Shen W, Wu C, et al Contribution of astrocytes to hippocampal long-term potentiation through release of D-serine. Proc Natl Acad Sci USA. 2003;100(25):15194–15199. 10.1073/pnas.2431073100 14638938 PMC299953

[36] Perea G, Araque A. Astrocytes potentiate transmitter release at single hippocampal synapses. Science. 2007;317(5841):1083–1086. 10.1126/science.1144640 17717185

[37] Takata N, Mishima T, Hisatsune C, Nagai T, Ebisui E, Mikoshiba K, et al Astrocyte calcium signaling transforms cholinergic modulation to cortical plasticity *in vivo*. J Neurosci. 2011;31(49):18155–18165. 10.1523/JNEUROSCI.5289-11.2011 22159127 PMC6634158

[38] Navarrete M, Perea G, de Sevilla DF, Gómez-Gonzalo M, Núñez A, Martín ED, et al Astrocytes mediate in vivo cholinergic-induced synaptic plasticity. PLoS Biol. 2012;10(2):e1001259 10.1371/journal.pbio.1001259 22347811 PMC3279365

[39] Letellier M, Park YK, Chater TE, Chipman PH, Gautam SG, Oshima-Takago T, et al Astrocytes regulate heterogeneity of presynaptic strengths in hippocampal networks. Proc Natl Acad Sci USA. 2016;113(19):E2685–E2694. 10.1073/pnas.1523717113 27118849 PMC4868440

[40] Sherwood MW, Arizono M, Hisatsune C, Bannai H, Ebisui E, Sherwood JL, et al Astrocytic IP_3_Rs: Contribution to Ca^2+^ signalling and hippocampal LTP. Glia. 2017;65(3):502–513. 10.1002/glia.23107 28063222

[41] Falcón-Moya R, Pérez-Rodríguez M, Prius-Mengual J, Andrade-Talavera Y, Arroyo-García LE, Pérez-Artés R, et al Astrocyte-mediated switch in spike timing-dependent plasticity during hippocampal development. Nat Commun. 2020;11(1):4388 10.1038/s41467-020-18024-4 32873805 PMC7463247

[42] Rasooli-Nejad S, Palygin O, Lalo U, Pankratov Y. Cannabinoid receptors contribute to astroglial Ca^2+^-signalling and control of synaptic plasticity in the neocortex. Phil Trans R Soc B. 2014;369(1654):20140077 10.1098/rstb.2014.0077 25225106 PMC4173298

[43] Bernardinelli Y, Randall J, Janett E, Nikonenko I, König S, Jones EV, et al Activity-dependent structural plasticity of perisynaptic astrocytic domains promotes excitatory synapse stability. Curr Biol. 2014;24(15):1679–1688. 10.1016/j.cub.2014.06.025 25042585

[44] Srinivasan R, Huang BS, Venugopal S, Johnston AD, Chai H, Zeng H, et al Ca^2+^ signaling in astrocytes from Ip3r2^-/-^ mice in brain slices and during startle responses *in vivo*. Nat Neurosci. 2015;18(5):708–717. 10.1038/nn.4001 25894291 PMC4429056

[45] Boulay AC, Saubaméa B, Adam N, Chasseigneaux S, Mazaré N, Gilbert A, et al Translation in astrocyte distal processes sets molecular heterogeneity at the gliovascular interface. Cell discovery. 2017;3:17005 10.1038/celldisc.2017.5 28377822 PMC5368712

[46] Sakers K, Lake AM, Khazanchi R, Ouwenga R, Vasek MJ, Dani A, et al Astrocytes locally translate transcripts in their peripheral processes. Proc Natl Acad Sci USA. 2017;114(19):E3830–E3838. 10.1073/pnas.1617782114 28439016 PMC5441704

[47] Navarrete M, Cuartero MI, Palenzuela R, Draffin JE, Konomi A, Serra I, et al Astrocytic p38αMAPK drives NMDA receptor-dependent long-term depression and modulates long-term memory. Nat Commun. 2019;10(1):2968 10.1038/s41467-019-10830-9 31273206 PMC6609681

[48] Venturini A, Passalacqua M, Pelassa S, Pastorino F, Tedesco M, Cortese K, et al Exosomes from astrocyte processes: signaling to neurons. Front Pharmacol. 2019;10:1452 10.3389/fphar.2019.01452 31849688 PMC6901013

[49] Mazaré N, Oudart M, Moulard J, Cheung G, Tortuyaux R, Mailly P, et al Local translation in perisynaptic astrocytic processes is specific and changes after fear conditioning. Cell Rep. 2020;32(8):108076 10.1016/j.celrep.2020.108076 32846133 PMC7450274

[50] Bernardinelli Y, Muller D, Nikonenko I. Astrocyte-synapse structural plasticity. Neural Plast. 2014;2014:232105 10.1155/2014/232105 24511394 PMC3910461

[51] Sims RE, Butcher JB, Parri HR, Glazewski S. Astrocyte and neuronal plasticity in the somatosensory system. Neural Plast. 2015;2015:732014 10.1155/2015/732014 26345481 PMC4539490

[52] Bhalla US, Iyengar R. Emergent properties of networks of biological signaling pathways. Science. 1999;283(5400):381–387. 10.1126/science.283.5400.381 9888852

[53] Kim B, Hawes SL, Gillani F, Wallace LJ, Blackwell KT. Signaling pathways involved in striatal synaptic plasticity are sensitive to temporal pattern and exhibit spatial specificity. PLoS Comput Biol. 2013;9(3):e1002953 10.1371/journal.pcbi.1002953 23516346 PMC3597530

[54] Markram H, Muller E, Ramaswamy S, Reimann MW, Abdellah M, Sanchez CA, et al Reconstruction and simulation of neocortical microcircuitry. Cell. 2015;163(2):456–492. 10.1016/j.cell.2015.09.029 26451489

[55] Gallimore AR, Kim T, Tanaka-Yamamoto K, De Schutter E. Switching on depression and potentiation in the cerebellum. Cell Rep. 2018;22(3):722–733. 10.1016/j.celrep.2017.12.084 29346769

[56] Lavzin M, Rapoport S, Polsky A, Garion L, Schiller J. Nonlinear dendritic processing determines angular tuning of barrel cortex neurons *in vivo*. Nature. 2012;490(7420):397–401. 10.1038/nature11451 22940864

[57] Safiulina VF, Caiati MD, Sivakumaran S, Bisson G, Migliore M, Cherubini E. Control of GABA release at single mossy fiber-CA3 connections in the developing hippocampus. Front Synaptic Neurosci. 2010;2:1 10.3389/neuro.19.001.2010 21423487 PMC3059712

[58] Erreger K, Dravid SM, Banke TG, Wyllie DJA, Traynelis SF. Subunit-specific gating controls rat NR1/NR2A and NR1/NR2B NMDA channel kinetics and synaptic signalling profiles. J Physiol. 2005;563(2):345–358. 10.1113/jphysiol.2004.080028 15649985 PMC1665591

[59] Clarke RJ, Johnson JW. Voltage-dependent gating of NR1/2B NMDA receptors. J Physiol. 2008;586(23):5727–5741. 10.1113/jphysiol.2008.160622 18936081 PMC2655412

[60] Fiala JC, Grossberg S, Bullock D. Metabotropic glutamate receptor activation in cerebellar Purkinje cells as substrate for adaptive timing of the classically conditioned eye-blink response. J Neurosci. 1996;16(11):3760–3774. 10.1523/JNEUROSCI.16-11-03760.1996 8642419 PMC6578825

[61] Tsodyks MV, Markram H. The neural code between neocortical pyramidal neurons depends on neurotransmitter release probability. Proc Natl Acad Sci USA. 1997;94(2):719–723. 10.1073/pnas.94.2.719 9012851 PMC19580

[62] Tsodyks M, Pawelzik K, Markram H. Neural networks with dynamic synapses. Neural Comput. 1998;10(4):821–835. 10.1162/089976698300017502 9573407

[63] Lee CCJ, Anton M, Poon CS, McRae GJ. A kinetic model unifying presynaptic short-term facilitation and depression. J Comput Neurosci. 2009;26(3):459–473. 10.1007/s10827-008-0122-6 19093195 PMC2766601

[64] De Pittà M, Volman V, Berry H, Ben-Jacob E. A tale of two stories: astrocyte regulation of synaptic depression and facilitation. PLoS Comput Biol. 2011;7(12):e1002293 10.1371/journal.pcbi.1002293 22162957 PMC3228793

[65] De Pittà M, Brunel N. Modulation of synaptic plasticity by glutamatergic gliotransmission: a modeling study. Neural Plast. 2016;2016:7607924 10.1155/2016/7607924 27195153 PMC4852535

[66] Pinsky PF, Rinzel J. Intrinsic and network rhythmogenesis in a reduced Traub model for CA3 neurons. J Comput Neurosci. 1994;1(1):39–60. 10.1007/BF00962717 8792224

[67] Sarid L, Bruno R, Sakmann B, Segev I, Feldmeyer D. Modeling a layer 4-to-layer 2/3 module of a single column in rat neocortex: interweaving *in vitro* and *in vivo* experimental observations. Proc Natl Acad Sci USA. 2007;104(41):16353–16358. 10.1073/pnas.0707853104 17913876 PMC2000451

[68] Reuveni I, Friedman A, Amitai Y, Gutnick MJ. Stepwise repolarization from Ca^2+^ plateaus in neocortical pyramidal cells: evidence for nonhomogeneous distribution of HVA Ca^2+^ channels in dendrites. J Neurosci. 1993;13(11):4609–4621. 10.1523/JNEUROSCI.13-11-04609.1993 8229187 PMC6576337

[69] Avery RB, Johnston D. Multiple channel types contribute to the low-voltage-activated calcium current in hippocampal CA3 pyramidal neurons. J Neurosci. 1996;16(18):5567–5582. 10.1523/JNEUROSCI.16-18-05567.1996 8795613 PMC6578965

[70] Destexhe A, Mainen ZF, Sejnowski TJ. Kinetic models of synaptic transmission In: Koch C, Segev I, editors. Methods in neuronal modeling. Cambridge, MA: MIT Press; 1998 p. 1–25.

[71] Zachariou M, Alexander SPH, Coombes S, Christodoulou C. A biophysical model of endocannabinoid-mediated short term depression in hippocampal inhibition. PLoS ONE. 2013;8(3):e58926 10.1371/journal.pone.0058926 23527052 PMC3601106

[72] De Young GW, Keizer J. A single-pool inositol 1,4,5-trisphosphate-receptor-based model for agonist-stimulated oscillations in Ca^2+^ concentration. Proc Natl Acad Sci USA. 1992;89(20):9895–9899. 10.1073/pnas.89.20.9895 1329108 PMC50240

[73] Li YX, Rinzel J. Equations for InsP_3_ receptor-mediated [Ca^2+^]_i_ oscillations derived from a detailed kinetic model: a Hodgkin-Huxley like formalism. J Theor Biol. 1994;166(4):461–473. 10.1006/jtbi.1994.1041 8176949

[74] Blackwell KT. Calcium waves and closure of potassium channels in response to GABA stimulation in Hermissenda type B photoreceptors. J Neurophysiol. 2002;87(2):776–792. 10.1152/jn.00867.2000 11826046

[75] Nadkarni S, Jung P. Spontaneous oscillations of dressed neurons: a new mechanism for epilepsy? Phys Rev Lett. 2003;91(26):268101 10.1103/PhysRevLett.91.268101 14754091

[76] Manninen T, Havela R, Linne ML. Reproducibility and comparability of computational models for astrocyte calcium excitability. Front Neuroinform. 2017;11:11 10.3389/fninf.2017.00011 28270761 PMC5318440

[77] Manninen T, Aćimović J, Havela R, Teppola H, Linne ML. Challenges in reproducibility, replicability, and comparability of computational models and tools for neuronal and glial networks, cells, and subcellular structures. Front Neuroinform. 2018;12:20 10.3389/fninf.2018.00020 29765315 PMC5938413

[78] Manninen T, Havela R, Linne ML. Computational models for calcium-mediated astrocyte functions. Front Comput Neurosci. 2018;12:14 10.3389/fncom.2018.00014 29670517 PMC5893839

[79] Manninen T, Havela R, Linne ML. Computational models of astrocytes and astrocyte-neuron interactions: characterization, reproducibility, and future perspectives In: De Pittà M, Berry H, editors. Computational Glioscience. Cham, Switzerland: Springer; 2019 p. 423–454.

[80] Wade J, McDaid L, Harkin J, Crunelli V, Kelso S. Self-repair in a bidirectionally coupled astrocyte-neuron (AN) system based on retrograde signaling. Front Comput Neurosci. 2012;6:76 10.3389/fncom.2012.00076 23055965 PMC3458420

[81] Schneggenburger R, Neher E. Intracellular calcium dependence of transmitter release rates at a fast central synapse. Nature. 2000;406(6798):889–893. 10.1038/35022702 10972290

[82] Lou X, Scheuss V, Schneggenburger R. Allosteric modulation of the presynaptic Ca^2+^ sensor for vesicle fusion. Nature. 2005;435(7041):497–501. 10.1038/nature03568 15917809

[83] Neher E, Sakaba T. Multiple roles of calcium ions in the regulation of neurotransmitter release. Neuron. 2008;59(6):861–872. 10.1016/j.neuron.2008.08.019 18817727

[84] Wan QF, Nixon E, Heidelberger R. Regulation of presynaptic calcium in a mammalian synaptic terminal. J Neurophysiol. 2012;108(11):3059–3067. 10.1152/jn.00213.2012 22972962 PMC3544868

[85] Clements JD. Transmitter timecourse in the synaptic cleft: its role in central synaptic function. Trends Neurosci. 1996;19(5):163–171. 10.1016/S0166-2236(96)10024-2 8723198

[86] Pasti L, Zonta M, Pozzan T, Vicini S, Carmignoto G. Cytosolic calcium oscillations in astrocytes may regulate exocytotic release of glutamate. J Neurosci. 2001;21(2):477–484. 10.1523/JNEUROSCI.21-02-00477.2001 11160427 PMC6763795

[87] Innocenti B, Parpura V, Haydon PG. Imaging extracellular waves of glutamate during calcium signaling in cultured astrocytes. J Neurosci. 2000;20(5):1800–1808. 10.1523/JNEUROSCI.20-05-01800.2000 10684881 PMC6772903

[88] Perea G, Yang A, Boyden ES, Sur M. Optogenetic astrocyte activation modulates response selectivity of visual cortex neurons *in vivo*. Nat Commun. 2014;5:3262 10.1038/ncomms4262 24500276 PMC4075037

[89] Bender KJ, Allen CB, Bender VA, Feldman DE. Synaptic basis for whisker deprivation-induced synaptic depression in rat somatosensory cortex. J Neurosci. 2006;26(16):4155–4165. 10.1523/JNEUROSCI.0175-06.2006 16624936 PMC3070309

[90] Nevian T, Sakmann B. Spine Ca^2+^ signaling in spike-timing-dependent plasticity. J Neurosci. 2006;26(43):11001–11013. 10.1523/JNEUROSCI.1749-06.2006 17065442 PMC6674669

[91] Rodríguez-Moreno A, Kohl MM, Reeve JE, Eaton TR, Collins HA, Anderson HL, et al Presynaptic induction and expression of timing-dependent long-term depression demonstrated by compartment-specific photorelease of a use-dependent NMDA receptor antagonist. J Neurosci. 2011;31(23):8564–8569. 10.1523/JNEUROSCI.0274-11.2011 21653860 PMC4299820

[92] Savtchenko LP, Bard L, Jensen TP, Reynolds JP, Kraev I, Medvedev N, et al Disentangling astroglial physiology with a realistic cell model in silico. Nat Commun. 2018;9(1):3554 10.1038/s41467-018-05896-w 30177844 PMC6120909

[93] Min R, Santello M, Nevian T. The computational power of astrocyte mediated synaptic plasticity. Front Comput Neurosci. 2012;6:93 10.3389/fncom.2012.00093 23125832 PMC3485583

[94] Rougier NP, Hinsen K, Alexandre F, Arildsen T, Barba LA, Benureau FCY, et al Sustainable computational science: the ReScience initiative. PeerJ Comput Sci. 2017;3:e142 10.7717/peerj-cs.142 PMC8530091 34722870

[95] Heifets BD, Castillo PE. Endocannabinoid signaling and long-term synaptic plasticity. Annu Rev Physiol. 2009;71:283–306. 10.1146/annurev.physiol.010908.163149 19575681 PMC4454279

[96] Han J, Kesner P, Metna-Laurent M, Duan T, Xu L, Georges F, et al Acute cannabinoids impair working memory through astroglial CB_1_ receptor modulation of hippocampal LTD. Cell. 2012;148(5):1039–1050. 10.1016/j.cell.2012.01.037 22385967

[97] Navarrete M, Díez A, Araque A. Astrocytes in endocannabinoid signalling. Phil Trans R Soc B. 2014;369(1654):20130599 10.1098/rstb.2013.0599 25225093 PMC4173285

[98] Hegyi Z, Oláh T, Kőszeghy Á, Piscitelli F, Holló K, Pál B, et al CB_1_ receptor activation induces intracellular Ca^2+^ mobilization and 2-arachidonoylglycerol release in rodent spinal cord astrocytes. Sci Rep. 2018;8(1):10562 10.1038/s41598-018-28763-6 30002493 PMC6043539

[99] Jourdain P, Bergersen LH, Bhaukaurally K, Bezzi P, Santello M, Domercq M, et al Glutamate exocytosis from astrocytes controls synaptic strength. Nat Neurosci. 2007;10(3):331–339. 10.1038/nn1849 17310248

[100] Marchaland J, Calì C, Voglmaier SM, Li H, Regazzi R, Edwards RH, et al Fast subplasma membrane Ca^2+^ transients control exo-endocytosis of synaptic-like microvesicles in astrocytes. J Neurosci. 2008;28(37):9122–9132. 10.1523/JNEUROSCI.0040-08.2008 18784293 PMC2846455

[101] Stobart JL, Ferrari KD, Barrett MJP, Glück C, Stobart MJ, Zuend M, et al Cortical circuit activity evokes rapid astrocyte calcium signals on a similar timescale to neurons. Neuron. 2018;98(4):726–735. 10.1016/j.neuron.2018.03.050 29706581

[102] Stobart JL, Ferrari KD, Barrett MJP, Stobart MJ, Looser ZJ, Saab AS, et al Long-term in vivo calcium imaging of astrocytes reveals distinct cellular compartment responses to sensory stimulation. Cereb Cortex. 2018;28(1):184–198. 10.1093/cercor/bhw366 28968832

[103] Agulhon C, Fiacco TA, McCarthy KD. Hippocampal short- and long-term plasticity are not modulated by astrocyte Ca^2+^ signaling. Science. 2010;327(5970):1250–1254. 10.1126/science.1184821 20203048

[104] Chai H, Diaz-Castro B, Shigetomi E, Monte E, Octeau JC, Yu X, et al Neural circuit-specialized astrocytes: transcriptomic, proteomic, morphological, and functional evidence. Neuron. 2017;95(3):531–549. 10.1016/j.neuron.2017.06.029 28712653 PMC5811312

[105] Corlew R, Wang Y, Ghermazien H, Erisir A, Philpot BD. Developmental switch in the contribution of presynaptic and postsynaptic NMDA receptors to long-term depression. J Neurosci. 2007;27(37):9835–9845. 10.1523/JNEUROSCI.5494-06.2007 17855598 PMC2905826

[106] Agulhon C, Sun MY, Murphy T, Myers T, Lauderdale K, Fiacco TA. Calcium signaling and gliotransmission in normal vs. reactive astrocytes. Front Pharmacol. 2012;3:139 10.3389/fphar.2012.00139 22811669 PMC3395812

[107] Santello M, Calì C, Bezzi P. Gliotransmission and the tripartite synapse In: Kreutz M, Sala C, editors. Synaptic Plasticity. Dynamics, Development and Disease. Vienna, Austria: Springer; 2012 p. 307–331. 10.1007/978-3-7091-0932-8_14 22351062

[108] De Pittà M, Brunel N, Volterra A. Astrocytes: orchestrating synaptic plasticity? Neuroscience. 2016;323:43–61. 10.1016/j.neuroscience.2015.04.001 25862587

[109] Brasier DJ, Feldman DE. Synapse-specific expression of functional presynaptic NMDA receptors in rat somatosensory cortex. J Neurosci. 2008;28(9):2199–2211. 10.1523/JNEUROSCI.3915-07.2008 18305253 PMC3071744

[110] Abrahamsson T, Chou CYC, Li SY, Mancino A, Costa RP, Brock JA, et al Differential regulation of evoked and spontaneous release by presynaptic NMDA receptors. Neuron. 2017;96(4):839–855. 10.1016/j.neuron.2017.09.030 29033205

[111] Feldman DE, Brecht M. Map plasticity in somatosensory cortex. Science. 2005;310(5749):810–815. 10.1126/science.1115807 16272113

[112] López-Hidalgo M, Schummers J. Cortical maps: a role for astrocytes? Curr Opin Neurobiol. 2014;24:176–189. 10.1016/j.conb.2013.11.001 24419141

[113] Yang J, Yang H, Liu Y, Li X, Qin L, Lou H, et al Astrocytes contribute to synapse elimination *via* type 2 inositol 1,4,5-trisphosphate receptor-dependent release of ATP. Elife. 2016;5:e15043 10.7554/eLife.15043 27067238 PMC4829431

[114] Nishida H, Okabe S. Direct astrocytic contacts regulate local maturation of dendritic spines. J Neurosci. 2007;27(2):331–340. 10.1523/JNEUROSCI.4466-06.2007 17215394 PMC6672072

[115] Foncelle A, Mendes A, Jedrzejewska-Szmek J, Valtcheva S, Berry H, Blackwell KT, et al Modulation of spike-timing dependent plasticity: towards the inclusion of a third factor in computational models. Front Comput Neurosci. 2018;12:49 10.3389/fncom.2018.00049 30018546 PMC6037788

[116] Gerstner W, Lehmann M, Liakoni V, Corneil D, Brea J. Eligibility traces and plasticity on behavioral time scales: experimental support of neohebbian three-factor learning rules. Front Neural Circuits. 2018;12:53 10.3389/fncir.2018.00053 30108488 PMC6079224

[117] Izhikevich EM. Solving the distal reward problem through linkage of STDP and dopamine signaling. Cereb Cortex. 2007;17(10):2443–2452. 10.1093/cercor/bhl152 17220510

[118] Einevoll GT, Destexhe A, Diesmann M, Grün S, Jirsa V, de Kamps M, et al The scientific case for brain simulations. Neuron. 2019;102(4):735–744. 10.1016/j.neuron.2019.03.027 31121126

[119] Payne HL, French RL, Guo CC, Nguyen-Vu TDB, Manninen T, Raymond JL. Cerebellar Purkinje cells control eye movements with a rapid rate code that is invariant to spike irregularity. Elife. 2019;8:e37102 10.7554/eLife.37102 31050648 PMC6499540

[120] Yu X, Nagai J, Khakh BS. Improved tools to study astrocytes. Nat Rev Neurosci. 2020; p. 1–18. 10.1038/s41583-020-0264-8 32042146

[121] Laviv T, Kim BB, Chu J, Lam AJ, Lin MZ, Yasuda R. Simultaneous dual-color fluorescence lifetime imaging with novel red-shifted fluorescent proteins. Nat Methods. 2016;13(12):989–992. 10.1038/nmeth.4046 27798609 PMC5322478

[122] Handly LN, Yao J, Wollman R. Signal transduction at the single-cell level: approaches to study the dynamic nature of signaling networks. J Mol Biol. 2016;428(19):3669–3682. 10.1016/j.jmb.2016.07.009 27430597 PMC5023475

[123] Badoual M, Zou Q, Davison AP, Rudolph M, Bal T, Fregnac Y, et al Biophysical and phenomenological models of multiple spike interactions in spike-timing dependent plasticity. Int J Neural Syst. 2006;16(2):79–97. 10.1142/S0129065706000524 16688849

[124] Cousin MA, Robinson PJ. The dephosphins: dephosphorylation by calcineurin triggers synaptic vesicle endocytosis. Trends Neurosci. 2001;24(11):659–665. 10.1016/S0166-2236(00)01930-5 11672811

[125] Heifets BD, Chevaleyre V, Castillo PE. Interneuron activity controls endocannabinoid-mediated presynaptic plasticity through calcineurin. Proc Natl Acad Sci USA. 2008;105(29):10250–10255. 10.1073/pnas.0711880105 18632563 PMC2481322

[126] Cottrell JR, Li B, Kyung JW, Ashford CJ, Mann JJ, Horvath TL, et al Calcineurin Aγ is a functional phosphatase that modulates synaptic vesicle endocytosis. J Biol Chem. 2016;291(4):1948–1956. 10.1074/jbc.M115.705319 26627835 PMC4722470

[127] Rodríguez-Moreno A, Banerjee A, Paulsen O. Presynaptic NMDA receptors and spike timing-dependent long-term depression at cortical synapses. Front Synaptic Neurosci. 2010;2:18 10.3389/fnsyn.2010.00018 21423504 PMC3059699

[128] Di Castro MA, Chuquet J, Liaudet N, Bhaukaurally K, Santello M, Bouvier D, et al Local Ca^2+^ detection and modulation of synaptic release by astrocytes. Nat Neurosci. 2011;14(10):1276–1284. 10.1038/nn.2929 21909085

[129] Haustein MD, Kracun S, Lu XH, Shih T, Jackson-Weaver O, Tong X, et al Conditions and constraints for astrocyte calcium signaling in the hippocampal mossy fiber pathway. Neuron. 2014;82(2):413–429. 10.1016/j.neuron.2014.02.041 24742463 PMC4086217

[130] Kanemaru K, Sekiya H, Xu M, Satoh K, Kitajima N, Yoshida K, et al In vivo visualization of subtle, transient, and local activity of astrocytes using an ultrasensitive Ca^2+^ indicator. Cell Rep. 2014;8(1):311–318. 10.1016/j.celrep.2014.05.056 24981861

[131] Rungta RL, Bernier LP, Dissing-Olesen L, Groten CJ, LeDue JM, Ko R, et al Ca^2+^ transients in astrocyte fine processes occur via Ca^2+^ influx in the adult mouse hippocampus. Glia. 2016;64(12):2093–2103. 10.1002/glia.23042 27479868

[132] Fujita T, Chen MJ, Li B, Smith NA, Peng W, Sun W, et al Neuronal transgene expression in dominant-negative SNARE mice. J Neurosci. 2014;34(50):16594–16604. 10.1523/JNEUROSCI.2585-14.2014 25505312 PMC4261088

[133] Sloan SA, Barres BA. Looks can be deceiving: reconsidering the evidence for gliotransmission. Neuron. 2014;84(6):1112–1115. 10.1016/j.neuron.2014.12.003 25521372 PMC4433290

[134] Cahoy JD, Emery B, Kaushal A, Foo LC, Zamanian JL, Christopherson KS, et al A transcriptome database for astrocytes, neurons, and oligodendrocytes: a new resource for understanding brain development and function. J Neurosci. 2008;28(1):264–278. 10.1523/JNEUROSCI.4178-07.2008 18171944 PMC6671143

[135] McDougal RA, Morse TM, Carnevale T, Marenco L, Wang R, Migliore M, et al Twenty years of ModelDB and beyond: building essential modeling tools for the future of neuroscience. J Comput Neurosci. 2017;42(1):1–10. 10.1007/s10827-016-0623-7 27629590 PMC5279891

