# Supplementary material for: Astrocyte-mediated spike-timing-dependent long-term depression modulates synaptic properties in the developing cortex
Source: PLoS Comput Biol. 2020 Nov 10;16(11):e1008360. doi: 10.1371/journal.pcbi.1008360 (PMC7654831; doi:10.1371/journal.pcbi.1008360)
Supplement: S1 Appendix — (PDF) [file pcbi.1008360.s001.pdf]

S1 Appendix

Astrocyte-mediated spike-timing-dependent long-term depression modulates synaptic properties in the developing cortex

Tiina Manninen, Ausra Saudargiene, Marja-Leena Linne

tiina.manninen@tuni.fi, marja-leena.linne@tuni.fi

To study the role of astrocytes in modulation of spike-timing-dependent long-term depression (t-LTD) in somatosensory cortex, we simulated a layer 4 (L4) to layer 2/3 (L2/3) synapse model. In the model, we described major biophysical and biochemical mechanisms for the one-compartmental presynaptic L4 spiny stellate cell, two-compartmental (soma and dendrite) postsynaptic L2/3 pyramidal cell, and one-compartmental nearby fine astrocyte process. We chose to include the most important signaling pathways that were assumed to be crucial in explaining t-LTD at L4-L2/3 synapse. We stimulated our synapse model using t-LTD stimulation protocols with a varying temporal difference between post- and presynaptic activity.

The model components are shortly the following:

- Presynaptic neuron model
  - High-voltage-activated (HVA) N-type calcium (Ca^{2+}) (Ca_{NHVA}) channel [1]
  - Potassium (K^+) channel [2]
  - Sodium (Na^+) channel [2]
  - Leak channel [2]
  - N-methyl-D-aspartate receptor (NMDAR) composed of GluN1 and either GluN2C or GluN2D subunits (GluN2C/D-containing NMDAR) [3, 4]
  - Calcineurin (CaN) signaling [5]
  - CaN-dependence to available glutamate (Glu) release
  - Glu release model modified from [6–10]
- Postsynaptic neuron model
  - L-type HVA Ca^{2+} (Ca_{LHVA}) channel [11, 12]
  - Low-voltage-activated (LVA) L-type Ca^{2+} (Ca_{LLVA}) channel [13]
  - A-type K^+ (K_A) channel [14]
  - Delayed rectifier K^+ (K_{DR}) channel [14]
  - Na^+ channel [14]
  - Persistent Na^+ (Na_P) channel [14]
  - Leak channel [14, 15]
  - Coupling currents [14, 15]

- α-amino-3-hydroxy-5-methyl-4-isoxazolepropionic acid receptor (AMPAR) [16]
- NMDAR composed of GluN1 and GluN2B subunits (GluN2B-containing NMDAR) [16]
- Metabotropic Glu receptor (mGluR) [17]
- Inositol 1,4,5-trisphosphate (IP_3) receptor (IP_3R) [18–20]
- Plasma membrane Ca^{2+}-ATPase (PMCA pump) [21]
- Sarco/endoplasmic reticulum (ER) Ca^{2+}-ATPase (SERCA pump) [18, 19]
- Leak flux [18, 19, 21]
- Ca^{2+} buffer [22]
- Reactions from mGluR activation to 2-arachidonoylglycerol (2-AG) release [17, 22]

- Astrocyte model
  - IP_3 activation by postsynaptic 2-AG modified from [23]
  - IP_3R [18, 19]
  - SERCA pump [18, 19]
  - Leak flux [18, 19]
  - Glu release model [6–10]

Stimulation Protocols and Simulation Results

The following stimulation protocols were used [24]:

1. The stimulation protocol before t-LTD induction consisted of five 10 ms long presynaptic stimuli at a frequency of 0.2 Hz and with an amplitude of 10 $\frac{\mu A}{cm^2}$ keeping the fraction of presynaptic Glu release inhibition (f_{pre}) as constant zero.

2. The t-LTD induction protocol consisted of a 10 ms long postsynaptic stimulus with an amplitude of 25 $\frac{\mu A}{cm^2}$ occurring between 10 ms and 200 ms before a 10 ms long presynaptic stimulus with an amplitude of 10 $\frac{\mu A}{cm^2}$ and the post-pre pairing was repeated 100 times at a frequency of 0.2 Hz. Thus, the temporal difference (ΔT) between the pre- and postsynaptic stimulus in this study had negative values meaning that the postsynaptic stimulus occurred before the presynaptic stimulus: ΔT had values between −10 ms and −200 ms. Note that in simulations ΔT values were implemented as positive because we set the postsynaptic stimuli to occur at fixed time points in all the simulations whereas the presynaptic stimuli occurred in varying time points after the postsynaptic stimuli depending on ΔT, and not the other way around. The initial value of f_{pre} in these simulations was zero, but it increased during the stimulation protocol to above zero depending on ΔT used in the t-LTD induction protocol.

3. The stimulation protocol after t-LTD induction consisted of five 10 ms long presynaptic stimuli at a frequency of 0.2 Hz and with an amplitude of 10 $\frac{\mu A}{cm^2}$ keeping f_{pre} as constant value received from the t-LTD induction protocol. For the fraction f_{pre}, we used the final simulation value received from the simulation with the t-LTD induction protocol. Thus, f_{pre} had different constant values depending on ΔT used in the t-LTD induction protocol.

Computational Model

The used abbreviations can be found in Table A. The values used throughout the synapse model for certain parameters are given in Table B.

Presynaptic neuron model

We extended a previously published presynaptic one-compartmental neuron model [2] by adding (1) $\mathrm{Ca_{NHVA}}$ channels [1], (2) GluN2C/D-containing NMDARs [3, 4], (3) $\mathrm{Ca^{2+}}$ signaling [1], (4) CaN signaling [5], (5) CaN-dependence to available Glu release, and (6) Glu release to the synaptic cleft (modified from [6–10]).

The differential equation for the presynaptic membrane potential can be given as [2]

$$C_{\mathrm{m,pre}}\frac{\mathrm{d}V_{\mathrm{pre}}}{\mathrm{d}t} = -I_{\mathrm{CaNHVA,pre}} - I_{\mathrm{K,pre}} - I_{\mathrm{Na,pre}} - I_{\mathrm{L,pre}} - I_{\mathrm{Ca,NMDAR,pre}} - I_{\mathrm{Na,NMDAR,pre}} + I_{\mathrm{ext,pre}} \ , \quad (1)$$

where $C_{\mathrm{m,pre}}$ is the presynaptic membrane capacitance per unit area, $I_{\mathrm{CaNHVA,pre}}$ is the current density via $\mathrm{Ca_{NHVA}}$ channels, $I_{\mathrm{K,pre}}$ is the $\mathrm{K^+}$ current density, $I_{\mathrm{Na,pre}}$ is the $\mathrm{Na^+}$ current density, $I_{\mathrm{L,pre}}$ is the leak current density, $I_{\mathrm{Ca,NMDAR,pre}}$ and $I_{\mathrm{Na,NMDAR,pre}}$ are the $\mathrm{Ca^{2+}}$ and $\mathrm{Na^+}$ current densities via NMDARs, and $I_{\mathrm{ext,pre}}$ is the stimulus current injected into the presynaptic neuron per unit area. The channels presented in Eq (1) are described by the Hodgkin-Huxley and Goldman-Hodgkin-Katz formalisms [1, 2]. The differential equations for the gating variables x ($m_{\mathrm{CaNHVA,pre}}$, $h_{\mathrm{CaNHVA,pre}}$, $n_{\mathrm{K,pre}}$, $n_{\mathrm{K,2,pre}}$, $m_{\mathrm{Na,pre}}$, and $h_{\mathrm{Na,pre}}$) of different currents presented in Eq (1) can be given as [1, 2]

$$\frac{\mathrm{d}x}{\mathrm{d}t} = \frac{x_\infty - x}{\tau_x} \ , \quad (2)$$

where x_∞ and τ_x denote the steady-state values and time constants for different gating variables, respectively. For the gating variable $s_{\mathrm{Na,pre}}$, the differential equation is [2]

$$\frac{\mathrm{d}s_{\mathrm{Na,pre}}}{\mathrm{d}t} = \frac{s_{\infty,\mathrm{Na,pre}} - s_{\mathrm{Na,pre}}}{\tau_{s,\mathrm{Na,pre}}\sigma_{s,\mathrm{Na,pre}} + \tau_{sb,\mathrm{Na,pre}}} \ , \quad (3)$$

where $s_{\infty,\mathrm{Na,pre}}$ denotes the steady-state value, $\tau_{s,\mathrm{Na,pre}}$ and $\tau_{sb,\mathrm{Na,pre}}$ denote the time constants, and $\sigma_{s,\mathrm{Na,pre}}$ denotes a shift in the time constant.

Presynaptic $\mathrm{Ca^{2+}}$ concentrations are increased by $\mathrm{Ca^{2+}}$ influxes through presynaptic NMDARs and $\mathrm{Ca_{NHVA}}$ channels. The NMDAR-mediated $\mathrm{Ca^{2+}}$ concentration activates CaN, and then CaN inhibits vesicle exocytosis and Glu release from the presynaptic neuron [25, 26]. The concentration of $\mathrm{Ca^{2+}}$ mediated by $\mathrm{Ca_{NHVA}}$ channels activates vesicle exocytosis and Glu release from the presynaptic neuron. The differential equation for the presynaptic $\mathrm{Ca^{2+}}$ concentration through $\mathrm{Ca_{NHVA}}$ channels is [27]

$$\frac{\mathrm{d}[\mathrm{Ca^{2+}}]_{\mathrm{CaNHVA,pre}}}{\mathrm{d}t} = -\frac{I_{\mathrm{CaNHVA,pre}}}{c_{\mathrm{Ca,pre}}} + \frac{\mathrm{Ca_{rest,pre}} - [\mathrm{Ca^{2+}}]_{\mathrm{CaNHVA,pre}}}{\tau_{\mathrm{Ca,pre}}} \quad (4)$$

and the differential equation for the presynaptic $\mathrm{Ca^{2+}}$ concentration mediated by NMDARs is [27]

$$\frac{\mathrm{d}[\mathrm{Ca^{2+}}]_{\mathrm{NMDAR,pre}}}{\mathrm{d}t} = -\frac{I_{\mathrm{Ca,NMDAR,pre}}}{c_{\mathrm{Ca,pre}}} + \frac{\mathrm{Ca_{rest,pre}} - [\mathrm{Ca^{2+}}]_{\mathrm{NMDAR,pre}}}{\tau_{\mathrm{Ca,pre}}} \ , \quad (5)$$

where $c_{\mathrm{Ca,pre}}$, $\mathrm{Ca_{rest,pre}}$, and $\tau_{\mathrm{Ca,pre}}$ are the presynaptic scaling factor to convert from units $\frac{\mathrm{\mu A}}{\mathrm{cm^2}}$ to $\frac{\mathrm{\mu M}}{\mathrm{ms}}$, presynaptic resting $\mathrm{Ca^{2+}}$ concentration, and time constant of a

presynaptic pump, respectively. The presynaptic pump takes into account the effect of SERCA and PMCA pumps as well as Na^{+}/Ca^{2+} exchangers [27].

The presynaptic NMDAR-mediated Ca^{2+} influx presented in Eq (5) activates presynaptic CaN [26], and the differential equation for the presynaptic CaN concentration can be given as [5]

$$\frac{\mathrm{d}[\mathrm{CaN}]_{\mathrm{pre}}}{\mathrm{d}t} = k_{1,\mathrm{pre}} \left(\mathrm{CaN}_{\mathrm{max,pre}} - [\mathrm{CaN}]_{\mathrm{pre}}\right) [\mathrm{Ca}^{2+}]^{3}_{\mathrm{NMDAR,pre}} - k_{2,\mathrm{pre}}[\mathrm{CaN}]_{\mathrm{pre}} \ , \quad (6)$$

where $k_{1,\mathrm{pre}}$ and $k_{2,\mathrm{pre}}$ are the rate constants for the activation and inactivation of presynaptic CaN, respectively, and $\mathrm{CaN}_{\mathrm{max,pre}}$ is the total concentration of presynaptic CaN. Calcineurin has been shown to regulate a specific phase of synaptic vesicle cycling, thus influencing the vesicle release [28–31]. We modeled the effect of CaN to Glu release via a signaling pathway linking CaN to vesicle release and recycling in the presynaptic terminal with the following differential equation

$$\frac{\mathrm{d}[\mathrm{X}]_{\mathrm{ac,pre}}}{\mathrm{d}t} = p_{1,\mathrm{pre}} \frac{[\mathrm{CaN}]_{\mathrm{pre}}^{n_{2,\mathrm{pre}}}}{K_{\mathrm{A,pre}}^{n_{2,\mathrm{pre}}} + [\mathrm{CaN}]_{\mathrm{pre}}^{n_{2,\mathrm{pre}}}} \left(\mathrm{X}_{\mathrm{total,pre}} - [\mathrm{X}]_{\mathrm{ac,pre}}\right) \ , \quad (7)$$

where $[\mathrm{X}]_{\mathrm{ac,pre}}$ is the active concentration and $\mathrm{X}_{\mathrm{total,pre}}$ is the total concentration of the unspecified protein that affects the vesicle release, $p_{1,\mathrm{pre}}$ is the rate constant, $K_{\mathrm{A,pre}}$ is the CaN concentration producing half occupation, and $n_{2,\mathrm{pre}}$ is the Hill coefficient.

The differential equation for the fraction of releasable presynaptic vesicles is [7–10]

$$\frac{\mathrm{d}R_{\mathrm{rel,pre}}}{\mathrm{d}t} = k_{\mathrm{recov,pre}} \left(1 - R_{\mathrm{rel,pre}}\right) - \sum_{j} P_{\mathrm{rel,pre}} R_{\mathrm{rel,pre}} \delta(t - t_j) \quad (8)$$

and the differential equation for the release probability of presynaptic Glu vesicles was combined and modified from previously published equations [7–10] and is given as

$$\begin{aligned} \frac{\mathrm{d}P_{\mathrm{rel,pre}}}{\mathrm{d}t} = & - k_{\mathrm{f,pre}} P_{\mathrm{rel,pre}} \\ & + \sum_{j} \left(1 - f_{\mathrm{pre}}\right) \frac{[\mathrm{Ca}^{2+}]_{\mathrm{CaNHVA,pre}}^{n_{1,\mathrm{pre}}}}{K_{\mathrm{rel,pre}}^{n_{1,\mathrm{pre}}} + [\mathrm{Ca}^{2+}]_{\mathrm{CaNHVA,pre}}^{n_{1,\mathrm{pre}}}} \left(1 - P_{\mathrm{rel,pre}}\right) \delta(t - t_j) \ , \end{aligned} \quad (9)$$

where the fraction (f_{pre}), which is the active concentration ($[\mathrm{X}]_{\mathrm{ac,pre}}$) divided by the total concentration ($\mathrm{X}_{\mathrm{total,pre}}$) of the protein, affects the probability of presynaptic Glu release. Note that f_{pre} inhibits $P_{\mathrm{rel,pre}}$ and excites $R_{\mathrm{rel,pre}}$. Parameters $k_{\mathrm{recov,pre}}$, $k_{\mathrm{f,pre}}$, $K_{\mathrm{rel,pre}}$, and $n_{1,\mathrm{pre}}$ describe the presynaptic recovery rate constant from empty to releasable state, facilitation rate constant, Ca^{2+} concentration producing half occupation used in calculation of Glu release, and Hill coefficient, respectively. The differential equation for the Glu concentration in the synaptic cleft was combined and modified from previously published Glu equations [8–10] and Glu-activated postsynaptic equations related to mGluRs [17] and is given as

$$\begin{aligned} \frac{\mathrm{d}[\mathrm{Glu}]_{\mathrm{syncleft}}}{\mathrm{d}t} = & - v_{\mathrm{Glu,f,post}} - v_{\mathrm{mGluR,f,post}} + v_{\mathrm{mGluR,b,post}} \\ & + \sum_{j} \frac{G_{\mathrm{pre}} N_{\mathrm{pre}} P_{\mathrm{rel,pre}} R_{\mathrm{rel,pre}}}{k_{\mathrm{Glu,pre}} N_{\mathrm{A}} V_{\mathrm{syncleft}}} \delta(t - t_j) \ , \end{aligned} \quad (10)$$

where $v_{\mathrm{Glu,f,post}}$, $v_{\mathrm{mGluR,f,post}}$, and $v_{\mathrm{mGluR,b,post}}$ are the reaction rates for the postsynaptic mGluR Glu uptake, and postsynaptic mGluR Glu binding and unbinding, respectively. Parameters G_{pre}, N_{pre}, $k_{\mathrm{Glu,pre}}$, N_{A}, and V_{syncleft} denote the number of

Glu per presynaptic vesicle, number of readily releasable presynaptic vesicles, scaling factor to convert from units M to µM, Avogadro's constant, and volume of synaptic cleft, respectively. In Eq (8)–(10), the presynaptic Glu release occurs at the first time point $t = t_j$ such that $[\mathrm{Ca}^{2+}]_{\mathrm{CaNHVA,pre}} \geq C_{\mathrm{thr,pre}}$ and less than 10 ms has passed from the previous presynaptic membrane potential crossing 0 mV from negative to positive voltages ($V_{\mathrm{pre}} \geq 0$, $\frac{\mathrm{d}V_{\mathrm{pre}}}{\mathrm{d}t} > 0$) at that time point t_j. The δ function has units of $\frac{1}{\mathrm{ms}}$.

Experimental studies have reported that presynaptic GluN2C/D-containing NMDARs are required for t-LTD at L4 to L2/3 synapses [24, 32–36], whereas postsynaptic GluN2B-containing NMDARs are necessary for spike-timing-dependent long-term potentiation (t-LTP) at L4-L2/3 and L2/3-L2/3 synapses, and postsynaptic GluN2A-containing NMDARs are required in t-LTD at L2/3-L2/3 synapses [24, 32–37]. Thus, we modeled presynaptic GluN2C/D-containing NMDARs [3, 4]. The closed presynaptic NMDAR (represented in Eq (11) as fraction $\mathrm{R}_{\mathrm{pre}}$) binds two agonists (represented in equations as A), such as Glu, and yields to $\mathrm{R\_A}_{2,\mathrm{pre}}$. In our synapse model, presynaptic NMDARs are activated by the sum of available Glu in the synaptic cleft and extrasynaptic space, where the Glu concentration in the synaptic cleft is multiplied by a parameter $f_{\mathrm{Glu,pre}}$ to denote the effect of spillover of glutamate from the synaptic cleft to the extrasynaptic space and that not all Glu in the synaptic cleft activates presynaptic NMDARs (most part of Glu in the synaptic cleft activates the postsynaptic receptors). The differential equations for these variables are [3, 4]

$$\frac{\mathrm{dR}_{\mathrm{pre}}}{\mathrm{d}t} = -v_{\mathrm{2kon,pre}} + v_{\mathrm{koff,pre}} \ , \tag{11}$$

$$\frac{\mathrm{dR\_A}_{\mathrm{pre}}}{\mathrm{d}t} = v_{\mathrm{2kon,pre}} - v_{\mathrm{koff,pre}} - v_{\mathrm{kon,pre}} + v_{\mathrm{2koff,pre}} \ , \tag{12}$$

and

$$\begin{aligned}\frac{\mathrm{dR\_A}_{2,\mathrm{pre}}}{\mathrm{d}t} = {} & v_{\mathrm{kon,pre}} - v_{\mathrm{2koff,pre}} - v_{\mathrm{d1,f,pre}} + v_{\mathrm{d1,b,pre}} - v_{\mathrm{d2,f,pre}} \\ & + v_{\mathrm{d2,b,pre}} - v_{\mathrm{f1,f,pre}} + v_{\mathrm{f1,b,pre}} - v_{\mathrm{s1,f,pre}} + v_{\mathrm{s1,b,pre}} \ .\end{aligned} \tag{13}$$

Glutamate available for the presynaptic NMDARs is present in some of the reaction rates in Eq (11)–(13).

Then $\mathrm{R\_A}_{2,\mathrm{pre}}$ form of the NMDARs in Eq (13) can either become one of the two desensitized states (represented in Eq (14) as $\mathrm{R\_A}_{2,\mathrm{d1,pre}}$ or in Eq (15) as $\mathrm{R\_A}_{2,\mathrm{d2,pre}}$), or go through a faster conformational change of the GluN1 subunit of the NMDAR (represented in Eq (16) as $\mathrm{R\_A}_{2,\mathrm{f,pre}}$) or a slower conformational change of the GluN2 subunit of the NMDAR (represented in Eq (17) as $\mathrm{R\_A}_{2,\mathrm{s,pre}}$). The differential equations for these variables are [3, 4]

$$\frac{\mathrm{dR\_A}_{2,\mathrm{d1,pre}}}{\mathrm{d}t} = v_{\mathrm{d1,f,pre}} - v_{\mathrm{d1,b,pre}} \ , \tag{14}$$

$$\frac{\mathrm{dR\_A}_{2,\mathrm{d2,pre}}}{\mathrm{d}t} = v_{\mathrm{d2,f,pre}} - v_{\mathrm{d2,b,pre}} \ , \tag{15}$$

$$\frac{\mathrm{dR\_A}_{2,\mathrm{f,pre}}}{\mathrm{d}t} = v_{\mathrm{f1,f,pre}} - v_{\mathrm{f1,b,pre}} - v_{\mathrm{s2,f,pre}} + v_{\mathrm{s2,b,pre}} \ , \tag{16}$$

and

$$\frac{\mathrm{dR\_A}_{2,\mathrm{s,pre}}}{\mathrm{d}t} = v_{\mathrm{s1,f,pre}} - v_{\mathrm{s1,b,pre}} - v_{\mathrm{f2,f,pre}} + v_{\mathrm{f2,b,pre}} \ . \tag{17}$$

The slow and fast components can proceed towards magnesium (Mg^{2+})-unblocked (open) NMDAR state and the differential equation for it can be given as [3, 4]

$$\frac{\mathrm{dR\_A}_{2,\mathrm{O,pre}}}{\mathrm{d}t} = v_{\mathrm{s2,f,pre}} - v_{\mathrm{s2,b,pre}} + v_{\mathrm{f2,f,pre}} - v_{\mathrm{f2,b,pre}} - v_{\mathrm{Mg,f,pre}} + v_{\mathrm{Mg,b,pre}} \ . \tag{18}$$

Similarly to Eq (11)–(18), NMDAR equations with Mg^{2+} blocking can be given as [4]

$$\frac{\mathrm{dR\_Mg_{pre}}}{\mathrm{d}t} = -v_{\mathrm{2konMg,pre}} + v_{\mathrm{koffMg,pre}} \ , \tag{19}$$

$$\frac{\mathrm{dR\_A\_Mg_{pre}}}{\mathrm{d}t} = v_{\mathrm{2konMg,pre}} - v_{\mathrm{koffMg,pre}} - v_{\mathrm{konMg,pre}} + v_{\mathrm{2koffMg,pre}} \ , \tag{20}$$

$$\begin{aligned}\frac{\mathrm{dR\_A_2\_Mg_{pre}}}{\mathrm{d}t} = & \, v_{\mathrm{konMg,pre}} - v_{\mathrm{2koffMg,pre}} - v_{\mathrm{d1Mg,f,pre}} + v_{\mathrm{d1Mg,b,pre}} \\ & - v_{\mathrm{d2Mg,f,pre}} + v_{\mathrm{d2Mg,b,pre}} - v_{\mathrm{f1Mg,f,pre}} \\ & + v_{\mathrm{f1Mg,b,pre}} - v_{\mathrm{s1Mg,f,pre}} + v_{\mathrm{s1Mg,b,pre}} \ ,\end{aligned} \tag{21}$$

$$\frac{\mathrm{dR\_A_{2,d1}\_Mg_{pre}}}{\mathrm{d}t} = v_{\mathrm{d1Mg,f,pre}} - v_{\mathrm{d1Mg,b,pre}} \ , \tag{22}$$

$$\frac{\mathrm{dR\_A_{2,d2}\_Mg_{pre}}}{\mathrm{d}t} = v_{\mathrm{d2Mg,f,pre}} - v_{\mathrm{d2Mg,b,pre}} \ , \tag{23}$$

$$\frac{\mathrm{dR\_A_{2,f}\_Mg_{pre}}}{\mathrm{d}t} = v_{\mathrm{f1Mg,f,pre}} - v_{\mathrm{f1Mg,b,pre}} - v_{\mathrm{s2Mg,f,pre}} + v_{\mathrm{s2Mg,b,pre}} \ , \tag{24}$$

$$\frac{\mathrm{dR\_A_{2,s}\_Mg_{pre}}}{\mathrm{d}t} = v_{\mathrm{s1Mg,f,pre}} - v_{\mathrm{s1Mg,b,pre}} - v_{\mathrm{f2Mg,f,pre}} + v_{\mathrm{f2Mg,b,pre}} \ , \tag{25}$$

and, finally, the Mg^{2+}-blocked NMDAR state can be given as [4]

$$\begin{aligned}\frac{\mathrm{dR\_A_{2,O}\_Mg_{pre}}}{\mathrm{d}t} = & \, v_{\mathrm{s2Mg,f,pre}} - v_{\mathrm{s2Mg,b,pre}} + v_{\mathrm{f2Mg,f,pre}} \\ & - v_{\mathrm{f2Mg,b,pre}} + v_{\mathrm{Mg,f,pre}} - v_{\mathrm{Mg,b,pre}} \ .\end{aligned} \tag{26}$$

The intermediate variables needed to solve Eq (1)–(26) can be found in Tables C, D, and F. The list of presynaptic reactions can be found in Table E. The parameter values of the presynaptic model can be found in Tables G–J, and initial values in Table K.

Postsynaptic neuron model

We modified a previously published postsynaptic two-compartmental neuron model [15] by adopting (1) K_A, K_{DR}, Na^+, and Na_P channels [14], (2) Ca_{LHVA} channels [11, 12], (3) Ca_{LLVA} channels [13], (4) AMPARs [16], (5) GluN2B-containing NMDARs [16], (6) mGluR activation to endocannabinoid 2-AG release [17, 22], and (7) Ca^{2+} signaling [18, 19, 21, 22].

The postsynaptic neuron model for the two compartments, a soma and a dendrite, was modified from a previously published study [15]. The differential equations for the membrane potentials of these two compartments are

$$\begin{aligned}C_{\mathrm{m,post}}\frac{\mathrm{d}V_{\mathrm{soma,post}}}{\mathrm{d}t} = & - I_{\mathrm{KDR,soma,post}} - I_{\mathrm{Na,soma,post}} - I_{\mathrm{NaP,soma,post}} \\ & - I_{\mathrm{L,soma,post}} + I_{\mathrm{coupl,soma,post}} + I_{\mathrm{ext,post}}\end{aligned} \tag{27}$$

and

$$\begin{aligned}C_{\mathrm{m,post}}\frac{\mathrm{d}V_{\mathrm{dend,post}}}{\mathrm{d}t} = & - I_{\mathrm{KA,dend,post}} - I_{\mathrm{CaLHVA,dend,post}} - I_{\mathrm{CaLLVA,dend,post}} \\ & - I_{\mathrm{Na,dend,post}} - I_{\mathrm{L,dend,post}} - I_{\mathrm{AMPAR,post}} \\ & - I_{\mathrm{Ca,NMDAR,post}} + I_{\mathrm{coupl,dend,post}} \ ,\end{aligned} \tag{28}$$

where $C_{\mathrm{m,post}}$ is the membrane capacitance per unit area, $I_{\mathrm{KDR,soma,post}}$ is the somatic K_{DR} current density, $I_{\mathrm{Na,soma,post}}$ and $I_{\mathrm{Na,dend,post}}$ are the somatic and dendritic Na^+

current densities, $I_{\mathrm{NaP,soma,post}}$ is the somatic $\mathrm{Na_P}$ current density, $I_{\mathrm{L,soma,post}}$ and $I_{\mathrm{L,dend,post}}$ are the somatic and dendritic leak current densities, $I_{\mathrm{coupl,soma,post}}$ and $I_{\mathrm{coupl,dend,post}}$ are the somatic and dendritic coupling terms, $I_{\mathrm{ext,post}}$ is the current injected into the soma per unit area, $I_{\mathrm{KA,dend,post}}$ is the dendritic $\mathrm{K_A}$ current density, $I_{\mathrm{CaLHVA,dend,post}}$ and $I_{\mathrm{CaLLVA,dend,post}}$ are the dendritic $\mathrm{Ca_{LHVA}}$ and $\mathrm{Ca_{LLVA}}$ current densities, and $I_{\mathrm{AMPAR,post}}$ and $I_{\mathrm{Ca,NMDAR,post}}$ are the synaptic current densities via AMPARs and NMDARs in the dendrite. We used Hodgkin-Huxley formalism to describe the behavior of ionic currents [11–14].

The differential equations for the fractions of postsynaptic AMPARs and GluN2B-containing NMDARs in open state can be modified from a previously published study [16] and given as

$$\frac{\mathrm{d}m_{\mathrm{AMPAR,post}}}{\mathrm{d}t} = \alpha_{\mathrm{AMPAR,post}}(1 - f_{\mathrm{Glu,pre}})[\mathrm{Glu}]_{\mathrm{syncleft}}(1 - m_{\mathrm{AMPAR,post}}) - \beta_{\mathrm{AMPAR,post}} m_{\mathrm{AMPAR,post}} \tag{29}$$

and

$$\frac{\mathrm{d}m_{\mathrm{NMDAR,post}}}{\mathrm{d}t} = \alpha_{\mathrm{NMDAR,post}}(1 - f_{\mathrm{Glu,pre}})[\mathrm{Glu}]_{\mathrm{syncleft}}(1 - m_{\mathrm{NMDAR,post}}) - \beta_{\mathrm{NMDAR,post}} m_{\mathrm{NMDAR,post}} \ , \tag{30}$$

where $\alpha_{\mathrm{AMPAR,post}}$ and $\beta_{\mathrm{AMPAR,post}}$ describe the rate constants of opening and closing postsynaptic AMPARs, respectively, and similarly to NMDARs. Parameter $1 - f_{\mathrm{Glu,pre}}$ denotes the part of Glu in synaptic cleft that activates the postsynaptic receptors. Other differential equations for the gating variables x ($m_{\mathrm{KDR,soma,post}}$, $m_{\mathrm{Na,soma,post}}$, $h_{\mathrm{Na,soma,post}}$, $m_{\mathrm{CaLHVA,dend,post}}$, $h_{\mathrm{CaLHVA,dend,post}}$, $m_{\mathrm{CaLLVA,dend,post}}$, $h_{\mathrm{CaLLVA,dend,post}}$, $m_{\mathrm{KA,dend,post}}$, $h_{\mathrm{KA,dend,post}}$, $m_{\mathrm{Na,dend,post}}$, and $h_{\mathrm{Na,dend,post}}$) of different currents presented in Eq (27) and (28) and for the $\mathrm{IP_3R}$ inactivation gating variable ($h_{\mathrm{IP3R,post}}$) can be given as [11–14, 18, 19]

$$\frac{\mathrm{d}x}{\mathrm{d}t} = \frac{x_\infty - x}{\tau_x} \ , \tag{31}$$

where x_∞ and τ_x denote the steady-state values and time constants for different gating variables, respectively.

The biochemical mechanisms related to mGluRs and the activation of the G-protein signaling cascade, as well as the subsequent production of endocannabinoids are included in the synapse model to study the effect of endocannabinoids on the nearby astrocyte. Next, we present the differential equations starting from the mGluR activation to the endocannabinoid 2-AG release [17, 22].

Glutamate in the synaptic cleft activates postsynaptic mGluRs and induces dissociation of the G protein α subunit bound with guanosine-5'-triphosphate (GαGTP) from the mGluR-bound G protein with β and γ subunits (Gβγ). The differential equation for the concentration of the postsynaptic mGluRs is [17]

$$\frac{\mathrm{d}[\mathrm{mGluR}]_{\mathrm{post}}}{\mathrm{d}t} = -v_{\mathrm{mGluR,f,post}} + v_{\mathrm{mGluR,b,post}} \ , \tag{32}$$

for the concentration of the Glu-mGluR complex is [17]

$$\frac{\mathrm{d}[\mathrm{Glu\_mGluR}]_{\mathrm{post}}}{\mathrm{d}t} = v_{\mathrm{mGluR,f,post}} - v_{\mathrm{mGluR,b,post}} - v_{\mathrm{mGluRdes,f,post}} + v_{\mathrm{mGluRdes,b,post}} - v_{\mathrm{Gact,f,post}} + v_{\mathrm{Gact,b,post}} + v_{\mathrm{Gact,c,post}} \ , \tag{33}$$

and for the concentration of the desensitized Glu-mGluR complex is [17]

$$\frac{\mathrm{d[Glu\_mGluRdesens]_{post}}}{\mathrm{d}t} = v_{\mathrm{mGluRdes,f,post}} - v_{\mathrm{mGluRdes,b,post}}\ . \tag{34}$$

The differential equation for the concentration of G protein with α, β, and γ subunits (Gαβγ) is [17]

$$\frac{\mathrm{d[G\alpha\beta\gamma]_{post}}}{\mathrm{d}t} = -v_{\mathrm{Gact,f,post}} + v_{\mathrm{Gact,b,post}} + v_{\mathrm{regenG,f,post}} \tag{35}$$

and for Gαβγ bound with Glu and mGluRs is [17]

$$\frac{\mathrm{d[G\alpha\beta\gamma\_Glu\_mGluR]_{post}}}{\mathrm{d}t} = v_{\mathrm{Gact,f,post}} - v_{\mathrm{Gact,b,post}} - v_{\mathrm{Gact,c,post}}\ . \tag{36}$$

The differential equation for the concentration of GαGTP can be given as [17]

$$\begin{aligned}\frac{\mathrm{d[G\alpha GTP]_{post}}}{\mathrm{d}t} = {} & v_{\mathrm{Gact,c,post}} - v_{\mathrm{G\_PLC2,f,post}} + v_{\mathrm{G\_PLC2,b,post}} \\ & - v_{\mathrm{G\_PLC1,f,post}} + v_{\mathrm{G\_PLC1,b,post}} - v_{\mathrm{hydrG,f,post}}\ .\end{aligned} \tag{37}$$

Calcium binds to phospholipase C (PLC), and GαGTP can enhance its activity. The differential equation for the concentration of postsynaptic Ca^{2+} is [17–19, 21]

$$\begin{aligned}\frac{\mathrm{d[Ca^{2+}]_{post}}}{\mathrm{d}t} = {} & - v_{\mathrm{Ca\_PLC1,f,post}} + v_{\mathrm{Ca\_PLC1,b,post}} - v_{\mathrm{Ca\_PLC2,f,post}} \\ & + v_{\mathrm{Ca\_PLC2,b,post}} - v_{\mathrm{DAGL,f,post}} + v_{\mathrm{DAGL,b,post}} \\ & + J_{\mathrm{IP3R,post}} - J_{\mathrm{SERCA,post}} + J_{\mathrm{leakER,post}} + J_{\mathrm{CaL,post}} \\ & + J_{\mathrm{NMDAR,post}} - J_{\mathrm{PMCA,post}} + J_{\mathrm{leakCell,post}}\end{aligned} \tag{38}$$

and for the concentration of Ca^{2+} in the ER is [18, 19, 21]

$$\frac{\mathrm{d[Ca^{2+}]_{ER,post}}}{\mathrm{d}t} = \frac{-J_{\mathrm{IP3R,post}} + J_{\mathrm{SERCA,post}} - J_{\mathrm{leakER,post}}}{r_{\mathrm{ERcyt,post}}}\ , \tag{39}$$

where $J_{\mathrm{IP3R,post}}$ is the Ca^{2+} flux via IP_3Rs from the ER into the cytosol, $J_{\mathrm{SERCA,post}}$ is the Ca^{2+} flux via SERCA pumps from the cytosol into the ER, $J_{\mathrm{leakER,post}}$ is the Ca^{2+} leak flux from the ER into the cytosol, $J_{\mathrm{CaL,post}}$ is the combined flux via Ca_{LHVA} and Ca_{LLVA} channels, $J_{\mathrm{NMDAR,post}}$ is the Ca^{2+} flux via NMDARs, $J_{\mathrm{PMCA,post}}$ is the Ca^{2+} flux via PMCA pumps, and $J_{\mathrm{leakCell,post}}$ is the leak flux from the extracellular space to the cytosol. Parameter $r_{\mathrm{ERcyt,post}}$ denotes the ratio between postsynaptic ER and cytosol volumes. The differential equation for the concentration of PLC can be given as [17]

$$\begin{aligned}\frac{\mathrm{d[PLC]_{post}}}{\mathrm{d}t} = {} & - v_{\mathrm{Ca\_PLC1,f,post}} + v_{\mathrm{Ca\_PLC1,b,post}} - v_{\mathrm{G\_PLC1,f,post}} \\ & + v_{\mathrm{G\_PLC1,b,post}} + v_{\mathrm{GAP1,f,post}}\end{aligned} \tag{40}$$

and for the concentration of the Ca^{2+}-PLC complex can be given as [17]

$$\begin{aligned}\frac{\mathrm{d[Ca\_PLC]_{post}}}{\mathrm{d}t} = {} & v_{\mathrm{Ca\_PLC1,f,post}} - v_{\mathrm{Ca\_PLC1,b,post}} - v_{\mathrm{G\_PLC2,f,post}} \\ & + v_{\mathrm{G\_PLC2,b,post}} - v_{\mathrm{DAG1,f,post}} + v_{\mathrm{DAG1,b,post}} \\ & + v_{\mathrm{DAG2,f,post}} + v_{\mathrm{GAP2,f,post}}\ .\end{aligned} \tag{41}$$

The differential equation for the concentration of the Ca^{2+}-GαGTP-PLC complex is [17]

$$\begin{aligned}\frac{\mathrm{d}[\mathrm{Ca\_G\alpha GTP\_PLC}]_{\mathrm{post}}}{\mathrm{d}t} = {} & v_{\mathrm{G\_PLC2,f,post}} - v_{\mathrm{G\_PLC2,b,post}} + v_{\mathrm{Ca\_PLC2,f,post}} \\ & - v_{\mathrm{Ca\_PLC2,b,post}} - v_{\mathrm{DAG3,f,post}} + v_{\mathrm{DAG3,b,post}} \\ & + v_{\mathrm{DAG4,f,post}} - v_{\mathrm{GAP2,f,post}}\end{aligned} \tag{42}$$

and for the concentration of the GαGTP-PLC complex is [17]

$$\begin{aligned}\frac{\mathrm{d}[\mathrm{G\alpha GTP\_PLC}]_{\mathrm{post}}}{\mathrm{d}t} = {} & v_{\mathrm{G\_PLC1,f,post}} - v_{\mathrm{G\_PLC1,b,post}} - v_{\mathrm{Ca\_PLC2,f,post}} \\ & + v_{\mathrm{Ca\_PLC2,b,post}} - v_{\mathrm{GAP1,f,post}} \ .\end{aligned} \tag{43}$$

Active PLC can then produce IP_3 and diacylglycerol (DAG) from phosphatidylinositol 4,5-bisphosphate (PIP_2). The differential equation for the concentration of PIP_2 is [17]

$$\begin{aligned}\frac{\mathrm{d}[\mathrm{PIP_2}]_{\mathrm{post}}}{\mathrm{d}t} = {} & - v_{\mathrm{DAG1,f,post}} + v_{\mathrm{DAG1,b,post}} - v_{\mathrm{DAG3,f,post}} \\ & + v_{\mathrm{DAG3,b,post}} + v_{\mathrm{PIP2,c,post}} \ ,\end{aligned} \tag{44}$$

for the concentration of the Ca^{2+}-PIP_2-PLC complex is [17]

$$\frac{\mathrm{d}[\mathrm{Ca\_PIP_2\_PLC}]_{\mathrm{post}}}{\mathrm{d}t} = v_{\mathrm{DAG1,f,post}} - v_{\mathrm{DAG1,b,post}} - v_{\mathrm{DAG1,c,post}} \ , \tag{45}$$

and for the concentration of the Ca^{2+}-DAG-PLC complex is [17]

$$\frac{\mathrm{d}[\mathrm{Ca\_DAG\_PLC}]_{\mathrm{post}}}{\mathrm{d}t} = v_{\mathrm{DAG1,c,post}} - v_{\mathrm{DAG2,f,post}} \ . \tag{46}$$

The differential equation for the concentration of the Ca^{2+}-GαGTP-PIP_2-PLC complex is [17]

$$\frac{\mathrm{d}[\mathrm{Ca\_G\alpha GTP\_PIP_2\_PLC}]_{\mathrm{post}}}{\mathrm{d}t} = v_{\mathrm{DAG3,f,post}} - v_{\mathrm{DAG3,b,post}} - v_{\mathrm{DAG3,c,post}} \tag{47}$$

and for the concentration of the Ca^{2+}-DAG-GαGTP-PLC complex is [17]

$$\frac{\mathrm{d}[\mathrm{Ca\_DAG\_G\alpha GTP\_PLC}]_{\mathrm{post}}}{\mathrm{d}t} = v_{\mathrm{DAG3,c,post}} - v_{\mathrm{DAG4,f,post}} \ . \tag{48}$$

The differential equation for the concentration of the G protein with α subunit bound with guanosine diphosphate (GαGDP) can be given as [17]

$$\frac{\mathrm{d}[\mathrm{G\alpha GDP}]_{\mathrm{post}}}{\mathrm{d}t} = v_{\mathrm{GAP1,f,post}} + v_{\mathrm{GAP2,f,post}} + v_{\mathrm{hydrG,f,post}} - v_{\mathrm{regenG,f,post}} \ . \tag{49}$$

The differential equation for the concentration of IP_3 is [17]

$$\frac{\mathrm{d}[\mathrm{IP_3}]_{\mathrm{post}}}{\mathrm{d}t} = v_{\mathrm{DAG1,c,post}} + v_{\mathrm{DAG3,c,post}} - v_{\mathrm{degIP3,post}} \tag{50}$$

and for the concentration of DAG is [17]

$$\begin{aligned}\frac{\mathrm{d}[\mathrm{DAG}]_{\mathrm{post}}}{\mathrm{d}t} = {} & v_{\mathrm{DAG2,f,post}} + v_{\mathrm{DAG4,f,post}} - v_{\mathrm{prodAG,f,post}} \\ & + v_{\mathrm{prodAG,b,post}} - v_{\mathrm{degDAG,post}} \ .\end{aligned} \tag{51}$$

After IP_3 is degraded, PIP_2 is regenerated by phosphoinositide 3-kinase (PIKin). The differential equation for the concentration of the degraded IP_3 (IP_3deg) is [17]

$$\frac{\mathrm{d}[\mathrm{IP_3deg}]_{\mathrm{post}}}{\mathrm{d}t} = v_{\mathrm{degIP3,post}} - v_{\mathrm{PIP2,f,post}} + v_{\mathrm{PIP2,b,post}} \ , \tag{52}$$

for the concentration of PIKin is [17]

$$\frac{\mathrm{d}[\mathrm{PIKin}]_{\mathrm{post}}}{\mathrm{d}t} = -v_{\mathrm{PIP2,f,post}} + v_{\mathrm{PIP2,b,post}} + v_{\mathrm{PIP2,c,post}} \ , \tag{53}$$

and for the concentration of the IP_3deg-PIKin complex is [17]

$$\frac{\mathrm{d}[\mathrm{IP_3deg\_PIKin}]_{\mathrm{post}}}{\mathrm{d}t} = v_{\mathrm{PIP2,f,post}} - v_{\mathrm{PIP2,b,post}} - v_{\mathrm{PIP2,c,post}} \ . \tag{54}$$

After Ca^{2+} binds to DAG lipase (DAGL), the Ca^{2+}-DAGL complex binds to DAG and 2-AG synthesis is catalyzed. The equation for the concentration of DAGL is [17]

$$\frac{\mathrm{d}[\mathrm{DAGL}]_{\mathrm{post}}}{\mathrm{d}t} = -v_{\mathrm{DAGL,f,post}} + v_{\mathrm{DAGL,b,post}} \ , \tag{55}$$

for the concentration of the Ca^{2+}-DAGL complex is [17]

$$\begin{aligned}\frac{\mathrm{d}[\mathrm{Ca\_DAGL}]_{\mathrm{post}}}{\mathrm{d}t} &= v_{\mathrm{DAGL,f,post}} - v_{\mathrm{DAGL,b,post}} - v_{\mathrm{prodAG,f,post}} \\ &\quad + v_{\mathrm{prodAG,b,post}} + v_{\mathrm{prodAG,c,post}} \ ,\end{aligned} \tag{56}$$

for the concentration of the Ca^{2+}-DAG-DAGL complex is [17]

$$\frac{\mathrm{d}[\mathrm{Ca\_DAG\_DAGL}]_{\mathrm{post}}}{\mathrm{d}t} = v_{\mathrm{prodAG,f,post}} - v_{\mathrm{prodAG,b,post}} - v_{\mathrm{prodAG,c,post}} \ , \tag{57}$$

and, finally, for the concentration of 2-AG is [17]

$$\frac{\mathrm{d}[\mathrm{2\text{-}AG}]_{\mathrm{post}}}{\mathrm{d}t} = v_{\mathrm{prodAG,c,post}} - v_{\mathrm{degAG,post}} \ . \tag{58}$$

The intermediate variables of the postsynaptic model needed to solve Eq (27)–(58) can be found in Tables L–O and Table Q. The list of postsynaptic reactions can be found in Table P. The parameter values of the postsynaptic model can be found in Tables R–T, and initial values in Tables U and V.

Astrocyte Model

For the astrocyte model, we utilized previously published [18, 19, 38] and extensively tested [39–42] Ca^{2+} signaling models by adding a modified version of a previously published model for IP_3 dependence on endocannabinoids [23] and a model for Glu release to the extrasynaptic space [6–10].

We modeled the Ca^{2+} and IP_3 concentrations, and the gating variable for IP_3R inactivation (h_{astro}) in the astrocyte, and the Glu concentration in the extrasynaptic space. The differential equation for the astrocytic Ca^{2+} concentration is [18, 19]

$$\frac{\mathrm{d}[\mathrm{Ca^{2+}}]_{\mathrm{astro}}}{\mathrm{d}t} = J_{\mathrm{IP3R,astro}} - J_{\mathrm{SERCA,astro}} + J_{\mathrm{leakER,astro}} \ , \tag{59}$$

where $J_{\mathrm{IP3R,astro}}$ is the Ca^{2+} flux via IP_3Rs from the ER into the cytosol, $J_{\mathrm{SERCA,astro}}$ is the Ca^{2+} flux via SERCA pumps from the cytosol into the ER, and $J_{\mathrm{leakER,astro}}$ is the

Ca^{2+} leak flux from the ER into the cytosol. The differential equation for the astrocytic IP_3 concentration was modified from previously published equations [23, 38] and given as

$$\frac{\mathrm{d}[\mathrm{IP}_3]_{\mathrm{astro}}}{\mathrm{d}t} = \frac{\mathrm{IP}^{\star}_{3,\mathrm{astro}} - [\mathrm{IP}_3]_{\mathrm{astro}}}{\tau_{\mathrm{IP3,astro}}} + r_{\mathrm{IP3,astro}}([\text{2-AG}]_{\mathrm{post}} - \mathrm{AG}^{\star}_{\mathrm{post}}) \ , \tag{60}$$

where $\mathrm{IP}^{\star}_{3,\mathrm{astro}}$, $\tau_{\mathrm{IP3,astro}}$, $r_{\mathrm{IP3,astro}}$, $[\text{2-AG}]_{\mathrm{post}}$, and $\mathrm{AG}^{\star}_{\mathrm{post}}$ denote the resting concentration of IP_3, time constant for IP_3 degradation, rate constant of IP_3 production, concentration of the endocannabinoid 2-AG released from the postsynaptic neuron, and resting concentration of 2-AG, respectively. The differential equation for the astrocytic IP_3R inactivation gating variable is given as [19]

$$\frac{\mathrm{d}h_{\mathrm{astro}}}{\mathrm{d}t} = \frac{h_{\infty,\mathrm{astro}} - h_{\mathrm{astro}}}{\tau_{h,\mathrm{astro}}} \ , \tag{61}$$

where $h_{\infty,\mathrm{astro}}$ is the steady-state value of the IP_3R inactivation gating variable and $\tau_{h,\mathrm{astro}}$ is the time constant for the IP_3R inactivation.

We modeled the astrocytic Glu release as exocytosis [43–45]. The differential equation for the fraction of releasable Glu resources in astrocyte is [7–10]

$$\frac{\mathrm{d}R_{\mathrm{rel,astro}}}{\mathrm{d}t} = k_{\mathrm{recov,astro}}\,(1 - R_{\mathrm{rel,astro}}) - \sum_i P_{\mathrm{rel,astro}} R_{\mathrm{rel,astro}} \delta(t - \tau_i) \tag{62}$$

and the Glu concentration in the extrasynaptic space is [9, 10]

$$\begin{aligned}\frac{\mathrm{d}[\mathrm{Glu}]_{\mathrm{extsyn}}}{\mathrm{d}t} =& - r_{\mathrm{astro}}[\mathrm{Glu}]_{\mathrm{extsyn}} \\ &+ \sum_i r_{\mathrm{vesext,astro}} G_{\mathrm{astro}} N_{\mathrm{astro}} P_{\mathrm{rel,astro}} R_{\mathrm{rel,astro}} \delta(t - \tau_i) \ ,\end{aligned} \tag{63}$$

where $k_{\mathrm{recov,astro}}$ is the recovery rate constant from empty to releasable state, r_{astro} is the Glu clearance rate from the extrasynaptic space, $r_{\mathrm{vesext,astro}}$ is the ratio between the astrocytic vesicular volume and the volume of the extrasynaptic space, G_{astro} is the Glu concentration per astrocytic vesicle, and N_{astro} is the number of readily releasable astrocytic vesicles. The astrocytic Glu release occurs at the first time point $t = \tau_i$ such that $[\mathrm{Ca}^{2+}]_{\mathrm{astro}} \geq C_{\mathrm{thr,astro}}$ and $\frac{\mathrm{d}[\mathrm{Ca}^{2+}]_{\mathrm{astro}}}{\mathrm{d}t} > 0$ at that time point τ_i. The δ function has units of $\frac{1}{\mathrm{ms}}$.

Intermediate variables needed to solve Eq (59)–(63) can be found in Table W, parameter values in Table X, and initial values in Table Y.

Table A. Abbreviations.

Abbreviation	Explanation	Abbreviation	Explanation
2-AG	2-arachidonoylglycerol	IP_3deg	Degraded IP_3
AMPAR	α-amino-3-hydroxy-5-methyl-4-isoxazolepropionic acid receptor	IP_3R	IP_3 receptor
ATP	Adenosine triphosphate	K^+	Potassium ion
Ca^{2+}	Calcium ion	K_A	A-type K^+ channel
CaN	Calcineurin	K_{DR}	Delayed rectifier K^+ channel
Ca_{LHVA}	L-type HVA Ca^{2+} channel	L2/3	Layer 2/3
Ca_{LLVA}	L-type LVA Ca^{2+} channel	L4	Layer 4
Ca_{NHVA}	N-type HVA Ca^{2+} channel	LTD	Long-term depression
CB_1R	Type 1 cannabinoid receptor	LTP	Long-term potentiation
DAG	Diacylglycerol	LVA	Low-voltage-activated
DAGL	DAG lipase	Mg^{2+}	Magnesium ion
EPSP	Excitatory postsynaptic potential	mGluR	Metabotropic Glu receptor
ER	Endoplasmic reticulum	mGluRdesens	Desensitized mGluR
G	G protein	Na^+	Sodium ion
Gα	G protein with α subunit	Na_P	Persistent Na^+ channel
Gαβγ	G protein with α, β, and γ subunits	NMDAR	N-methyl-D-aspartate receptor
GαGDP	G protein with α subunit bound with GDP	PIKin	Phosphoinositide 3-kinase
GαGTP	G protein with α subunit bound with GTP	PIP_2	Phosphatidylinositol 4,5-bisphosphate
Gβγ	G protein with β and γ subunits	PLC	Phospholipase C
GAP	GTPase activating protein	PMCA	Plasma membrane Ca^{2+}-ATPase
GDP	Guanosine diphosphate	SERCA	Sarco/ER Ca^{2+}-ATPase
Glu	Glutamate	STDP	Spike-timing-dependent plasticity
GluN2C/D	NMDAR containing GluN2C or GluN2D subunits	t-LTD	Spike-timing-dependent LTD
GTP	Guanosine-5'-triphosphate	t-LTP	Spike-timing-dependent LTP
HVA	High-voltage-activated	ΔEPSP	Change in EPSP
IP_3	Inositol 1,4,5-trisphosphate	ΔT	Temporal difference between the pre- and postsynaptic activity

Table B. Parameter values.

Name	Value	Unit	Description	Refs
$A_{\mathrm{stim,post}}$	25	$\frac{\mu A}{cm^2}$	External current amplitude to postsynaptic neuron per unit area	
$A_{\mathrm{stim,pre}}$	10	$\frac{\mu A}{cm^2}$	External current amplitude to presynaptic neuron per unit area	
F	96,485	$\frac{C}{mol}$	Faraday constant	
N_{A}	6.0221×10^{23}	$\frac{1}{mol}$	Avogadro's constant	
R	8.3145	$\frac{J}{Kmol}$	Molar gas constant	
T_{celsius}	36	℃	Temperature	[14]
z	2	1	Valence of Ca^{2+} ion	

Table C. Intermediate variables of presynaptic channel model.

Intermediate variable	Description	Refs
$\sigma_{s,\mathrm{Na,pre}} = \frac{1}{1+\exp\left(\frac{V_{\mathrm{pre}}+V_{sv,\mathrm{Na,pre}}+V_{\mathrm{shift,pre}}}{V_{sd,\mathrm{Na,pre}}}\right)}$	Shift in presynaptic Na^{+} inactivation time constant	[2]
$h_{\infty,\mathrm{Na,pre}} = \frac{1}{1+\exp\left(\frac{V_{\mathrm{pre}}+45+V_{\mathrm{shift,pre}}}{3}\right)}$	Steady-state value of presynaptic Na^{+} inactivation gating variable	[2]
$m_{\infty,\mathrm{Na,pre}} = \frac{1}{1+\exp\left(-\frac{V_{\mathrm{pre}}+40+V_{\mathrm{shift,pre}}}{3}\right)}$	Steady-state value of presynaptic Na^{+} activation gating variable	[2]
$s_{\infty,\mathrm{Na,pre}} = \frac{1}{1+\exp\left(\frac{V_{\mathrm{pre}}+44+V_{\mathrm{shift,pre}}}{3}\right)}$	Steady-state value of presynaptic Na^{+} inactivation gating variable	[2]
$n_{\infty,\mathrm{K,pre}} = \frac{1}{1+\exp\left(-\frac{V_{\mathrm{pre}}+40+V_{\mathrm{shift,pre}}}{3}\right)}$	Steady-state value of presynaptic K^{+} activation gating variable	[2]
$n_{\infty,\mathrm{K,2,pre}} = \frac{1}{1+\exp\left(-\frac{V_{\mathrm{pre}}+40+V_{\mathrm{shift,pre}}}{3}\right)}$	Steady-state value of presynaptic K^{+} activation gating variable	[2]
$\alpha_{h,\mathrm{CaNHVA,pre}} = 0.00016\exp\left(-\frac{V_{\mathrm{pre}}}{48.4}\right)$	Transition rate of presynaptic Ca_{NHVA} channel's inactivation gate from non-permissive to permissive state	[1]
$\alpha_{m,\mathrm{CaNHVA,pre}} = 0.1967\frac{-V_{\mathrm{pre}}+19.88}{\exp\left(\frac{-V_{\mathrm{pre}}+19.88}{10}\right)-1}$	Transition rate of presynaptic Ca_{NHVA} channel's activation gate from non-permissive to permissive state	[1]
$\alpha_{mt,\mathrm{CaNHVA,pre}} = \exp\left(0.0378 z_{m,\mathrm{pre}}\left(V_{\mathrm{pre}}-V_{1/2m,\mathrm{pre}}\right)\right)$	Rate used in calculation of presynaptic Ca_{NHVA} activation time constant	[1]
$\beta_{h,\mathrm{CaNHVA,pre}} = \frac{1}{1+\exp\left(\frac{-V_{\mathrm{pre}}+39}{10}\right)}$	Transition rate of presynaptic Ca_{NHVA} channel's inactivation gate from permissive to non-permissive state	[1]
$\beta_{m,\mathrm{CaNHVA,pre}} = 0.046\exp\left(-\frac{V_{\mathrm{pre}}}{20.73}\right)$	Transition rate of presynaptic Ca_{NHVA} channel's activation gate from permissive to non-permissive state	[1]
$\beta_{mt,\mathrm{CaNHVA,pre}} = \exp\left(0.0378 z_{m,\mathrm{pre}} g_{mm,\mathrm{pre}}\left(V_{\mathrm{pre}}-V_{1/2m,\mathrm{pre}}\right)\right)$	Rate used in calculation of presynaptic Ca_{NHVA} activation time constant	[1]
$h_{\infty,\mathrm{CaNHVA,pre}} = \frac{\alpha_{h,\mathrm{CaNHVA,pre}}}{\alpha_{h,\mathrm{CaNHVA,pre}}+\beta_{h,\mathrm{CaNHVA,pre}}}$	Steady-state value of presynaptic Ca_{NHVA} inactivation gating variable	[1]
$m_{\infty,\mathrm{CaNHVA,pre}} = \frac{\alpha_{m,\mathrm{CaNHVA,pre}}}{\alpha_{m,\mathrm{CaNHVA,pre}}+\beta_{m,\mathrm{CaNHVA,pre}}}$	Steady-state value of presynaptic Ca_{NHVA} activation gating variable	[1]
$h_{\infty,\mathrm{CaNHVA,2,pre}} = \frac{K_{\mathrm{inh,pre}}}{K_{\mathrm{inh,pre}}+[\mathrm{Ca}^{2+}]_{\mathrm{CaNHVA,pre}}}$	Steady-state value of presynaptic Ca_{NHVA} inactivation gating variable	[1]
$\tau_{m,\mathrm{CaNHVA,pre}} = \max\left(\frac{\tau_{m,\mathrm{min,pre}}}{5^{\frac{T_{\mathrm{celsius}}-25}{10}}}, \frac{\beta_{mt,\mathrm{CaNHVA,pre}}}{5^{\frac{T_{\mathrm{celsius}}-25}{10}} a_{0m,\mathrm{pre}}\left(1+\alpha_{mt,\mathrm{CaNHVA,pre}}\right)}\right)$	Time constant for presynaptic Ca_{NHVA} activation	[1]

Table D. Presynaptic channel and receptor currents.

Intermediate variable	Description	Refs
$\nu_{\text{pre}} = \dfrac{V_{\text{pre}}}{c_{V,\text{pre}}}$	Scaling of presynaptic membrane potential used in Goldman-Hodgkin-Katz current equation (unitless)	[1]
$e_{\text{pre}} = \begin{cases} \frac{\nu_{\text{pre}}}{2} - 1, & \text{if } \lvert\nu_{\text{pre}}\rvert < 0.0001 \\ \frac{\nu_{\text{pre}}}{1-\exp(\nu_{\text{pre}})}, & \text{otherwise} \end{cases}$	Equation dealing with Goldman-Hodgkin-Katz current equation near 0 mV and otherwise	[1]
$f_{\text{ghk,pre}} = c_{V,\text{pre}} \left(1 - \dfrac{[\text{Ca}^{2+}]_{\text{CaNHVA,pre}}}{\text{Ca}_{\text{ext,pre}}} \exp\left(\nu_{\text{pre}}\right)\right) e_{\text{pre}}$	Driving force of Goldman-Hodgkin-Katz current equation used in calculation of presynaptic Ca_{NHVA} current	[1]
$I_{\text{CaNHVA,pre}} = g_{\text{CaNHVA,pre}} m^2_{\text{CaNHVA,pre}} h_{\text{CaNHVA,pre}} h_{\infty,\text{CaNHVA},2,\text{pre}} f_{\text{ghk,pre}}$	Presynaptic Ca_{NHVA} current density	[1]
$I_{\text{K,pre}} = g_{\text{K,pre}} n^3_{\text{K,pre}} \left(V_{\text{pre}} - V_{\text{K,pre}}\right) + g_{\text{K,2,pre}} n^3_{\text{K,2,pre}} \left(V_{\text{pre}} - V_{\text{K,pre}}\right)$	Presynaptic K^+ current density	[2]
$I_{\text{L,pre}} = g_{\text{L,pre}} \left(V_{\text{pre}} - V_{\text{L,pre}}\right)$	Presynaptic leak current density	[2]
$I_{\text{Na,pre}} = g_{\text{Na,pre}} m^3_{\text{Na,pre}} h_{\text{Na,pre}} s_{\text{Na,pre}} (V_{\text{pre}} - V_{\text{Na,pre}})$	Presynaptic Na^+ current density	[2]
$I_{\text{Ca,NMDAR,pre}} = \begin{cases} 0, & \text{if } V_{\text{pre}} \geq V_{\text{NMDAR,pre}} \\ 0.1 g_{\text{NMDAR,pre}} \text{R\_A}_{2,\text{O,pre}} \times \\ (V_{\text{pre}} - V_{\text{NMDAR,pre}}), & \text{otherwise} \end{cases}$	Ca^{2+} current density via presynaptic NMDARs	[46, 47]
$I_{\text{Na,NMDAR,pre}} = \begin{cases} 0, & \text{if } V_{\text{pre}} \geq V_{\text{NMDAR,pre}} \\ 0.9 g_{\text{NMDAR,pre}} \text{R\_A}_{2,\text{O,pre}} \times \\ (V_{\text{pre}} - V_{\text{NMDAR,pre}}), & \text{otherwise} \end{cases}$	Na^+ current density via presynaptic NMDARs	[46, 47]

Table E. Reactions of presynaptic NMDAR model.

Reaction	Description	Refs
$Glu_{NMDAR,pre} + R_{pre} \rightleftharpoons R\_A_{pre}$	Presynaptic NMDAR agonist (Glu) binding	[3, 4]
$Glu_{NMDAR,pre} + R\_A_{pre} \rightleftharpoons R\_A_{2,pre}$	Presynaptic NMDAR agonist (Glu) binding	[3, 4]
$R\_A_{2,pre} \rightleftharpoons R\_A_{2,d1,pre}$	Desensitized state of presynaptic NMDAR	[3, 4]
$R\_A_{2,pre} \rightleftharpoons R\_A_{2,d2,pre}$	Desensitized state of presynaptic NMDAR	[3, 4]
$R\_A_{2,pre} \rightleftharpoons R\_A_{2,f,pre}$	Gating-associated conformational changes of presynaptic GluN1 subunit of NMDAR	[3, 4]
$R\_A_{2,pre} \rightleftharpoons R\_A_{2,s,pre}$	Gating-associated conformational changes of presynaptic GluN2 subunit of NMDAR	[3, 4]
$R\_A_{2,f,pre} \rightleftharpoons R\_A_{2,O,pre}$	Gating-associated conformational changes of presynaptic GluN2 subunit of NMDAR	[3, 4]
$R\_A_{2,s,pre} \rightleftharpoons R\_A_{2,O,pre}$	Gating-associated conformational changes of presynaptic GluN1 subunit of NMDAR	[3, 4]
$Glu_{NMDAR,pre} + R\_Mg_{pre} \rightleftharpoons R\_A\_Mg_{pre}$	Presynaptic Mg^{2+}-blocked NMDAR agonist (Glu) binding	[3, 4]
$Glu_{NMDAR,pre} + R\_A\_Mg_{pre} \rightleftharpoons R\_A_2\_Mg_{pre}$	Presynaptic Mg^{2+}-blocked NMDAR agonist (Glu) binding	[3, 4]
$R\_A_2\_Mg_{pre} \rightleftharpoons R\_A_{2,d1}\_Mg_{pre}$	Desensitized state of presynaptic Mg^{2+}-blocked NMDAR	[3, 4]
$R\_A_2\_Mg_{pre} \rightleftharpoons R\_A_{2,d2}\_Mg_{pre}$	Desensitized state of presynaptic Mg^{2+}-blocked NMDAR	[3, 4]
$R\_A_2\_Mg_{pre} \rightleftharpoons R\_A_{2,f}\_Mg_{pre}$	Gating-associated conformational changes of presynaptic GluN1 subunit of Mg^{2+}-blocked NMDAR	[3, 4]
$R\_A_2\_Mg_{pre} \rightleftharpoons R\_A_{2,s}\_Mg_{pre}$	Gating-associated conformational changes of presynaptic GluN2 subunit of Mg^{2+}-blocked NMDAR	[3, 4]
$R\_A_{2,f}\_Mg_{pre} \rightleftharpoons R\_A_{2,O}\_Mg_{pre}$	Gating-associated conformational changes of presynaptic GluN2 subunit of Mg^{2+}-blocked NMDAR	[3, 4]
$R\_A_{2,s}\_Mg_{pre} \rightleftharpoons R\_A_{2,O}\_Mg_{pre}$	Gating-associated conformational changes of presynaptic GluN1 subunit of Mg^{2+}-blocked NMDAR	[3, 4]
$R\_A_{2,O,pre} \rightleftharpoons R\_A_{2,O}\_Mg_{pre}$	Gating-associated conformational changes of presynaptic GluN1 subunit of Mg^{2+}-blocked NMDAR	[3, 4]

Table F. Intermediate variables of presynaptic Glu release model and NMDAR model.

Intermediate variable	Description	Refs
$f_{\mathrm{pre}} = \dfrac{[\mathrm{X}]_{\mathrm{ac,pre}}}{\mathrm{X}_{\mathrm{total,pre}}}$	Fraction of presynaptic Glu release inhibition	
$[\mathrm{Glu}]_{\mathrm{NMDAR,pre}} = f_{\mathrm{Glu,pre}}[\mathrm{Glu}]_{\mathrm{syncleft}} + [\mathrm{Glu}]_{\mathrm{extsyn}}$	Total concentration of Glu that presynaptic NMDARs receives	
$k_{\mathrm{Mg,f,pre}} = 0.00061 \exp\left(-\dfrac{V_{\mathrm{pre}}}{17}\right)$	Rate of presynaptic NMDAR blocking by external Mg^{2+}	[48]
$k_{\mathrm{Mg,b,pre}} = 5.4 \exp\left(\dfrac{V_{\mathrm{pre}}}{47}\right)$	Rate of presynaptic NMDAR unblocking by external Mg^{2+}	[48]
$k_{\mathrm{s,f,pre}} = k_{\mathrm{s,f,0,pre}} \exp\left(\dfrac{V_{\mathrm{pre}} + 100}{175}\right)$	Rate of gating-associated conformational changes of presynaptic GluN2 subunit of NMDAR	[4]
$v_{\mathrm{2kon,pre}} = 2k_{\mathrm{on,pre}}[\mathrm{Glu}]_{\mathrm{NMDAR,pre}}\mathrm{R}_{\mathrm{pre}}$<br>$v_{\mathrm{koff,pre}} = k_{\mathrm{off,pre}}\mathrm{R\_A}_{\mathrm{pre}}$	Reaction rates of Glu binding and unbinding to presynaptic NMDAR	[3, 4]
$v_{\mathrm{kon,pre}} = k_{\mathrm{on,pre}}[\mathrm{Glu}]_{\mathrm{NMDAR,pre}}\mathrm{R\_A}_{\mathrm{pre}}$<br>$v_{\mathrm{2koff,pre}} = 2k_{\mathrm{off,pre}}\mathrm{R\_A}_{\mathrm{2,pre}}$	Reaction rates of Glu binding and unbinding to presynaptic NMDAR	[3, 4]
$v_{\mathrm{d1,f,pre}} = k_{\mathrm{d1,f,pre}}\mathrm{R\_A}_{\mathrm{2,pre}}$<br>$v_{\mathrm{d1,b,pre}} = k_{\mathrm{d1,b,pre}}\mathrm{R\_A}_{\mathrm{2,d1,pre}}$	Reaction rates into and out of desensitized state of presynaptic NMDAR	[3, 4]
$v_{\mathrm{d2,f,pre}} = k_{\mathrm{d2,f,pre}}\mathrm{R\_A}_{\mathrm{2,pre}}$<br>$v_{\mathrm{d2,b,pre}} = k_{\mathrm{d2,b,pre}}\mathrm{R\_A}_{\mathrm{2,d2,pre}}$	Reaction rates into and out of desensitized state of presynaptic NMDAR	[3, 4]
$v_{\mathrm{f1,f,pre}} = k_{\mathrm{f,f,pre}}\mathrm{R\_A}_{\mathrm{2,pre}}$<br>$v_{\mathrm{f1,b,pre}} = k_{\mathrm{f,b,pre}}\mathrm{R\_A}_{\mathrm{2,f,pre}}$	Reaction rates of gating-associated conformational changes of presynaptic GluN1 subunit of NMDAR	[3, 4]
$v_{\mathrm{s1,f,pre}} = k_{\mathrm{s,f,pre}}\mathrm{R\_A}_{\mathrm{2,pre}}$<br>$v_{\mathrm{s1,b,pre}} = k_{\mathrm{s,b,pre}}\mathrm{R\_A}_{\mathrm{2,s,pre}}$	Reaction rates of gating-associated conformational changes of presynaptic GluN2 subunit of NMDAR	[3, 4]
$v_{\mathrm{s2,f,pre}} = k_{\mathrm{s,f,pre}}\mathrm{R\_A}_{\mathrm{2,f,pre}}$<br>$v_{\mathrm{s2,b,pre}} = k_{\mathrm{s,b,pre}}\mathrm{R\_A}_{\mathrm{2,O,pre}}$	Reaction rates of gating-associated conformational changes of presynaptic GluN2 subunit of NMDAR	[3, 4]
$v_{\mathrm{f2,f,pre}} = k_{\mathrm{f,f,pre}}\mathrm{R\_A}_{\mathrm{2,s,pre}}$<br>$v_{\mathrm{f2,b,pre}} = k_{\mathrm{f,b,pre}}\mathrm{R\_A}_{\mathrm{2,O,pre}}$	Reaction rates of gating-associated conformational changes of presynaptic GluN1 subunit of NMDAR	[3, 4]
$v_{\mathrm{2konMg,pre}} = 2k_{\mathrm{on,pre}}[\mathrm{Glu}]_{\mathrm{NMDAR,pre}}\mathrm{R\_Mg}_{\mathrm{pre}}$<br>$v_{\mathrm{koffMg,pre}} = k_{\mathrm{off,pre}}\mathrm{R\_A\_Mg}_{\mathrm{pre}}$	Reaction rates of Glu binding and unbinding to presynaptic Mg^{2+}-blocked NMDAR	[3, 4]
$v_{\mathrm{konMg,pre}} = k_{\mathrm{on,pre}}[\mathrm{Glu}]_{\mathrm{NMDAR,pre}}\mathrm{R\_A\_Mg}_{\mathrm{pre}}$<br>$v_{\mathrm{2koffMg,pre}} = 2k_{\mathrm{off,pre}}\mathrm{R\_A_2\_Mg}_{\mathrm{pre}}$	Reaction rates of Glu binding and unbinding to presynaptic Mg^{2+}-blocked NMDAR	[3, 4]
$v_{\mathrm{d1Mg,f,pre}} = k_{\mathrm{d1,f,pre}}\mathrm{R\_A_2\_Mg}_{\mathrm{pre}}$<br>$v_{\mathrm{d1Mg,b,pre}} = k_{\mathrm{d1,b,pre}}\mathrm{R\_A_{2,d1}\_Mg}_{\mathrm{pre}}$	Reaction rates into and out of desensitized state of presynaptic Mg^{2+}-blocked NMDAR	[3, 4]
$v_{\mathrm{d2Mg,f,pre}} = k_{\mathrm{d2,f,pre}}\mathrm{R\_A_2\_Mg}_{\mathrm{pre}}$<br>$v_{\mathrm{d2Mg,b,pre}} = k_{\mathrm{d2,b,pre}}\mathrm{R\_A_{2,d2}\_Mg}_{\mathrm{pre}}$	Reaction rates into and out of desensitized state of presynaptic Mg^{2+}-blocked NMDAR	[3, 4]
$v_{\mathrm{f1Mg,f,pre}} = k_{\mathrm{f,f,pre}}\mathrm{R\_A_2\_Mg}_{\mathrm{pre}}$<br>$v_{\mathrm{f1Mg,b,pre}} = k_{\mathrm{f,b,pre}}\mathrm{R\_A_{2,f}\_Mg}_{\mathrm{pre}}$	Reaction rates of gating-associated conformational changes of presynaptic GluN1 subunit of Mg^{2+}-blocked NMDAR	[3, 4]
$v_{\mathrm{s1Mg,f,pre}} = k_{\mathrm{s,f,pre}}\mathrm{R\_A_2\_Mg}_{\mathrm{pre}}$<br>$v_{\mathrm{s1Mg,b,pre}} = k_{\mathrm{s,b,pre}}\mathrm{R\_A_{2,s}\_Mg}_{\mathrm{pre}}$	Reaction rates of gating-associated conformational changes of presynaptic GluN2 subunit of Mg^{2+}-blocked NMDAR	[3, 4]
$v_{\mathrm{s2Mg,f,pre}} = k_{\mathrm{s,f,pre}}\mathrm{R\_A_{2,f}\_Mg}_{\mathrm{pre}}$<br>$v_{\mathrm{s2Mg,b,pre}} = k_{\mathrm{s,b,pre}}\mathrm{R\_A_{2,O}\_Mg}_{\mathrm{pre}}$	Reaction rates of gating-associated conformational changes of presynaptic GluN2 subunit of Mg^{2+}-blocked NMDAR	[3, 4]
$v_{\mathrm{f2Mg,f,pre}} = k_{\mathrm{f,f,pre}}\mathrm{R\_A_{2,s}\_Mg}_{\mathrm{pre}}$<br>$v_{\mathrm{f2Mg,b,pre}} = k_{\mathrm{f,b,pre}}\mathrm{R\_A_{2,O}\_Mg}_{\mathrm{pre}}$	Reaction rates of gating-associated conformational changes of presynaptic GluN1 subunit of Mg^{2+}-blocked NMDAR	[3, 4]
$v_{\mathrm{Mg,f,pre}} = k_{\mathrm{Mg,f,pre}}\mathrm{R\_A}_{\mathrm{2,O,pre}}$<br>$v_{\mathrm{Mg,b,pre}} = k_{\mathrm{Mg,b,pre}}\mathrm{R\_A_{2,O}\_Mg}_{\mathrm{pre}}$	Reaction rates of presynaptic NMDAR blocking and unblocking by external Mg^{2+}	[3, 4]

Table G. Parameter values of presynaptic channel model.

Parameter	Value	Unit	Description	Refs
$C_{\mathrm{m,pre}}$	1.5	$\frac{\mu F}{cm^2}$	Presynaptic membrane capacitance per unit area	[2]
$g_{\mathrm{CaNHVA,pre}}$	0.3	$\frac{mS}{cm^2}$	Maximum conductance of presynaptic Ca_{NHVA} current per unit area	[1]
$g_{\mathrm{K,pre}}$	20	$\frac{mS}{cm^2}$	Maximum conductance of presynaptic K^+ current per unit area	[2]: 120 $\frac{mS}{cm^2}$
$g_{\mathrm{K,2,pre}}$	20	$\frac{mS}{cm^2}$	Maximum conductance of presynaptic K^+ current per unit area	[2]: 120 $\frac{mS}{cm^2}$
$g_{\mathrm{L,pre}}$	0.2	$\frac{mS}{cm^2}$	Leak conductance of presynaptic neuron per unit area	[2]: 0.1 $\frac{mS}{cm^2}$
$g_{\mathrm{Na,pre}}$	30	$\frac{mS}{cm^2}$	Maximum conductance of presynaptic Na^+ current per unit area	[2]: 200 $\frac{mS}{cm^2}$
$g_{\mathrm{NMDAR,pre}}$	0.1	$\frac{mS}{cm^2}$	Maximum conductance of presynaptic NMDAR per unit area	[49]: 0.014 $\frac{mS}{cm^2}$
$\tau_{h,\mathrm{CaNHVA,pre}}$	80	ms	Time constant for presynaptic Ca_{NHVA} inactivation	[1]
$\tau_{h,\mathrm{Na,pre}}$	1	ms	Time constant for presynaptic Na^+ inactivation	[2]: 0.5 ms
$\tau_{m,\mathrm{Na,pre}}$	0.05	ms	Time constant for presynaptic Na^+ activation	[2]
$\tau_{n,\mathrm{K,pre}}$	1	ms	Time constant for presynaptic K^+ activation	[2]
$\tau_{n,\mathrm{K,2,pre}}$	10	ms	Time constant for presynaptic K^+ activation	[2]
$\tau_{s,\mathrm{Na,pre}}$	30	ms	Time constant for presynaptic Na^+ inactivation	[2]
$\tau_{sb,\mathrm{Na,pre}}$	0.1	ms	Time constant for presynaptic Na^+ inactivation	[2]: 0.5 ms
$V_{\mathrm{K,pre}}$	−77	mV	Reversal potential of presynaptic K^+ current	[2]
$V_{\mathrm{L,pre}}$	−60	mV	Leak reversal potential of presynaptic neuron	[2]: −70 mV
$V_{\mathrm{Na,pre}}$	50	mV	Reversal potential of presynaptic Na^+ current	[2]: 40 mV, 70 mV
$V_{\mathrm{NMDAR,pre}}$	0	mV	Reversal potential of presynaptic NMDAR current	[16]
$V_{sd,\mathrm{Na,pre}}$	1	mV	Shift in presynaptic membrane potential used in calculation of Na^+ current	[2]
$V_{\mathrm{shift,pre}}$	−10	mV	Shift in presynaptic membrane potential	[2]: 0 mV
$V_{sv,\mathrm{Na,pre}}$	10	mV	Shift in presynaptic membrane potential used in calculation of Na^+ current	[2]: 30 mV

Table H. Parameter values of presynaptic Ca^{2+} model.

Parameter	Value	Unit	Description	Refs
$a_{0m,\mathrm{pre}}$	0.03	$\frac{1}{\mathrm{ms}}$	Parameter used in calculation of presynaptic $\mathrm{Ca_{NHVA}}$ activation time constant	[1]
$c_{\mathrm{Ca,pre}}$	$\dfrac{zFd_{\mathrm{pre}}}{k_{\mathrm{Ca,pre}}}$	$\frac{\mathrm{\mu A\ ms}}{\mathrm{cm^2 \mu M}}$	Presynaptic scaling factor to convert from units $\frac{\mathrm{\mu A}}{\mathrm{cm^2}}$ to $\frac{\mathrm{\mu M}}{\mathrm{ms}}$	[1]
$c_{V,\mathrm{pre}}$	$\dfrac{k_{V,\mathrm{pre}}R(T_{\mathrm{celsius}}+273.15)}{zF}$	mV	Presynaptic scaling factor to convert from units mV to 1 used in calculation of Goldman-Hodgkin-Katz current equation	[1]
$\mathrm{Ca_{ext,pre}}$	2,000	µM	Ca^{2+} concentration outside presynaptic neuron	[1]
$\mathrm{Ca_{rest,pre}}$	0.05	µM	Presynaptic resting Ca^{2+} concentration	[1]
d_{pre}	0.1	µm	Depth of presynaptic axonal shell	[1]
$g_{mm,\mathrm{pre}}$	0.1	1	Parameter used in calculation of presynaptic $\mathrm{Ca_{NHVA}}$ activation time constant	[1]
$k_{\mathrm{Ca,pre}}$	10,000	1	Presynaptic scaling factor used in calculation of $c_{\mathrm{Ca,pre}}$	[1]
$k_{V,\mathrm{pre}}$	1,000	1	Presynaptic scaling factor used in calculation of $c_{V,\mathrm{pre}}$	[1]
$K_{\mathrm{inh,pre}}$	1	µM	Presynaptic $\mathrm{Ca_{NHVA}}$ channel dissociation constant (inhibition)	[1]
$\tau_{\mathrm{Ca,pre}}$	100	ms	Time constant of presynaptic pump	[1]
$\tau_{m,\mathrm{min,pre}}$	0.2	ms	Minimun value for presynaptic $\mathrm{Ca_{NHVA}}$ activation time constant	[1]
$V_{1/2m,\mathrm{pre}}$	-14	mV	Half-activation potential of presynaptic $\mathrm{Ca_{NHVA}}$ channel	[1]
$z_{m,\mathrm{pre}}$	2	1	Parameter used in calculation of presynaptic $\mathrm{Ca_{NHVA}}$ activation time constant	[1]

Table I. Parameter values of presynaptic NMDAR model.

Parameter	Value	Unit	Description	Refs
$k_{\mathrm{d1,f,pre}}$	0.055	$\frac{1}{\mathrm{ms}}$	Rate constant into desensitized state of presynaptic NMDAR	[3]: 0.55 $\frac{1}{\mathrm{ms}}$
$k_{\mathrm{d1,b,pre}}$	0.0814	$\frac{1}{\mathrm{ms}}$	Rate constant out of desensitized state of presynaptic NMDAR	[3]
$k_{\mathrm{d2,f,pre}}$	0.0112	$\frac{1}{\mathrm{ms}}$	Rate constant into desensitized state of presynaptic NMDAR	[3]: 0.112 $\frac{1}{\mathrm{ms}}$
$k_{\mathrm{d2,b,pre}}$	0.00091	$\frac{1}{\mathrm{ms}}$	Rate constant out of desensitized state of presynaptic NMDAR	[3]
$k_{\mathrm{f,f,pre}}$	2.836	$\frac{1}{\mathrm{ms}}$	Rate constant for gating-associated conformational changes of presynaptic GluN1 subunit of NMDAR	[3, 4]
$k_{\mathrm{f,b,pre}}$	0.175	$\frac{1}{\mathrm{ms}}$	Rate constant for gating-associated conformational changes of presynaptic GluN1 subunit of NMDAR	[3, 4]
$k_{\mathrm{on,pre}}$	0.00283	$\frac{1}{\mathrm{\mu M\ ms}}$	Rate constant for agonist (Glu) binding to presynaptic NMDAR	[3, 4]
$k_{\mathrm{off,pre}}$	0.0381	$\frac{1}{\mathrm{ms}}$	Rate constant for agonist (Glu) unbinding from presynaptic NMDAR	[3, 4]
$k_{\mathrm{s,f,0,pre}}$	0.048	$\frac{1}{\mathrm{ms}}$	Rate constant for gating-associated conformational changes of presynaptic GluN2 subunit of NMDAR	[3, 4]
$k_{\mathrm{s,b,pre}}$	0.23	$\frac{1}{\mathrm{ms}}$	Rate constant for gating-associated conformational changes of presynaptic GluN2 subunit of NMDAR	[3, 4]

Table J. Parameter values of presynaptic Glu release model.

Parameter	Value	Unit	Description	Refs
$C_{\text{thr,pre}}$	3	µM	Ca^{2+} threshold concentration of Glu release in presynaptic neuron	
$\text{CaN}_{\text{max,pre}}$	2	µM	Total concentration of presynaptic CaN	[50]
$f_{\text{Glu,pre}}$	0.1	1	Factor representing spillover of Glu from synaptic cleft that presynaptic NMDAR receives	
G_{pre}	1,092	1	Number of Glu per presynaptic vesicle	[51]
$k_{1,\text{pre}}$	0.001	$\frac{1}{\text{µM}^3\text{ms}}$	Rate constant for Ca^{2+} activation of presynaptic CaN	[50]
$k_{2,\text{pre}}$	0.002	$\frac{1}{\text{ms}}$	Rate constant for inactivation of presynaptic CaN	[50]: 0.012 $\frac{1}{\text{ms}}$
$k_{\text{f,pre}}$	0.0075	$\frac{1}{\text{ms}}$	Presynaptic facilitation rate constant	
$k_{\text{Glu,pre}}$	10^{-6}	1	Presynaptic scaling factor to convert from units M to µM	
$k_{\text{recov,pre}}$	0.0075	$\frac{1}{\text{ms}}$	Presynaptic recovery rate constant from empty to releasable state	[8]
$K_{\text{A,pre}}$	2	µM	Presynaptic CaN concentration producing half occupation	
$K_{\text{rel,pre}}$	5	µM	Presynaptic Ca^{2+} concentration producing half occupation used in calculation of Glu release	[8]: 20 µM
$n_{1,\text{pre}}$	2	1	Presynaptic Hill coefficient	[8]: 4
$n_{2,\text{pre}}$	2	1	Presynaptic Hill coefficient	
N_{pre}	2	1	Number of readily releasable presynaptic vesicles	[52]: 4.6 ± 3.0
$p_{1,\text{pre}}$	3×10^{-5}	$\frac{1}{\text{ms}}$	Rate constant for presynaptic protein activation affecting vesicular release	
V_{syncleft}	2×10^{-18}	l	Volume of synaptic cleft	[53]: 0.7×10^{-18} l – 8×10^{-18} l, [52]: 0.76×10^{-18} l
$X_{\text{total,pre}}$	0.1	µM	Total concentration of presynaptic protein affecting vesicular release	

Table K. Initial values of presynaptic variables.

Variable	Initial value	Unit	Description	Refs
$[Ca^{2+}]_{CaNHVA,pre}$	0.082523	µM	Presynaptic Ca_{NHVA}-mediated Ca^{2+} concentration	
$[Ca^{2+}]_{NMDAR,pre}$	0.05	µM	Presynaptic NMDAR-mediated Ca^{2+} concentration	
$[CaN]_{pre}$	1.2499×10^{-4}	µM	Presynaptic CaN concentration	
$[Glu]_{syncleft}$	0	µM	Concentration of Glu in synaptic cleft	
$h_{CaNHVA,pre}$	$h_{\infty,CaNHVA,pre}$	1	Gating variable for presynaptic Ca_{NHVA} inactivation	[1]
$h_{Na,pre}$	$h_{\infty,Na,pre}$	1	Gating variable for presynaptic Na^+ inactivation	[2]
$m_{CaNHVA,pre}$	$m_{\infty,CaNHVA,pre}$	1	Gating variable for presynaptic Ca_{NHVA} activation	[1]
$m_{Na,pre}$	$m_{\infty,Na,pre}$	1	Gating variable for presynaptic Na^+ activation	[2]
$n_{K,pre}$	$n_{\infty,K,pre}$	1	Gating variable for presynaptic K^+ activation	[2]
$n_{K,2,pre}$	$n_{\infty,K,2,pre}$	1	Gating variable for presynaptic K^+ activation	[2]
$P_{rel,pre}$	0	1	Release probability of presynaptic Glu vesicles	
R_{pre}	0	1	Fraction of presynaptic NMDARs in closed state	
R_A_{pre}	0	1	Fraction of presynaptic NMDARs in single liganded state	
$R\_A_{2,pre}$	0	1	Fraction of presynaptic NMDARs in fully liganded state	
$R\_A_{2,d1,pre}$	0	1	Fraction of presynaptic NMDARs in desensitized state	
$R\_A_{2,d2,pre}$	0	1	Fraction of presynaptic NMDARs in desensitized state	
$R\_A_{2,f,pre}$	0	1	Fraction of presynaptic NMDARs in faster conformational change of GluN1 subunit	
$R\_A_{2,s,pre}$	0	1	Fraction of presynaptic NMDARs in slower conformational change of GluN2 subunit	
$R\_A_{2,O,pre}$	0	1	Fraction of presynaptic NMDARs in open state	
$R\_A\_Mg_{pre}$	0	1	Fraction of presynaptic NMDARs in single liganded Mg^{2+}-blocked state	
$R\_A_2\_Mg_{pre}$	0	1	Fraction of presynaptic NMDARs in fully liganded Mg^{2+}-blocked state	
$R\_A_{2,d1}\_Mg_{pre}$	0	1	Fraction of presynaptic NMDARs in desensitized Mg^{2+}-blocked state	
$R\_A_{2,d2}\_Mg_{pre}$	0	1	Fraction of presynaptic NMDARs in desensitized Mg^{2+}-blocked state	
$R\_A_{2,f}\_Mg_{pre}$	0	1	Fraction of presynaptic NMDARs in faster conformational change of Mg^{2+}-blocked GluN1 subunit	
$R\_A_{2,s}\_Mg_{pre}$	0	1	Fraction of presynaptic NMDARs in slower conformational change of Mg^{2+}-blocked GluN2 subunit	
$R\_A_{2,O}\_Mg_{pre}$	0	1	Fraction of presynaptic NMDARs in Mg^{2+}-blocked state	
R_Mg_{pre}	1	1	Fraction of presynaptic NMDARs in closed Mg^{2+}-blocked state	
$R_{rel,pre}$	1	1	Fraction of releasable presynaptic vesicles	
$s_{Na,pre}$	$s_{\infty,Na,pre}$	1	Gating variable for presynaptic Na^+ inactivation	[2]
V_{pre}	−59.9969	mV	Presynaptic membrane potential	
$[X]_{ac,pre}$	0	µM	Concentration of presynaptic active protein affecting vesicular release	

Table L. Intermediate variables of postsynaptic channel model.

Intermediate variable	Description	Refs
$h_{\infty,\mathrm{Na,soma,post}} = \dfrac{1}{1+\exp\left(\frac{V_{\mathrm{soma,post}}+23}{11.5}\right)}$	Steady-state value of postsynaptic Na^+ inactivation gating variable in the soma	[14]
$m_{\infty,\mathrm{Na,soma,post}} = \dfrac{1}{1+\exp\left(-\frac{V_{\mathrm{soma,post}}+17}{11}\right)}$	Steady-state value of postsynaptic Na^+ activation gating variable in the soma	[14]
$m_{\infty,\mathrm{KDR,soma,post}} = \dfrac{1}{1+\exp\left(-\frac{V_{\mathrm{soma,post}}+17}{13.6}\right)}$	Steady-state value of postsynaptic K_{DR} activation gating variable in the soma	[14]
$n_{\infty,\mathrm{NaP,soma,post}} = \dfrac{1}{1+\exp\left(-\frac{V_{\mathrm{soma,post}}+50}{6}\right)}$	Steady-state value of postsynaptic Na_P activation gating variable in the soma	[14]
$h_{\infty,\mathrm{KA,dend,post}} = \dfrac{1}{1+\exp\left(\frac{V_{\mathrm{dend,post}}+49}{6}\right)}$	Steady-state value of postsynaptic K_A inactivation gating variable in the dendrite	[14]
$m_{\infty,\mathrm{KA,dend,post}} = \dfrac{1}{1+\exp\left(-\frac{V_{\mathrm{dend,post}}+40}{8.5}\right)}$	Steady-state value of postsynaptic K_A activation gating variable in the dendrite	[14]
$h_{\infty,\mathrm{Na,dend,post}} = \dfrac{1}{1+\exp\left(\frac{V_{\mathrm{dend,post}}+23}{11.5}\right)}$	Steady-state value of postsynaptic Na^+ inactivation gating variable in the dendrite	[14]
$m_{\infty,\mathrm{Na,dend,post}} = \dfrac{1}{1+\exp\left(-\frac{V_{\mathrm{dend,post}}+17}{11}\right)}$	Steady-state value of postsynaptic Na^+ activation gating variable in the dendrite	[14]
$\alpha_{h,\mathrm{CaLHVA,post}} = 0.000457\exp\left(-\dfrac{V_{\mathrm{dend,post}}+13}{50}\right)$	Transition rate of postsynaptic Ca_{LHVA} channel's inactivation gate from non-permissive to permissive state	[11, 12]
$\alpha_{m,\mathrm{CaLHVA,post}} = -0.055\dfrac{V_{\mathrm{dend,post}}+27}{\exp\left(-\frac{V_{\mathrm{dend,post}}+27}{3.8}\right)-1}$	Transition rate of postsynaptic Ca_{LHVA} channel's activation gate from non-permissive to permissive state	[11, 12]
$\beta_{h,\mathrm{CaLHVA,post}} = \dfrac{0.0065}{1+\exp\left(-\frac{V_{\mathrm{dend,post}}+15}{28}\right)}$	Transition rate of postsynaptic Ca_{LHVA} channel's inactivation gate from permissive to non-permissive state	[11, 12]
$\beta_{m,\mathrm{CaLHVA,post}} = 0.94\exp\left(-\dfrac{V_{\mathrm{dend,post}}+75}{17}\right)$	Transition rate of postsynaptic Ca_{LHVA} channel's activation gate from permissive to non-permissive state	[11, 12]
$h_{\infty,\mathrm{CaLHVA,dend,post}} = \dfrac{\alpha_{h,\mathrm{CaLHVA,post}}}{\alpha_{h,\mathrm{CaLHVA,post}}+\beta_{h,\mathrm{CaLHVA,post}}}$	Steady-state value of postsynaptic Ca_{LHVA} inactivation gating variable in the dendrite	[11, 12]
$m_{\infty,\mathrm{CaLHVA,dend,post}} = \dfrac{\alpha_{m,\mathrm{CaLHVA,post}}}{\alpha_{m,\mathrm{CaLHVA,post}}+\beta_{m,\mathrm{CaLHVA,post}}}$	Steady-state value of postsynaptic Ca_{LHVA} activation gating variable in the dendrite	[11, 12]
$h_{\infty,\mathrm{CaLLVA,dend,post}} = \dfrac{1}{1+\exp\left(\frac{V_{\mathrm{dend,post}}+10+80}{6.4}\right)}$	Steady-state value of postsynaptic Ca_{LLVA} inactivation gating variable in the dendrite	[13]
$m_{\infty,\mathrm{CaLLVA,dend,post}} = \dfrac{1}{1+\exp\left(-\frac{V_{\mathrm{dend,post}}+10+30}{6}\right)}$	Steady-state value of postsynaptic Ca_{LLVA} activation gating variable in the dendrite	[13]
$h_{\infty,\mathrm{IP3R,post}} = \dfrac{K_{\mathrm{inh,post}}}{K_{\mathrm{inh,post}}+[\mathrm{Ca}^{2+}]_{\mathrm{post}}}$	Steady-state value of postsynaptic IP_3R inactivation gating variable	[18–20]
$m_{\infty,\mathrm{IP3R,post}} = \dfrac{[\mathrm{IP}_3]_{\mathrm{post}}}{K_{\mathrm{IP3,post}}+[\mathrm{IP}_3]_{\mathrm{post}}}$	Steady-state value of postsynaptic IP_3R activation gating variable	[18–20]
$n_{\infty,\mathrm{IP3R,post}} = \dfrac{[\mathrm{Ca}^{2+}]_{\mathrm{post}}}{K_{\mathrm{act,post}}+[\mathrm{Ca}^{2+}]_{\mathrm{post}}}$	Steady-state value of postsynaptic IP_3R activation gating variable	[18–20]

Table M. Intermediate variables of postsynaptic channel model in the dendrite.

Intermediate variable	Description	Refs
$B_{\text{NMDAR,post}} = \dfrac{1}{1 + \frac{\text{Mg}_{\text{ext,post}}}{3{,}570}\exp(-0.062 V_{\text{dend,post}})}$	Mg^{2+} block of postsynaptic NMDAR	[16]
$\tau_{h,\text{KA,post}} = \begin{cases} \frac{1}{\exp\left(\frac{V_{\text{dend,post}}+46}{5}\right)+\exp\left(-\frac{V_{\text{dend,post}}+238}{37}\right)} \frac{1}{3^{\left(\frac{T_{\text{celsius}}-23.5}{10}\right)}}, & \text{if } V_{\text{dend,post}} < -63 \text{ mV} \\ \frac{19}{3^{\frac{T_{\text{celsius}}-23.5}{10}}}, & \text{otherwise} \end{cases}$	Time constant for postsynaptic K_A inactivation	[14, 54]
$\tau_{m,\text{KA,post}} = \left(\dfrac{1}{\exp\left(\frac{V_{\text{dend,post}}+36}{20}\right) + \exp\left(-\frac{V_{\text{dend,post}}+80}{13}\right)} + 0.37\right) \dfrac{1}{3^{\left(\frac{T_{\text{celsius}}-23.5}{10}\right)}}$	Time constant for postsynaptic K_A activation	[14]
$\tau_{h,\text{CaLHVA,post}} = \dfrac{1}{\alpha_{h,\text{CaLHVA,post}} + \beta_{h,\text{CaLHVA,post}}}$	Time constant for postsynaptic Ca_{LHVA} inactivation	[11, 12]
$\tau_{m,\text{CaLHVA,post}} = \dfrac{1}{\alpha_{m,\text{CaLHVA,post}} + \beta_{m,\text{CaLHVA,post}}}$	Time constant for postsynaptic Ca_{LHVA} activation	[11, 12]
$\tau_{h,\text{CaLLVA,post}} = \left(20 + \dfrac{50}{1+\exp\left(\frac{V_{\text{dend,post}}+10+40}{7}\right)}\right) \dfrac{1}{2.3^{\frac{T_{\text{celsius}}-21}{10}}}$	Time constant for postsynaptic Ca_{LLVA} inactivation	[13]
$\tau_{m,\text{CaLLVA,post}} = \left(5 + \dfrac{20}{1+\exp\left(\frac{V_{\text{dend,post}}+10+25}{5}\right)}\right) \dfrac{1}{2.3^{\frac{T_{\text{celsius}}-21}{10}}}$	Time constant for postsynaptic Ca_{LLVA} activation	[13]

Table N. Postsynaptic channel and receptor currents.

Intermediate variable	Description	Refs
$I_{\mathrm{KDR,soma,post}} = g_{\mathrm{KDR,soma,post}} m^2_{\mathrm{KDR,soma,post}} \left(V_{\mathrm{soma,post}} - V_{\mathrm{K,post}}\right)$	Postsynaptic $\mathrm{K_{DR}}$ current density in the soma	[14]
$I_{\mathrm{L,soma,post}} = g_{\mathrm{L,soma,post}} \left(V_{\mathrm{soma,post}} - V_{\mathrm{L,post}}\right)$	Postsynaptic leak current density in the soma	[14]
$I_{\mathrm{Na,soma,post}} = g_{\mathrm{Na,soma,post}} m^2_{\mathrm{Na,soma,post}} h_{\mathrm{Na,soma,post}} \left(V_{\mathrm{soma,post}} - V_{\mathrm{Na,soma,post}}\right)$	Postsynaptic $\mathrm{Na^+}$ current density in the soma	[14]
$I_{\mathrm{NaP,soma,post}} = g_{\mathrm{NaP,soma,post}} n_{\infty,\mathrm{NaP,soma,post}} \left(V_{\mathrm{soma,post}} - V_{\mathrm{Na,soma,post}}\right)$	Postsynaptic $\mathrm{Na_P}$ current density in the soma	[14]
$I_{\mathrm{KA,dend,post}} = g_{\mathrm{KA,dend,post}} m^4_{\mathrm{KA,dend,post}} h_{\mathrm{KA,dend,post}} \left(V_{\mathrm{dend,post}} - V_{\mathrm{K,post}}\right)$	Postsynaptic $\mathrm{K_A}$ current density in the dendrite	[14]
$I_{\mathrm{CaLHVA,dend,post}} = g_{\mathrm{CaLHVA,dend,post}} m^2_{\mathrm{CaLHVA,dend,post}} h_{\mathrm{CaLHVA,dend,post}} \left(V_{\mathrm{dend,post}} - V_{\mathrm{Ca,post}}\right)$	Postsynaptic $\mathrm{Ca_{LHVA}}$ current density in the dendrite	[11, 12]
$I_{\mathrm{CaLLVA,dend,post}} = g_{\mathrm{CaLLVA,dend,post}} m^2_{\mathrm{CaLLVA,dend,post}} h_{\mathrm{CaLLVA,dend,post}} \left(V_{\mathrm{dend,post}} - V_{\mathrm{Ca,post}}\right)$	Postsynaptic $\mathrm{Ca_{LLVA}}$ current density in the dendrite	[13]
$I_{\mathrm{L,dend,post}} = g_{\mathrm{L,dend,post}} \left(V_{\mathrm{dend,post}} - V_{\mathrm{L,post}}\right)$	Postsynaptic leak current density in the dendrite	[14]
$I_{\mathrm{Na,dend,post}} = g_{\mathrm{Na,dend,post}} m^2_{\mathrm{Na,dend,post}} h_{\mathrm{Na,dend,post}} \left(V_{\mathrm{dend,post}} - V_{\mathrm{Na,dend,post}}\right)$	Postsynaptic $\mathrm{Na^+}$ current density in the dendrite	[14]
$I_{\mathrm{AMPAR,post}} = g_{\mathrm{AMPAR,post}} m_{\mathrm{AMPAR,post}} \left(V_{\mathrm{dend,post}} - V_{\mathrm{AMPAR,post}}\right)$	Postsynaptic AMPAR current density in the dendrite	[16]
$I_{\mathrm{Ca,NMDAR,post}} = g_{\mathrm{NMDAR,post}} B_{\mathrm{NMDAR,post}} m_{\mathrm{NMDAR,post}} \left(V_{\mathrm{dend,post}} - V_{\mathrm{NMDAR,post}}\right)$	$\mathrm{Ca^{2+}}$ current density via postsynaptic NMDARs in the dendrite	[16]
$I_{\mathrm{coupl,dend,post}} = \frac{g_{\mathrm{c,post}}}{1 - p_{\mathrm{post}}} \left(V_{\mathrm{soma,post}} - V_{\mathrm{dend,post}}\right)$	Postsynaptic current coupling term in the dendrite	[14]
$I_{\mathrm{coupl,soma,post}} = \frac{g_{\mathrm{c,post}}}{p_{\mathrm{post}}} \left(V_{\mathrm{dend,post}} - V_{\mathrm{soma,post}}\right)$	Postsynaptic current coupling term in the soma	[14]

Table O. Intermediate variables of postsynaptic flux model.

**Intermediate variable**	**Description**	**Refs**
$J_{\mathrm{CaL,post}} = -\frac{I_{\mathrm{CaLHVA,dend,post}} + I_{\mathrm{CaLLVA,dend,post}}}{c_{\mathrm{Ca,post}}}$	Ca^{2+} flux via postsynaptic Ca_{LHVA} and Ca_{LLVA} channels	
$J_{\mathrm{IP3R,post}} = v_{\mathrm{IP3R,post}} m^3_{\infty,\mathrm{IP3R,post}} n^3_{\infty,\mathrm{IP3R,post}} h^3_{\mathrm{IP3R,post}} \left([\mathrm{Ca}^{2+}]_{\mathrm{ER,post}} - [\mathrm{Ca}^{2+}]_{\mathrm{post}}\right)$	Ca^{2+} flux via postsynaptic IP_3R	[18–20]
$J_{\mathrm{leakER,post}} = r_{\mathrm{leakER,post}} \left([\mathrm{Ca}^{2+}]_{\mathrm{ER,post}} - [\mathrm{Ca}^{2+}]_{\mathrm{post}}\right)$	Postsynaptic Ca^{2+} leak flux from ER to cytosol	[18, 19]
$J_{\mathrm{leakCell,post}} = r_{\mathrm{leakCell,post}} \left(\mathrm{Ca}_{\mathrm{ext,post}} - [\mathrm{Ca}^{2+}]_{\mathrm{post}}\right)$	Postsynaptic Ca^{2+} leak flux from extracellular space to cytosol	[21]
$J_{\mathrm{NMDAR,post}} = -\frac{I_{\mathrm{Ca,NMDAR,post}}}{c_{\mathrm{Ca,post}}}$	Ca^{2+} flux via postsynaptic NMDAR	
$J_{\mathrm{PMCA,post}} = \frac{k_{\mathrm{Ca,post}} A_{\mathrm{spine,post}} v_{\mathrm{PMCA,post}}}{V_{\mathrm{spine,post}}} \frac{[\mathrm{Ca}^{2+}]^2_{\mathrm{post}}}{K^2_{\mathrm{PMCA,post}} + [\mathrm{Ca}^{2+}]^2_{\mathrm{post}}}$	Ca^{2+} flux via postsynaptic PMCA pump	[21]
$J_{\mathrm{SERCA,post}} = v_{\mathrm{SERCA,post}} \frac{[\mathrm{Ca}^{2+}]^2_{\mathrm{post}}}{K^2_{\mathrm{SERCA,post}} + [\mathrm{Ca}^{2+}]^2_{\mathrm{post}}}$	Ca^{2+} flux via postsynaptic SERCA pump	[18, 19]

Table P. Reactions of postsynaptic neuron model.

Reaction	Description	Refs
$Glu_{syncleft} \rightarrow \emptyset$	Postsynaptic mGluR Glu uptake	[17]
$Glu_{syncleft} + mGluR \rightleftharpoons Glu\_mGluR$	Postsynaptic mGluR Glu binding	[17]
$Glu\_mGluR \rightleftharpoons Glu\_mGluRdesens$	Postsynaptic mGluR desensitization	[17]
$G\alpha\beta\gamma + Glu\_mGluR \rightleftharpoons G\alpha\beta\gamma\_Glu\_mGluR \rightarrow Glu\_mGluR + G\alpha GTP$	Postsynaptic G protein activation	[17]
$Ca^{2+} + PLC \rightleftharpoons Ca\_PLC$	Postsynaptic PLC binding Ca^{2+} first	[17]
$G\alpha GTP + Ca\_PLC \rightleftharpoons Ca\_G\alpha GTP\_PLC$	Postsynaptic PLC binding GαGTP second	[17]
$G\alpha GTP + PLC \rightleftharpoons G\alpha GTP\_PLC$	Postsynaptic PLC binding GαGTP first	[17]
$Ca^{2+} + G\alpha GTP\_PLC \rightleftharpoons Ca\_G\alpha GTP\_PLC$	Postsynaptic PLC binding Ca^{2+} second	[17]
$PIP_2 + Ca\_PLC \rightleftharpoons Ca\_PIP_2\_PLC \rightarrow Ca\_DAG\_PLC + IP_3$	Postsynaptic DAG production, step 1	[17]
$Ca\_DAG\_PLC \rightarrow Ca\_PLC + DAG$	Postsynaptic DAG production, step 2	[17]
$PIP_2 + Ca\_G\alpha GTP\_PLC \rightleftharpoons Ca\_G\alpha GTP\_PIP_2\_PLC \rightarrow Ca\_DAG\_G\alpha GTP\_PLC + IP_3$	Postsynaptic DAG production, step 1	[17]
$Ca\_DAG\_G\alpha GTP\_PLC \rightarrow Ca\_G\alpha GTP\_PLC + DAG$	Postsynaptic DAG production, step 2	[17]
$IP_3 \rightarrow IP_3deg$	Postsynaptic IP_3 degradation	[17]
$IP_3deg + PIKin \rightleftharpoons IP_3deg\_PIKin \rightarrow PIP_2 + PIKin$	Postsynaptic PIP_2 regeneration by PIKin	[17]
$G\alpha GTP\_PLC \rightarrow PLC + G\alpha GDP$	Postsynaptic GAP activity on PLC	[17]
$Ca\_G\alpha GTP\_PLC \rightarrow Ca\_PLC + G\alpha GDP$	Postsynaptic GAP activity on PLC	[17]
$G\alpha GTP \rightarrow G\alpha GDP$	Postsynaptic GαGTP hydrolysis	[17]
$G\alpha GDP \rightarrow G\alpha\beta\gamma$	Postsynaptic G protein regeneration	[17]
$Ca^{2+} + DAGL \rightleftharpoons Ca\_DAGL$	Postsynaptic Ca^{2+} activating DAGL	[17]
$DAG + Ca\_DAGL \rightleftharpoons Ca\_DAG\_DAGL \rightarrow Ca\_DAGL + 2\text{-}AG$	Postsynaptic 2-AG production	[17]
$2\text{-}AG \rightarrow \emptyset$	Postsynaptic 2-AG degradation	[17]
$DAG \rightarrow \emptyset$	Postsynaptic DAG degradation	[22]

Table Q. Reaction rates of postsynaptic model.

Reaction rate	Description	Refs
$v_{\mathrm{Glu,f,post}} = k_{\mathrm{Glu,f,post}}\,(1-f_{\mathrm{Glu,pre}})\,[\mathrm{Glu}]_{\mathrm{syncleft}}$	Reaction rate for postsynaptic mGluR Glu uptake	Mod. from [17]
$v_{\mathrm{mGluR,f,post}} = k_{\mathrm{mGluR,f,post}}\,(1-f_{\mathrm{Glu,pre}})\,[\mathrm{Glu}]_{\mathrm{syncleft}}[\mathrm{mGluR}]_{\mathrm{post}}$<br>$v_{\mathrm{mGluR,b,post}} = k_{\mathrm{mGluR,b,post}}[\mathrm{Glu\_mGluR}]_{\mathrm{post}}$	Reaction rates of Glu binding and unbinding to postsynaptic mGluR	Mod. from [17]
$v_{\mathrm{mGluRdes,f,post}} = k_{\mathrm{mGluRdes,f,post}}[\mathrm{Glu\_mGluR}]_{\mathrm{post}}$<br>$v_{\mathrm{mGluRdes,b,post}} = k_{\mathrm{mGluRdes,b,post}}[\mathrm{Glu\_mGluRdesens}]_{\mathrm{post}}$	Reaction rates into and out of desensitized state of postsynaptic mGluR	[17]
$v_{\mathrm{Gact,f,post}} = k_{\mathrm{Gact,f,post}}[\mathrm{G}\alpha\beta\gamma]_{\mathrm{post}}[\mathrm{Glu\_mGluR}]_{\mathrm{post}}$<br>$v_{\mathrm{Gact,b,post}} = k_{\mathrm{Gact,b,post}}[\mathrm{G}\alpha\beta\gamma\mathrm{\_Glu\_mGluR}]_{\mathrm{post}}$<br>$v_{\mathrm{Gact,c,post}} = k_{\mathrm{Gact,c,post}}[\mathrm{G}\alpha\beta\gamma\mathrm{\_Glu\_mGluR}]_{\mathrm{post}}$	Reaction rates of postsynaptic G protein activation and inactivation	[17]
$v_{\mathrm{Ca\_PLC1,f,post}} = k_{\mathrm{Ca\_PLC1,f,post}}[\mathrm{Ca}^{2+}]_{\mathrm{post}}[\mathrm{PLC}]_{\mathrm{post}}$<br>$v_{\mathrm{Ca\_PLC1,b,post}} = k_{\mathrm{Ca\_PLC1,b,post}}[\mathrm{Ca\_PLC}]_{\mathrm{post}}$	Reaction rates of postsynaptic PLC binding and unbinding Ca^{2+} first	[17]
$v_{\mathrm{G\_PLC2,f,post}} = k_{\mathrm{G\_PLC2,f,post}}[\mathrm{G}\alpha\mathrm{GTP}]_{\mathrm{post}}[\mathrm{Ca\_PLC}]_{\mathrm{post}}$<br>$v_{\mathrm{G\_PLC2,b,post}} = k_{\mathrm{G\_PLC2,b,post}}[\mathrm{Ca\_G}\alpha\mathrm{GTP\_PLC}]_{\mathrm{post}}$	Reaction rates of postsynaptic PLC binding and unbinding GαGTP second	[17]
$v_{\mathrm{G\_PLC1,f,post}} = k_{\mathrm{G\_PLC1,f,post}}[\mathrm{G}\alpha\mathrm{GTP}]_{\mathrm{post}}[\mathrm{PLC}]_{\mathrm{post}}$<br>$v_{\mathrm{G\_PLC1,b,post}} = k_{\mathrm{G\_PLC1,b,post}}[\mathrm{G}\alpha\mathrm{GTP\_PLC}]_{\mathrm{post}}$	Reaction rates of postsynaptic PLC binding and unbinding GαGTP first	[17]
$v_{\mathrm{Ca\_PLC2,f,post}} = k_{\mathrm{Ca\_PLC2,f,post}}[\mathrm{Ca}^{2+}]_{\mathrm{post}}[\mathrm{G}\alpha\mathrm{GTP\_PLC}]_{\mathrm{post}}$<br>$v_{\mathrm{Ca\_PLC2,b,post}} = k_{\mathrm{Ca\_PLC2,b,post}}[\mathrm{Ca\_G}\alpha\mathrm{GTP\_PLC}]_{\mathrm{post}}$	Reaction rates of postsynaptic PLC binding and unbinding Ca^{2+} second	[17]
$v_{\mathrm{DAG1,f,post}} = k_{\mathrm{DAG1,f,post}}[\mathrm{PIP_2}]_{\mathrm{post}}[\mathrm{Ca\_PLC}]_{\mathrm{post}}$<br>$v_{\mathrm{DAG1,b,post}} = k_{\mathrm{DAG1,b,post}}[\mathrm{Ca\_PIP_2\_PLC}]_{\mathrm{post}}$<br>$v_{\mathrm{DAG1,c,post}} = k_{\mathrm{DAG1,c,post}}[\mathrm{Ca\_PIP_2\_PLC}]_{\mathrm{post}}$	Reaction rates of postsynaptic DAG production, step 1	[17]
$v_{\mathrm{DAG2,f,post}} = k_{\mathrm{DAG2,f,post}}[\mathrm{Ca\_DAG\_PLC}]_{\mathrm{post}}$	Reaction rate of postsynaptic DAG production, step 2	[17]
$v_{\mathrm{DAG3,f,post}} = k_{\mathrm{DAG3,f,post}}[\mathrm{Ca\_G}\alpha\mathrm{GTP\_PLC}]_{\mathrm{post}}[\mathrm{PIP_2}]_{\mathrm{post}}$<br>$v_{\mathrm{DAG3,b,post}} = k_{\mathrm{DAG3,b,post}}[\mathrm{Ca\_G}\alpha\mathrm{GTP\_PIP_2\_PLC}]_{\mathrm{post}}$<br>$v_{\mathrm{DAG3,c,post}} = k_{\mathrm{DAG3,c,post}}[\mathrm{Ca\_G}\alpha\mathrm{GTP\_PIP_2\_PLC}]_{\mathrm{post}}$	Reaction rates of postsynaptic DAG production, step 1	[17]
$v_{\mathrm{DAG4,f,post}} = k_{\mathrm{DAG4,f,post}}[\mathrm{Ca\_DAG\_G}\alpha\mathrm{GTP\_PLC}]_{\mathrm{post}}$	Reaction rate of postsynaptic DAG production, step 2	[17]
$v_{\mathrm{degIP3,post}} = k_{\mathrm{degIP3,post}}[\mathrm{IP_3}]_{\mathrm{post}}$	Reaction rate of postsynaptic $\mathrm{IP_3}$ degradation	[17]
$v_{\mathrm{PIP2,f,post}} = k_{\mathrm{PIP2,f,post}}[\mathrm{IP_3deg}]_{\mathrm{post}}[\mathrm{PIKin}]_{\mathrm{post}}$<br>$v_{\mathrm{PIP2,b,post}} = k_{\mathrm{PIP2,b,post}}[\mathrm{IP_3deg\_PIKin}]_{\mathrm{post}}$<br>$v_{\mathrm{PIP2,c,post}} = k_{\mathrm{PIP2,c,post}}[\mathrm{IP_3deg\_PIKin}]_{\mathrm{post}}$	Reaction rates of postsynaptic $\mathrm{PIP_2}$ regeneration by PIKin	[17]
$v_{\mathrm{GAP1,f,post}} = k_{\mathrm{GAP1,f,post}}[\mathrm{G}\alpha\mathrm{GTP\_PLC}]_{\mathrm{post}}$	Reaction rate of postsynaptic GAP activity on PLC	[17]
$v_{\mathrm{GAP2,f,post}} = k_{\mathrm{GAP2,f,post}}[\mathrm{Ca\_G}\alpha\mathrm{GTP\_PLC}]_{\mathrm{post}}$	Reaction rate of postsynaptic GAP activity on PLC	[17]
$v_{\mathrm{hydrG,f,post}} = k_{\mathrm{hydrG,f,post}}[\mathrm{G}\alpha\mathrm{GTP}]_{\mathrm{post}}$	Reaction rate of postsynaptic GαGTP hydrolysis	[17]
$v_{\mathrm{regenG,f,post}} = k_{\mathrm{regenG,f,post}}[\mathrm{G}\alpha\mathrm{GDP}]_{\mathrm{post}}$	Reaction rate of postsynaptic G protein regeneration	[17]
$v_{\mathrm{DAGL,f,post}} = k_{\mathrm{DAGL,f,post}}[\mathrm{Ca}^{2+}]_{\mathrm{post}}[\mathrm{DAGL}]_{\mathrm{post}}$<br>$v_{\mathrm{DAGL,b,post}} = k_{\mathrm{DAGL,b,post}}[\mathrm{Ca\_DAGL}]_{\mathrm{post}}$	Reaction rates of postsynaptic Ca^{2+} activation of DAGL	[17]
$v_{\mathrm{prodAG,f,post}} = k_{\mathrm{prodAG,f,post}}[\mathrm{DAG}]_{\mathrm{post}}[\mathrm{Ca\_DAGL}]_{\mathrm{post}}$<br>$v_{\mathrm{prodAG,b,post}} = k_{\mathrm{prodAG,b,post}}[\mathrm{Ca\_DAG\_DAGL}]_{\mathrm{post}}$<br>$v_{\mathrm{prodAG,c,post}} = k_{\mathrm{prodAG,c,post}}[\mathrm{Ca\_DAG\_DAGL}]_{\mathrm{post}}$	Reaction rates of postsynaptic 2-AG production	[17]
$v_{\mathrm{degAG,post}} = k_{\mathrm{degAG,post}}[\mathrm{2\text{-}AG}]_{\mathrm{post}}$	Reaction rate of postsynaptic 2-AG degradation	[17]
$v_{\mathrm{degDAG,post}} = k_{\mathrm{degDAG,post}}[\mathrm{DAG}]_{\mathrm{post}}$	Reaction rate of postsynaptic DAG degradation	[22]

Table R. Parameter values of postsynaptic channel and receptor models.

Parameter	Value	Unit	Description	Refs
$\alpha_{\mathrm{AMPAR,post}}$	0.0011	$\frac{1}{\mu\mathrm{M\ ms}}$	Rate constant of opening postsynaptic AMPAR	[16]
$\alpha_{\mathrm{NMDAR,post}}$	7.2×10^{-5}	$\frac{1}{\mu\mathrm{M\ ms}}$	Rate constant of opening postsynaptic NMDAR	[16]
$\beta_{\mathrm{AMPAR,post}}$	0.19	$\frac{1}{\mathrm{ms}}$	Rate constant of closing postsynaptic AMPAR	[16]
$\beta_{\mathrm{NMDAR,post}}$	0.0066	$\frac{1}{\mathrm{ms}}$	Rate constant of closing postsynaptic NMDAR	[16]
$C_{\mathrm{m,post}}$	3	$\frac{\mu\mathrm{F}}{\mathrm{cm}^2}$	Postsynaptic membrane capacitance per unit area	[14]: 2.7 $\frac{\mu\mathrm{F}}{\mathrm{cm}^2}$
$g_{\mathrm{AMPAR,post}}$	0.1	$\frac{\mathrm{mS}}{\mathrm{cm}^2}$	Maximum conductance of postsynaptic AMPAR per unit area	[16, 55, 56]: 3.5×10^{-7} mS
$g_{\mathrm{c,post}}$	2.1	$\frac{\mathrm{mS}}{\mathrm{cm}^2}$	Coupling conductance between postsynaptic soma and dendrite per unit area	[15]
$g_{\mathrm{CaLHVA,dend,post}}$	0.23	$\frac{\mathrm{mS}}{\mathrm{cm}^2}$	Maximum conductance of postsynaptic $\mathrm{Ca_{LHVA}}$ current in the dendrite per unit area	[11, 12]: 0.01 $\frac{\mathrm{mS}}{\mathrm{cm}^2}$
$g_{\mathrm{CaLLVA,dend,post}}$	0.23	$\frac{\mathrm{mS}}{\mathrm{cm}^2}$	Maximum conductance of postsynaptic $\mathrm{Ca_{LLVA}}$ current in the dendrite per unit area	[13]: 0.01 $\frac{\mathrm{mS}}{\mathrm{cm}^2}$
$g_{\mathrm{KA,dend,post}}$	1	$\frac{\mathrm{mS}}{\mathrm{cm}^2}$	Maximum conductance of postsynaptic $\mathrm{K_A}$ current in the dendrite per unit area	Mod. from [14]
$g_{\mathrm{KDR,soma,post}}$	50	$\frac{\mathrm{mS}}{\mathrm{cm}^2}$	Maximum conductance of postsynaptic $\mathrm{K_{DR}}$ current in the soma per unit area	[14]: 154 $\frac{\mathrm{mS}}{\mathrm{cm}^2}$
$g_{\mathrm{L,dend,post}}$	0.2	$\frac{\mathrm{mS}}{\mathrm{cm}^2}$	Leak conductance of postsynaptic dendrite per unit area	[15]: 0.1 $\frac{\mathrm{mS}}{\mathrm{cm}^2}$
$g_{\mathrm{L,soma,post}}$	0.2	$\frac{\mathrm{mS}}{\mathrm{cm}^2}$	Leak conductance of postsynaptic soma per unit area	[14]: 0.222 $\frac{\mathrm{mS}}{\mathrm{cm}^2}$, [15]: 0.1 $\frac{\mathrm{mS}}{\mathrm{cm}^2}$
$g_{\mathrm{Na,dend,post}}$	0.06	$\frac{\mathrm{mS}}{\mathrm{cm}^2}$	Maximum conductance of postsynaptic $\mathrm{Na^+}$ current in the dendrite per unit area	[14]: 1.78 $\frac{\mathrm{mS}}{\mathrm{cm}^2}$
$g_{\mathrm{Na,soma,post}}$	60	$\frac{\mathrm{mS}}{\mathrm{cm}^2}$	Maximum conductance of postsynaptic $\mathrm{Na^+}$ current in the soma per unit area	[14]: 178 $\frac{\mathrm{mS}}{\mathrm{cm}^2}$
$g_{\mathrm{NaP,soma,post}}$	0.1	$\frac{\mathrm{mS}}{\mathrm{cm}^2}$	Maximum conductance of postsynaptic $\mathrm{Na_P}$ current in the soma per unit area	[14]: 0.6 $\frac{\mathrm{mS}}{\mathrm{cm}^2}$
$g_{\mathrm{NMDAR,post}}$	0.001	$\frac{\mathrm{mS}}{\mathrm{cm}^2}$	Maximum conductance of postsynaptic NMDAR per unit area	[16]: 10^{-8} mS
$\mathrm{Mg_{ext,post}}$	1,000	μM	$\mathrm{Mg^{2+}}$ concentration outside postsynaptic neuron	[16]
p_{post}	0.5	1	Proportion of postsynaptic cell area taken by soma	[15]
$\tau_{h,\mathrm{Na,post}}$	0.5	ms	Time constant for postsynaptic $\mathrm{Na^+}$ inactivation	[14]
$\tau_{m,\mathrm{KDR,post}}$	2	ms	Time constant for postsynaptic $\mathrm{K_{DR}}$ activation	[14]
$\tau_{m,\mathrm{Na,post}}$	0.05	ms	Time constant for postsynaptic $\mathrm{Na^+}$ activation	[14]
$V_{\mathrm{AMPAR,post}}$	0	mV	Reversal potential of postsynaptic AMPAR current	[16]
$V_{\mathrm{Ca,post}}$	90	mV	Reversal potential of postsynaptic $\mathrm{Ca_{LHVA}}$ and $\mathrm{Ca_{LLVA}}$ currents	[11]
$V_{\mathrm{K,post}}$	−85	mV	Reversal potential of postsynaptic $\mathrm{K^+}$ current	[14]: −77 mV
$V_{\mathrm{L,post}}$	−70	mV	Leak reversal potential of postsynaptic neuron	[14]: −73.6 mV
$V_{\mathrm{Na,dend,post}}$	50	mV	Reversal potential of postsynaptic $\mathrm{Na^+}$ current in the dendrite	[14]
$V_{\mathrm{Na,soma,post}}$	50	mV	Reversal potential of postsynaptic $\mathrm{Na^+}$ current in the soma	[14]: 90 mV
$V_{\mathrm{NMDAR,post}}$	0	mV	Reversal potential of postsynaptic NMDAR current	[16]

Table S. Parameter values of postsynaptic flux model.

Parameter	Value	Unit	Description	Refs
$A_{\text{spine,post}}$	$4\pi r^2_{\text{spine,post}}$	cm^2	Postsynaptic surface area of dendritic spine	
B_{post}	0.5	1	Postsynaptic fast buffering factor	[22]: 0.01
$c_{\text{Ca,post}}$	$\frac{zFV_{\text{spine,post}}}{B_{\text{post}}A_{\text{spine,post}}}$	$\frac{\text{µA ms}}{\text{cm}^2\text{µM}}$	Postsynaptic scaling factor to convert from units $\frac{\text{µA}}{\text{cm}^2}$ to $\frac{\text{µM}}{\text{ms}}$	[22]
$\text{Ca}_{\text{ext,post}}$	2,015.1	µM	Ca^{2+} concentration outside postsynaptic neuron	[17]
$k_{\text{Ca,post}}$	1,000	1	Postsynaptic scaling factor to convert from units $\frac{\text{mM}}{\text{ms}}$ to $\frac{\text{µM}}{\text{ms}}$	
$K_{\text{act,post}}$	0.8	µM	Postsynaptic IP_3R dissociation constant of Ca^{2+} (activation)	[20]
$K_{\text{inh,post}}$	1.9	µM	Postsynaptic IP_3R dissociation constant of Ca^{2+} (inhibition)	[20]
$K_{\text{IP3,post}}$	0.15	µM	Postsynaptic IP_3R dissociation constant of IP_3 (activation)	[20]
$K_{\text{PMCA,post}}$	0.12	µM	Half-activation constant of postsynaptic PMCA pump	[57]
$K_{\text{SERCA,post}}$	0.4	µM	Half-activation constant of postsynaptic SERCA pump	[20]
$r_{\text{ERcyt,post}}$	0.185	1	Ratio between postsynaptic ER and cytosol volumes	[18]
$r_{\text{leakCell,post}}$	$\frac{J_{\text{PMCA,post}} - J_{\text{CaL,post}} - J_{\text{NMDAR,post}}}{\text{Ca}_{\text{ext,post}} - [\text{Ca}^{2+}]_{\text{post}}}$	$\frac{1}{\text{ms}}$	Postsynaptic leak parameter over cell membrane calculated at resting concentrations	
$r_{\text{leakER,post}}$	$\frac{J_{\text{SERCA,post}} - J_{\text{IP3R,post}}}{[\text{Ca}^{2+}]_{\text{ER,post}} - [\text{Ca}^{2+}]_{\text{post}}}$	$\frac{1}{\text{ms}}$	Postsynaptic ER leak parameter calculated at resting concentrations	
$r_{\text{spine,post}}$	5×10^{-5}	cm	Postsynaptic radius of dendritic spine	[58]
$\tau_{\text{IP3R,post}}$	2,000	ms	Time constant for postsynaptic IP_3R inactivation	[20]
$v_{\text{IP3R,post}}$	0.01	$\frac{1}{\text{ms}}$	Maximum rate of Ca^{2+} release via postsynaptic IP_3R	[20]: 0.0085 $\frac{1}{\text{ms}}$
$v_{\text{PMCA,post}}$	8×10^{-11}	$\frac{\text{µMol}}{\text{mscm}^2}$	Maximum rate of Ca^{2+}uptake by postsynaptic PMCA pump per unit area	[21]: 10^{-9} $\frac{\text{µMol}}{\text{mscm}^2}$
$v_{\text{SERCA,post}}$	0.003	$\frac{\text{µM}}{\text{ms}}$	Maximum rate of Ca^{2+} uptake by postsynaptic SERCA pump	[18]: 0.0009 $\frac{\text{µM}}{\text{ms}}$
$V_{\text{spine,post}}$	$\frac{4}{3}\pi r^3_{\text{spine,post}}$	cm^3	Postsynaptic volume of dendritic spine	

Table T. Parameter values of postsynaptic reaction model.

Parameter	Value	Unit	Refs	Parameter	Value	Unit	Refs	Parameter	Value	Unit	Refs
$k_{\mathrm{Glu,f,post}}$	0.2	$\frac{1}{\mathrm{ms}}$	[17]: 0.002 $\frac{1}{\mathrm{ms}}$								
$k_{\mathrm{mGluR,f,post}}$	0.0001	$\frac{1}{\mathrm{\mu M\ ms}}$	[17]	$k_{\mathrm{mGluR,b,post}}$	0.01	$\frac{1}{\mathrm{ms}}$	[17]				
$k_{\mathrm{mGluRdes,f,post}}$	0.00025	$\frac{1}{\mathrm{ms}}$	[17]	$k_{\mathrm{mGluRdes,b,post}}$	10^{-6}	$\frac{1}{\mathrm{ms}}$	[17]				
$k_{\mathrm{Gact,f,post}}$	0.015	$\frac{1}{\mathrm{\mu M\ ms}}$	[17]	$k_{\mathrm{Gact,b,post}}$	0.0072	$\frac{1}{\mathrm{ms}}$	[17]	$k_{\mathrm{Gact,c,post}}$	0.0005	$\frac{1}{\mathrm{ms}}$	[17]
$k_{\mathrm{Ca\_PLC1,f,post}}$	0.002	$\frac{1}{\mathrm{\mu M\ ms}}$	[17]: 0.02 $\frac{1}{\mathrm{\mu M\ ms}}$	$k_{\mathrm{Ca\_PLC1,b,post}}$	0.12	$\frac{1}{\mathrm{ms}}$	[17]				
$k_{\mathrm{G\_PLC2,f,post}}$	0.1	$\frac{1}{\mathrm{\mu M\ ms}}$	[17]	$k_{\mathrm{G\_PLC2,b,post}}$	0.01	$\frac{1}{\mathrm{ms}}$	[17]				
$k_{\mathrm{G\_PLC1,f,post}}$	0.01	$\frac{1}{\mathrm{\mu M\ ms}}$	[17]	$k_{\mathrm{G\_PLC1,b,post}}$	0.012	$\frac{1}{\mathrm{ms}}$	[17]				
$k_{\mathrm{Ca\_PLC2,f,post}}$	0.08	$\frac{1}{\mathrm{\mu M\ ms}}$	[17]	$k_{\mathrm{Ca\_PLC2,b,post}}$	0.04	$\frac{1}{\mathrm{ms}}$	[17]				
$k_{\mathrm{DAG1,f,post}}$	0.0006	$\frac{1}{\mathrm{\mu M\ ms}}$	[17]: 0.006 $\frac{1}{\mathrm{\mu M\ ms}}$	$k_{\mathrm{DAG1,b,post}}$	0.01	$\frac{1}{\mathrm{ms}}$	[17]	$k_{\mathrm{DAG1,c,post}}$	0.025	$\frac{1}{\mathrm{ms}}$	[17]
$k_{\mathrm{DAG2,f,post}}$	0.2	$\frac{1}{\mathrm{ms}}$	[17]								
$k_{\mathrm{DAG3,f,post}}$	0.015	$\frac{1}{\mathrm{\mu M\ ms}}$	[17]	$k_{\mathrm{DAG3,b,post}}$	0.075	$\frac{1}{\mathrm{ms}}$	[17]	$k_{\mathrm{DAG3,c,post}}$	0.25	$\frac{1}{\mathrm{ms}}$	[17]
$k_{\mathrm{DAG4,f,post}}$	1	$\frac{1}{\mathrm{ms}}$	[17]								
$k_{\mathrm{degIP3,post}}$	0.01	$\frac{1}{\mathrm{ms}}$	[17]								
$k_{\mathrm{PIP2,f,post}}$	0.002	$\frac{1}{\mathrm{\mu M\ ms}}$	[17]	$k_{\mathrm{PIP2,b,post}}$	0.001	$\frac{1}{\mathrm{ms}}$	[17]	$k_{\mathrm{PIP2,c,post}}$	0.001	$\frac{1}{\mathrm{ms}}$	[17]
$k_{\mathrm{GAP1,f,post}}$	0.03	$\frac{1}{\mathrm{ms}}$	[17]								
$k_{\mathrm{GAP2,f,post}}$	0.03	$\frac{1}{\mathrm{ms}}$	[17]								
$k_{\mathrm{hydrG,f,post}}$	0.001	$\frac{1}{\mathrm{ms}}$	[17]								
$k_{\mathrm{regenG,f,post}}$	0.01	$\frac{1}{\mathrm{ms}}$	[17]								
$k_{\mathrm{DAGL,f,post}}$	0.125	$\frac{1}{\mathrm{\mu M\ ms}}$	[17]	$k_{\mathrm{DAGL,b,post}}$	0.05	$\frac{1}{\mathrm{ms}}$	[17]				
$k_{\mathrm{prodAG,f,post}}$	0.0025	$\frac{1}{\mathrm{\mu M\ ms}}$	[17]	$k_{\mathrm{prodAG,b,post}}$	0.0015	$\frac{1}{\mathrm{ms}}$	[17]	$k_{\mathrm{prodAG,c,post}}$	0.001	$\frac{1}{\mathrm{ms}}$	[17]
$k_{\mathrm{degAG,post}}$	0.005	$\frac{1}{\mathrm{ms}}$	[17]								
$k_{\mathrm{degDAG,post}}$	0.00066	$\frac{1}{\mathrm{ms}}$	[22, 57]								

Table U. Initial values of postsynaptic variables.

Variable	Initial value	Unit	Description	Refs
$[2\text{-AG}]_{\text{post}}$	0.0010453	µM	Postsynaptic 2-AG concentration	
$[\text{Ca}^{2+}]_{\text{post}}$	0.049978	µM	Postsynaptic Ca^{2+} concentration	
$[\text{Ca}^{2+}]_{\text{ER,post}}$	62.9016	µM	Postsynaptic Ca^{2+} concentration in ER	
$[\text{Ca\_DAG\_DAGL}]_{\text{post}}$	0.0052265	µM	Concentration of postsynaptic Ca^{2+}-DAG-DAGL complex	
$[\text{Ca\_DAG\_G}\alpha\text{GTP\_PLC}]_{\text{post}}$	0	µM	Concentration of postsynaptic Ca^{2+}-DAG-GαGTP-PLC complex	
$[\text{Ca\_DAG\_PLC}]_{\text{post}}$	8.8541×10^{-5}	µM	Concentration of postsynaptic Ca^{2+}-DAG-PLC complex	
$[\text{Ca\_DAGL}]_{\text{post}}$	0.27637	µM	Concentration of postsynaptic Ca^{2+}-DAGL complex	
$[\text{Ca\_G}\alpha\text{GTP\_PIP2\_PLC}]_{\text{post}}$	0	µM	Concentration of postsynaptic Ca^{2+}-GαGTP-PIP_2-PLC complex	
$[\text{Ca\_G}\alpha\text{GTP\_PLC}]_{\text{post}}$	0	µM	Concentration of postsynaptic Ca^{2+}-GαGTP-PLC complex	
$[\text{Ca\_PIP2\_PLC}]_{\text{post}}$	0.00070833	µM	Concentration of postsynaptic Ca^{2+}-PIP_2-PLC complex	
$[\text{Ca\_PLC}]_{\text{post}}$	0.00083161	µM	Concentration of postsynaptic Ca^{2+}-PLC complex	
$[\text{DAG}]_{\text{post}}$	0.018912	µM	Postsynaptic DAG concentration	
$[\text{DAGL}]_{\text{post}}$	2.2119	µM	Postsynaptic DAGL concentration	
$[\text{G}\alpha\beta\gamma]_{\text{post}}$	3.5	µM	Postsynaptic Gαβγ concentration	[17]
$[\text{G}\alpha\beta\gamma\text{\_Glu\_mGluR}]_{\text{post}}$	0	µM	Concentration of postsynaptic Gαβγ-Glu-mGluR complex	
$[\text{G}\alpha\text{GDP}]_{\text{post}}$	0	µM	Postsynaptic GαGDP concentration	
$[\text{G}\alpha\text{GTP}]_{\text{post}}$	0	µM	Postsynaptic GαGTP concentration	
$[\text{G}\alpha\text{GTP\_PLC}]_{\text{post}}$	0	µM	Concentration of postsynaptic GαGTP-PLC complex	
$[\text{Glu\_mGluR}]_{\text{post}}$	0	µM	Concentration of postsynaptic Glu-mGluR complex	
$[\text{Glu\_mGluRdesens}]_{\text{post}}$	0	µM	Concentration of postsynaptic Glu-mGluRdesens complex	
$[\text{IP}_3]_{\text{post}}$	0.0017708	µM	Postsynaptic IP_3 concentration	
$[\text{IP}_3\text{deg}]_{\text{post}}$	0.014141	µM	Postsynaptic IP_3deg concentration	
$[\text{IP}_3\text{deg\_PIKin}]_{\text{post}}$	0.017708	µM	Concentration of postsynaptic IP_3deg-PIKin complex	
$[\text{mGluR}]_{\text{post}}$	5	µM	Postsynaptic mGluR concentration	[17]
$[\text{PIKin}]_{\text{post}}$	1.2523	µM	Postsynaptic PIKin concentration	
$[\text{PIP}_2]_{\text{post}}$	49.6857	µM	Postsynaptic PIP_2 concentration	
$[\text{PLC}]_{\text{post}}$	0.99837	µM	Postsynaptic PLC concentration	
$V_{\text{dend,post}}$	−68.1916	mV	Postsynaptic membrane potential in the dendrite	
$V_{\text{soma,post}}$	−68.1057	mV	Postsynaptic membrane potential in the soma	

Table V. Initial values of postsynaptic variables.

Variable	Initial value	Unit	Description	Refs
$h_{\mathrm{CaLHVA,dend,post}}$	$h_{\infty,\mathrm{CaLHVA,dend,post}}$	1	Gating variable for postsynaptic $\mathrm{Ca_{LHVA}}$ inactivation in the dendrite	
$h_{\mathrm{CaLLVA,dend,post}}$	$h_{\infty,\mathrm{CaLLVA,dend,post}}$	1	Gating variable for postsynaptic $\mathrm{Ca_{LLVA}}$ inactivation in the dendrite	
$h_{\mathrm{IP3R,post}}$	$h_{\infty,\mathrm{IP3R,post}}$	1	Gating variable for postsynaptic $\mathrm{IP_3R}$ inactivation	
$h_{\mathrm{KA,dend,post}}$	$h_{\infty,\mathrm{KA,dend,post}}$	1	Gating variable for postsynaptic $\mathrm{K_A}$ inactivation in the dendrite	
$h_{\mathrm{Na,dend,post}}$	$h_{\infty,\mathrm{Na,dend,post}}$	1	Gating variable for postsynaptic $\mathrm{Na^+}$ inactivation in the dendrite	
$h_{\mathrm{Na,soma,post}}$	$h_{\infty,\mathrm{Na,soma,post}}$	1	Gating variable for postsynaptic $\mathrm{Na^+}$ inactivation in the soma	
$m_{\mathrm{AMPAR,post}}$	0	1	Fraction of postsynaptic AMPARs in open state	
$m_{\mathrm{CaLHVA,dend,post}}$	$m_{\infty,\mathrm{CaLHVA,dend,post}}$	1	Gating variable for postsynaptic $\mathrm{Ca_{LHVA}}$ activation in the dendrite	
$m_{\mathrm{CaLLVA,dend,post}}$	$m_{\infty,\mathrm{CaLLVA,dend,post}}$	1	Gating variable for postsynaptic $\mathrm{Ca_{LLVA}}$ activation in the dendrite	
$m_{\mathrm{KA,dend,post}}$	$m_{\infty,\mathrm{KA,dend,post}}$	1	Gating variable for postsynaptic $\mathrm{K_A}$ activation in the dendrite	
$m_{\mathrm{KDR,soma,post}}$	$m_{\infty,\mathrm{KDR,soma,post}}$	1	Gating variable for postsynaptic $\mathrm{K_{DR}}$ activation in the soma	
$m_{\mathrm{Na,dend,post}}$	$m_{\infty,\mathrm{Na,dend,post}}$	1	Gating variable for postsynaptic $\mathrm{Na^+}$ activation in the dendrite	
$m_{\mathrm{Na,soma,post}}$	$m_{\infty,\mathrm{Na,soma,post}}$	1	Gating variable for postsynaptic $\mathrm{Na^+}$ activation in the soma	
$m_{\mathrm{NMDAR,post}}$	0	1	Fraction of postsynaptic NMDARs in open state	

Table W. Intermediate variables of astrocyte model.

Intermediate variable	Description	Refs
$h_{\infty,\mathrm{astro}} = \dfrac{Q_{\mathrm{astro}}}{Q_{\mathrm{astro}} + [\mathrm{Ca^{2+}}]_{\mathrm{astro}}}$	Steady-state value of astrocytic $\mathrm{IP_3R}$ inactivation gating variable	[18,19]
$m_{\infty,\mathrm{astro}} = \dfrac{[\mathrm{IP_3}]_{\mathrm{astro}}}{K_{\mathrm{IP3,1,astro}} + [\mathrm{IP_3}]_{\mathrm{astro}}}$	Steady-state value of astrocytic $\mathrm{IP_3R}$ activation gating variable	[18,19]
$n_{\infty,\mathrm{astro}} = \dfrac{[\mathrm{Ca^{2+}}]_{\mathrm{astro}}}{K_{\mathrm{act,astro}} + [\mathrm{Ca^{2+}}]_{\mathrm{astro}}}$	Steady-state value of astrocytic $\mathrm{IP_3R}$ activation gating variable	[18,19]
$J_{\mathrm{IP3R,astro}} = v_{\mathrm{IP3R,astro}} m^3_{\infty,\mathrm{astro}} n^3_{\infty,\mathrm{astro}} h^3_{\mathrm{astro}} \left(\mathrm{Ca_{tot,astro}} - (1 + r_{\mathrm{ERcyt,astro}}) [\mathrm{Ca^{2+}}]_{\mathrm{astro}}\right)$	$\mathrm{Ca^{2+}}$ flux via astrocytic $\mathrm{IP_3R}$	[18,19]
$J_{\mathrm{leakER,astro}} = r_{\mathrm{leakER,astro}} \left(\mathrm{Ca_{tot,astro}} - (1 + r_{\mathrm{ERcyt,astro}}) [\mathrm{Ca^{2+}}]_{\mathrm{astro}}\right)$	Astrocytic $\mathrm{Ca^{2+}}$ leak flux from ER to cytosol	[18,19]
$J_{\mathrm{SERCA,astro}} = v_{\mathrm{SERCA,astro}} \dfrac{[\mathrm{Ca^{2+}}]^2_{\mathrm{astro}}}{K^2_{\mathrm{SERCA,astro}} + [\mathrm{Ca^{2+}}]^2_{\mathrm{astro}}}$	$\mathrm{Ca^{2+}}$ flux via astrocytic SERCA pump	[18,19]
$Q_{\mathrm{astro}} = K_{\mathrm{inh,astro}} \dfrac{[\mathrm{IP_3}]_{\mathrm{astro}} + K_{\mathrm{IP3,1,astro}}}{[\mathrm{IP_3}]_{\mathrm{astro}} + K_{\mathrm{IP3,2,astro}}}$	Astrocytic intermediate variable	[18,19]
$\tau_{h,\mathrm{astro}} = \dfrac{1}{r_{\mathrm{IP3R,astro}} (Q_{\mathrm{astro}} + [\mathrm{Ca^{2+}}]_{\mathrm{astro}})}$	Time constant for astrocytic $\mathrm{IP_3R}$ inactivation	[18,19]

Table X. Parameter values of astrocyte model.

Parameter	Value	Unit	Description	Refs
$AG^{\star}_{post}$	0.0010453	µM	Postsynaptic resting 2-AG concentration	
$C_{thr,astro}$	0.3	µM	Ca^{2+} threshold concentration of Glu exocytosis in astrocytes	[59]
$Ca_{tot,astro}$	2	µM	Total free astrocytic Ca^{2+} concentration	[18]
G_{astro}	50,000	µM	Glu concentration per astrocytic vesicle	[9]
$IP^{\star}_{3,astro}$	0.28	µM	Astrocytic resting IP_3 concentration	[38]: 0.16 µM
$k_{recov,astro}$	0.0006	$\frac{1}{ms}$	Astrocytic recovery rate constant from empty to releasable state	[9]
$K_{act,astro}$	0.08234	µM	Astrocytic IP_3R dissociation constant of Ca^{2+} (activation)	[18]
$K_{inh,astro}$	1.049	µM	Astrocytic IP_3R dissociation constant of Ca^{2+} (inhibition)	[18]
$K_{IP3,1,astro}$	0.13	µM	Astrocytic IP_3R dissociation constant of IP_3	[18]
$K_{IP3,2,astro}$	0.9434	µM	Astrocytic IP_3R dissociation constant of IP_3	[18]
$K_{SERCA,astro}$	0.1	µM	Half-activation constant of astrocytic SERCA pump	[18]
N_{astro}	4	1	Number of readily releasable astrocytic vesicles	[9]
$P_{rel,astro}$	0.6	1	Basal release probability of astrocytic Glu vesicles	[9, 10]
r_{astro}	0.005	$\frac{1}{ms}$	Glu clearance rate from extrasynaptic space	[10]
$r_{ERcyt,astro}$	0.185	1	Ratio between astrocytic ER and cytosol volumes	[18]
$r_{IP3,astro}$	0.0008	$\frac{1}{ms}$	Rate constant of astrocytic IP_3 production	[38]
$r_{IP3R,astro}$	0.0002	$\frac{1}{\mu M\ ms}$	Astrocytic IP_3R binding constant for Ca^{2+} (inhibition)	[18]
$r_{leakER,astro}$	$\frac{J_{SERCA,astro} - J_{IP3R,astro}}{Ca_{tot,astro} - (1 + r_{ERcyt,astro})\,[Ca^{2+}]_{astro}}$	$\frac{1}{ms}$	Astrocytic ER leak parameter calculated at resting concentrations	[18]
$r_{vesext,astro}$	0.00065	1	Ratio between astrocytic vesicular volume and volume of extrasynaptic space	[9, 10]
$\tau_{IP3,astro}$	7,000	ms	Time constant for astrocytic IP_3 degradation	[38]
$v_{IP3R,astro}$	0.006	$\frac{1}{ms}$	Maximum rate of Ca^{2+} release via astrocytic IP_3R	[18]
$v_{SERCA,astro}$	0.0007	$\frac{\mu M}{ms}$	Maximum rate of Ca^{2+} uptake by astrocytic SERCA pump	[18]: 0.0009 $\frac{\mu M}{ms}$

Table Y. Initial values of astrocytic variables.

Variable	Initial value	Unit	Description
$[Ca^{2+}]_{astro}$	0.15002	µM	Astrocytic Ca^{2+} concentration
$[Glu]_{extsyn}$	0	µM	Concentration of Glu in extrasynaptic space
h_{astro}	$h_{\infty,astro}$	1	Gating variable for astrocytic IP_3R inactivation
$[IP_3]_{astro}$	0.28	µM	Astrocytic IP_3 concentration
$R_{rel,astro}$	1	1	Fraction of releasable astrocytic vesicles

References

1. Safiulina VF, Caiati MD, Sivakumaran S, Bisson G, Migliore M, Cherubini E. Control of GABA release at single mossy fiber-CA3 connections in the developing hippocampus. Front Synaptic Neurosci. 2010;2:1. doi:10.3389/neuro.19.001.2010.

2. Lavzin M, Rapoport S, Polsky A, Garion L, Schiller J. Nonlinear dendritic processing determines angular tuning of barrel cortex neurons *in vivo*. Nature. 2012;490(7420):397–401. doi:10.1038/nature11451.

3. Erreger K, Dravid SM, Banke TG, Wyllie DJA, Traynelis SF. Subunit-specific gating controls rat NR1/NR2A and NR1/NR2B NMDA channel kinetics and synaptic signalling profiles. J Physiol. 2005;563(2):345–358. doi:10.1113/jphysiol.2004.080028.

4. Clarke RJ, Johnson JW. Voltage-dependent gating of NR1/2B NMDA receptors. J Physiol. 2008;586(23):5727–5741. doi:10.1113/jphysiol.2008.160622.

5. Fiala JC, Grossberg S, Bullock D. Metabotropic glutamate receptor activation in cerebellar Purkinje cells as substrate for adaptive timing of the classically conditioned eye-blink response. J Neurosci. 1996;16(11):3760–3774. doi:10.1523/JNEUROSCI.16-11-03760.1996.

6. Tsodyks MV, Markram H. The neural code between neocortical pyramidal neurons depends on neurotransmitter release probability. Proc Natl Acad Sci USA. 1997;94(2):719–723. doi:10.1073/pnas.94.2.719.

7. Tsodyks M, Pawelzik K, Markram H. Neural networks with dynamic synapses. Neural Comput. 1998;10(4):821–835. doi:10.1162/089976698300017502.

8. Lee CCJ, Anton M, Poon CS, McRae GJ. A kinetic model unifying presynaptic short-term facilitation and depression. J Comput Neurosci. 2009;26(3):459–473. doi:10.1007/s10827-008-0122-6.

9. De Pittà M, Volman V, Berry H, Ben-Jacob E. A tale of two stories: astrocyte regulation of synaptic depression and facilitation. PLoS Comput Biol. 2011;7(12):e1002293. doi:10.1371/journal.pcbi.1002293.

10. De Pittà M, Brunel N. Modulation of synaptic plasticity by glutamatergic gliotransmission: a modeling study. Neural Plast. 2016;2016:7607924. doi:10.1155/2016/7607924.

11. Reuveni I, Friedman A, Amitai Y, Gutnick MJ. Stepwise repolarization from Ca^{2+} plateaus in neocortical pyramidal cells: evidence for nonhomogeneous distribution of HVA Ca^{2+} channels in dendrites. J Neurosci. 1993;13(11):4609–4621. doi:10.1523/JNEUROSCI.13-11-04609.1993.

12. Markram H, Muller E, Ramaswamy S, Reimann MW, Abdellah M, Sanchez CA, et al. Reconstruction and simulation of neocortical microcircuitry. Cell. 2015;163(2):456–492. doi:10.1016/j.cell.2015.09.029.

13. Avery RB, Johnston D. Multiple channel types contribute to the low-voltage-activated calcium current in hippocampal CA3 pyramidal neurons. J Neurosci. 1996;16(18):5567–5582. doi:10.1523/JNEUROSCI.16-18-05567.1996.

14. Sarid L, Bruno R, Sakmann B, Segev I, Feldmeyer D. Modeling a layer 4-to-layer 2/3 module of a single column in rat neocortex: interweaving *in vitro* and *in vivo* experimental observations. Proc Natl Acad Sci USA. 2007;104(41):16353–16358. doi:10.1073/pnas.0707853104.

15. Pinsky PF, Rinzel J. Intrinsic and network rhythmogenesis in a reduced Traub model for CA3 neurons. J Comput Neurosci. 1994;1(1):39–60. doi:10.1007/BF00962717.

16. Destexhe A, Mainen ZF, Sejnowski TJ. Kinetic models of synaptic transmission. In: Koch C, Segev I, editors. Methods in neuronal modeling. Cambridge, MA: MIT Press; 1998. p. 1–25.

17. Kim B, Hawes SL, Gillani F, Wallace LJ, Blackwell KT. Signaling pathways involved in striatal synaptic plasticity are sensitive to temporal pattern and exhibit spatial specificity. PLoS Comput Biol. 2013;9(3):e1002953. doi:10.1371/journal.pcbi.1002953.

18. De Young GW, Keizer J. A single-pool inositol 1,4,5-trisphosphate-receptor-based model for agonist-stimulated oscillations in Ca^{2+} concentration. Proc Natl Acad Sci USA. 1992;89(20):9895–9899. doi:10.1073/pnas.89.20.9895.

19. Li YX, Rinzel J. Equations for $InsP_3$ receptor-mediated $[Ca^{2+}]_i$ oscillations derived from a detailed kinetic model: a Hodgkin-Huxley like formalism. J Theor Biol. 1994;166(4):461–473. doi:10.1006/jtbi.1994.1041.

20. Wagner J, Fall CP, Hong F, Sims CE, Allbritton NL, Fontanilla RA, et al. A wave of IP_3 production accompanies the fertilization Ca^{2+} wave in the egg of the frog, Xenopus laevis: theoretical and experimental support. Cell Calcium. 2004;35(5):433–447. doi:10.1016/j.ceca.2003.10.009.

21. Blackwell KT. Calcium waves and closure of potassium channels in response to GABA stimulation in Hermissenda type B photoreceptors. J Neurophysiol. 2002;87(2):776–792. doi:10.1152/jn.00867.2000.

22. Zachariou M, Alexander SPH, Coombes S, Christodoulou C. A biophysical model of endocannabinoid-mediated short term depression in hippocampal inhibition. PLoS ONE. 2013;8(3):e58926. doi:10.1371/journal.pone.0058926.

23. Wade J, McDaid L, Harkin J, Crunelli V, Kelso S. Self-repair in a bidirectionally coupled astrocyte-neuron (AN) system based on retrograde signaling. Front Comput Neurosci. 2012;6:76. doi:10.3389/fncom.2012.00076.

24. Banerjee A, González-Rueda A, Sampaio-Baptista C, Paulsen O, Rodríguez-Moreno A. Distinct mechanisms of spike timing-dependent LTD at vertical and horizontal inputs onto L2/3 pyramidal neurons in mouse barrel cortex. Physiol Rep. 2014;2(3):e00271. doi:10.1002/phy2.271.

25. Banerjee A, Larsen RS, Philpot BD, Paulsen O. Roles of presynaptic NMDA receptors in neurotransmission and plasticity. Trends Neurosci. 2016;39(1):26–39. doi:10.1016/j.tins.2015.11.001.

26. Bouvier G, Larsen RS, Rodriguez-Moreno A, Paulsen O, Sjöström PJ. Towards resolving the presynaptic NMDA receptor debate. Curr Opin Neurobiol. 2018;51:1–7. doi:10.1016/j.conb.2017.12.020.

27. Badoual M, Zou Q, Davison AP, Rudolph M, Bal T, Fregnac Y, et al. Biophysical and phenomenological models of multiple spike interactions in spike-timing dependent plasticity. Int J Neural Syst. 2006;16(2):79–97. doi:10.1142/S0129065706000524.

28. Cousin MA, Robinson PJ. The dephosphins: dephosphorylation by calcineurin triggers synaptic vesicle endocytosis. Trends Neurosci. 2001;24(11):659–665. doi:10.1016/S0166-2236(00)01930-5.

29. Heifets BD, Chevaleyre V, Castillo PE. Interneuron activity controls endocannabinoid-mediated presynaptic plasticity through calcineurin. Proc Natl Acad Sci USA. 2008;105(29):10250–10255. doi:10.1073/pnas.0711880105.

30. Rodríguez-Moreno A, González-Rueda A, Banerjee A, Upton AL, Craig MT, Paulsen O. Presynaptic self-depression at developing neocortical synapses. Neuron. 2013;77(1):35–42. doi:10.1016/j.neuron.2012.10.035.

31. Cottrell JR, Li B, Kyung JW, Ashford CJ, Mann JJ, Horvath TL, et al. Calcineurin Aγ is a functional phosphatase that modulates synaptic vesicle endocytosis. J Biol Chem. 2016;291(4):1948–1956. doi:10.1074/jbc.M115.705319.

32. Bender KJ, Allen CB, Bender VA, Feldman DE. Synaptic basis for whisker deprivation-induced synaptic depression in rat somatosensory cortex. J Neurosci. 2006;26(16):4155–4165. doi:10.1523/JNEUROSCI.0175-06.2006.

33. Nevian T, Sakmann B. Spine Ca^{2+} signaling in spike-timing-dependent plasticity. J Neurosci. 2006;26(43):11001–11013. doi:10.1523/JNEUROSCI.1749-06.2006.

34. Rodríguez-Moreno A, Paulsen O. Spike timing–dependent long-term depression requires presynaptic NMDA receptors. Nat Neurosci. 2008;11(7):744–745. doi:10.1038/nn.2125.

35. Rodríguez-Moreno A, Kohl MM, Reeve JE, Eaton TR, Collins HA, Anderson HL, et al. Presynaptic induction and expression of timing-dependent long-term depression demonstrated by compartment-specific photorelease of a use-dependent NMDA receptor antagonist. J Neurosci. 2011;31(23):8564–8569. doi:10.1523/JNEUROSCI.0274-11.2011.

36. Min R, Nevian T. Astrocyte signaling controls spike timing–dependent depression at neocortical synapses. Nat Neurosci. 2012;15(5):746–753. doi:10.1038/nn.3075.

37. Rodríguez-Moreno A, Banerjee A, Paulsen O. Presynaptic NMDA receptors and spike timing-dependent long-term depression at cortical synapses. Front Synaptic Neurosci. 2010;2:18. doi:10.3389/fnsyn.2010.00018.

38. Nadkarni S, Jung P. Spontaneous oscillations of dressed neurons: a new mechanism for epilepsy? Phys Rev Lett. 2003;91(26):268101. doi:10.1103/PhysRevLett.91.268101.

39. Manninen T, Havela R, Linne ML. Reproducibility and comparability of computational models for astrocyte calcium excitability. Front Neuroinform. 2017;11:11. doi:10.3389/fninf.2017.00011.

40. Manninen T, Aćimović J, Havela R, Teppola H, Linne ML. Challenges in reproducibility, replicability, and comparability of computational models and tools for neuronal and glial networks, cells, and subcellular structures. Front Neuroinform. 2018;12:20. doi:10.3389/fninf.2018.00020.

41. Manninen T, Havela R, Linne ML. Computational models for calcium-mediated astrocyte functions. Front Comput Neurosci. 2018;12:14. doi:10.3389/fncom.2018.00014.

42. Manninen T, Havela R, Linne ML. Computational models of astrocytes and astrocyte-neuron interactions: characterization, reproducibility, and future perspectives. In: De Pittà M, Berry H, editors. Computational Glioscience. Cham, Switzerland: Springer; 2019. p. 423–454.

43. Jourdain P, Bergersen LH, Bhaukaurally K, Bezzi P, Santello M, Domercq M, et al. Glutamate exocytosis from astrocytes controls synaptic strength. Nat Neurosci. 2007;10(3):331–339. doi:10.1038/nn1849.

44. Marchaland J, Calì C, Voglmaier SM, Li H, Regazzi R, Edwards RH, et al. Fast subplasma membrane Ca^{2+} transients control exo-endocytosis of synaptic-like microvesicles in astrocytes. Journal of Neuroscience. 2008;28(37):9122–9132. doi:10.1523/JNEUROSCI.0040-08.2008.

45. Rasooli-Nejad S, Palygin O, Lalo U, Pankratov Y. Cannabinoid receptors contribute to astroglial Ca^{2+}-signalling and control of synaptic plasticity in the neocortex. Phil Trans R Soc B. 2014;369(1654):20140077. doi:10.1098/rstb.2014.0077.

46. Schneggenburger R, Zhou Z, Konnerth A, Neher E. Fractional contribution of calcium to the cation current through glutamate receptor channels. Neuron. 1993;11(1):133–143. doi:10.1016/0896-6273(93)90277-X.

47. Burnashev N, Zhou Z, Neher E, Sakmann B. Fractional calcium currents through recombinant GluR channels of the NMDA, AMPA and kainate receptor subtypes. J Physiol. 1995;485(2):403–418. doi:10.1113/jphysiol.1995.sp020738.

48. Ascher P, Nowak L. The role of divalent cations in the N-methyl-D-aspartate responses of mouse central neurones in culture. J Physiol. 1988;399(1):247–266. doi:10.1113/jphysiol.1988.sp017078.

49. Gabbiani F, Cox SJ. Mathematics for Neuroscientists. London, United Kingdom: Academic Press; 2010.

50. Steuber V, Willshaw DJ. A biophysical model of synaptic delay learning and temporal pattern recognition in a cerebellar Purkinje cell. J Comput Neurosci. 2004;17(2):149–164. doi:10.1023/B:JCNS.0000037678.26155.b5.

51. Riveros N, Fiedler J, Lagos N, Munoz C, Orrego F. Glutamate in rat brain cortex synaptic vesicles: influence of the vesicle isolation procedure. Brain Res. 1986;386(1):405–408. doi:10.1016/0006-8993(86)90181-2.

52. Schikorski T, Stevens CF. Quantitative ultrastructural analysis of hippocampal excitatory synapses. J Neurosci. 1997;17(15):5858–5867. doi:10.1523/JNEUROSCI.17-15-05858.1997.

53. Harris KM, Sultan P. Variation in the number, location and size of synaptic vesicles provides an anatomical basis for the nonuniform probability of release at hippocampal CA1 synapses. Neuropharmacology. 1995;34(11):1387–1395. doi:10.1016/0028-3908(95)00142-S.

54. Huguenard JR, McCormick DA. Simulation of the currents involved in rhythmic oscillations in thalamic relay neurons. J Neurophysiol. 1992;68(4):1373–1383. doi:10.1152/jn.1992.68.4.1373.

55. Tewari S, Majumdar K. A mathematical model for astrocytes mediated LTP at single hippocampal synapses. J Comput Neurosci. 2012;33(2):341–370. doi:10.1007/s10827-012-0389-5.

56. Tewari SG, Majumdar KK. A mathematical model of the tripartite synapse: astrocyte-induced synaptic plasticity. J Biol Phys. 2012;38(3):465–496. doi:10.1007/s10867-012-9267-7.

57. Politi A, Gaspers LD, Thomas AP, Höfer T. Models of IP_3 and Ca^{2+} oscillations: frequency encoding and identification of underlying feedbacks. Biophys J. 2006;90(9):3120–3133. doi:10.1529/biophysj.105.072249.

58. Bourne JN, Harris KM. Balancing structure and function at hippocampal dendritic spines. Annu Rev Neurosci. 2008;31:47–67. doi:10.1146/annurev.neuro.31.060407.125646.

59. Pasti L, Zonta M, Pozzan T, Vicini S, Carmignoto G. Cytosolic calcium oscillations in astrocytes may regulate exocytotic release of glutamate. J Neurosci. 2001;21(2):477–484. doi:10.1523/JNEUROSCI.21-02-00477.2001.
